# ARTEM‐IS for ERP: Agreed Reporting Template for EEG Methodology—International Standard for Event‐Related Potential Experiments

**DOI:** 10.1111/psyp.70187

**Published:** 2025-12-08

**Authors:** Anđela Šoškić, Vanja Ković, Johannes Algermissen, Nastassja L. Fischer, Giorgio Ganis, Remi Gau, Faisal Mushtaq, Guiomar Niso, Robert Oostenveld, Dejan Pajić, Yuri G. Pavlov, Katarina Stekić, Yu‐Fang Yang, Suzy J. Styles

**Affiliations:** ^1^ Laboratory for Neurocognition and Applied Cognition, Faculty of Philosophy University of Belgrade Beograd Serbia; ^2^ Department of Experimental Psychology University of Oxford Oxford UK; ^3^ Centre for Research in Pedagogy and Practice, National Institute of Education Nanyang Technological University Singapore; ^4^ Singapore and Science of Learning in Education Centre, National Institute of Education Nanyang Technological University Singapore Singapore; ^5^ School of Psychology University of Plymouth Plymouth UK; ^6^ Brain Research and Imaging Centre (BRIC) University of Plymouth Plymouth UK; ^7^ Neuro Data Science ORIGAMI Laboratory, McConnell Brain Imaging Centre, Faculty of Medicine McGill University Montréal Quebec Canada; ^8^ School of Psychology University of Leeds Leeds UK; ^9^ Leeds NIHR Biomedical Research Centre Leeds UK; ^10^ Instituto Cajal, CSIC Madrid Spain; ^11^ Donders Institute for Brain, Cognition and Behaviour Radboud University Nijmegen Nijmegen the Netherlands; ^12^ NatMEG, Karolinska Institutet Stockholm Sweden; ^13^ Department of Psychology, Faculty of Philosophy University of Novi Sad Novi Sad Serbia; ^14^ Institute of Medical Psychology and Behavioral Neurobiology University of Tuebingen Tuebingen Germany; ^15^ Department of Psychology University of Würzburg Würzburg Germany; ^16^ Division of Experimental Psychology and Neuropsychology, Department of Education and Psychology Freie Universität Berlin Berlin Germany; ^17^ Psychology, School of Social Sciences Nanyang Technological University Singapore; ^18^ Centre for Research and Development on Learning (CRADLE) Nanyang Technological University Singapore

**Keywords:** ARTEM‐IS, event‐related potentials, good scientific practice, open science, reproducibility, transparency

## Abstract

The choices we make during the recording, preprocessing and analysis of event‐related potentials (ERP) data can affect study outcomes. As such, it is critical that they are transparently reported to allow for reproducibility. Yet, systematic reviews of reporting practices in the field have shown that journal articles often do not meet this goal and that existing reporting guidelines have not resulted in a sufficient improvement in reporting transparency. An easier workflow for transparently documenting pipelines used in regular journal articles is needed. The ARTEM‐IS (Agreed Reporting Template for EEG Methodology—International Standard) initiative is working towards addressing this issue by building dynamic, interactive web applications that support documenting information required by existing publication guidelines in the form of a standardized metadata template. Completing an ARTEM‐IS form results in a human‐reader‐friendly PDF or DOCX and a machine‐readable JSON summary of methodological information. This level of specificity surpasses conventional article methods sections, ensuring fewer omissions and ambiguities. These can be used as supplements to a publication, as a memory aid when writing a paper, or as records that allow easier metadata extraction. Here, we present the ARTEM‐IS for ERP, which supports describing a typical ERP study, including most of its core methodological aspects (study description, experimental design, hardware, data acquisition, pre‐processing, measurement, visualization, additional comments). We discuss the current functionalities of ARTEM‐IS for ERP, its development via a grassroots collaborative initiative, and potential extensions (e.g., including complex designs or statistical analyses). In doing so, we highlight how widespread adoption of ARTEM‐IS can benefit researchers, reviewers, and the broader scientific community by improving transparency, reducing reporting errors, and expediting rigorous replication efforts.

## Impact Statement

Transparent reporting of ERP methods is essential for reproducibility, yet journal articles often lack sufficient detail. ARTEM‐IS for ERP addresses this by providing a web‐based tool that generates systematic and thorough methodological summaries, in both human‐readable and machine‐readable versions. These can serve as supplements to publications, memory aids, or structured research report metadata records, benefiting researchers, reviewers, and the broader scientific community. By improving reporting precision, ARTEM‐IS enhances replicability and facilitates meta‐scientific research in EEG.

**Abbreviations**
ARTEM‐IS: Agreed Reporting Template for EEG Methodology – International Standard
BIDS: brain imaging data structure
COBIDAS: OHBM Committee on Best Practice in Data Analysis and Sharing
CSV: comma separated values
EEG: electroencephalography
EOG: electrooculography
ERN: error‐related negativity
ERP: event‐related potentials
ICA: independent component analysis
INCF: International Neuroinformatics Coordinating Facility
JSON: JavaScript Object Notation
MEEG: magneto‐ and electroencephalography
MEG: magnetoencephalography
MySQL: my structured query language
OHBM: OrganizationOrganisation for Human Brain Mapping
OSF: Open Science Foundation
PDF: Portable Document Format
PHP: PHP: Hypertext Preprocessor

## 1 Introduction

Recordings of brain activity and behavior in neuroscientific experiments result in very rich datasets. The often high spatial and temporal density of these datasets allows for a myriad of possible data processing and analysis pathways, sometimes known as the ‘garden of forking paths’ (Gelman and Loken 2013) as every analytical decision contributes to an ever‐increasing number of possible outcomes. This problem has been of growing concern for researchers using electroencephalography (EEG) and, more specifically, the event‐related potentials (ERP) technique (Šoškić et al. 2024; Neuro; Clayson et al. 2021; Trübutschek et al. 2024). In order to go from raw EEG data to ERPs, the data have to undergo a complex yet flexible sequence of preprocessing and analysis steps (referred to as a “pipeline”). For each of these steps, researchers have to make decisions, including how to filter and reference the raw data, which baseline correction to apply, and which statistical analysis to conduct.

Recent studies have shown that these decisions matter. Sandre et al. (2020) applied several different common implementations of a few preprocessing steps to the same ERP dataset and found that these choices impacted the observed effects of the error‐related negativity (ERN) component as well as its reliability. In another recent demonstration in the domain of the N400 family of ERP components, when one chain of 8 pre‐processing and analysis steps was varied by choosing between only two and four options per step, this resulted in 864 possible pre‐processing and analysis pipelines. The significance and size of some of the experimental effects were shown to fluctuate across 14 chosen pipelines (Šoškić et al. 2024). Furthermore, the selection of software toolboxes or versions may contribute to variations in results (Kabbara et al. 2023; Lefebvre et al. 2018). Choices in ERP methodology are thus very likely to be one explaining factor for inconsistencies in the literature and differences between original studies and replications.

Given the potentially profound consequences of choices made during preprocessing and analysis of ERP data on the results, it is critical that all choices are transparently reported to allow for reproducibility and replicability (Niso, Krol, et al. 2022). With the hope of facilitating transparent and complete reporting of methodological details, several ERP reporting guidelines have been published (Donchin et al. 1977; Keil et al. 2014; Pernet et al. 2020; Picton et al. 2000). The trend towards transparency in ERP and EEG research is part of a broader effort to increase transparency and reproducibility of the scientific record in the past two decades (e.g., Foster and Deardorff 2017; Larson and Moser 2017; Niso, Botvinik‐Nezer, et al. 2022; Niso, Krol, et al. 2022; Open Science Collaboration 2015; Wilkinson et al. 2016).

Transparent and detailed reporting of preprocessing and analysis decisions is crucial not only for scientific rigor but also from an ethical standpoint, especially in clinical research (Emanuel et al. 2000). The ethical principle of beneficence explicitly requires researchers to minimize the risk/benefit ratio for participants in their studies (e.g., National Commission for the Protection of Human Subjects of Biomedical and Behavioral Research 1979). When contradictory or ambiguous findings arise due to incomplete methodological reporting, researchers risk repeating experiments—sometimes across multiple labs—without effectively resolving the inconsistencies. This cycle can place an unnecessary burden on participants, as illustrated by repeated clinical trials that fail to clarify inconclusive evidence see (Van Dang 2020, for an example of this in cancer research).

Despite the availability of reporting guidelines and calls to enhance transparency, empirical findings suggest these efforts have not significantly improved the depth of methodological detail in ERP journal articles (Clayson et al. 2019; Šoškić 2021; see also Paul and Mani 2022). This persistent shortfall highlights a need for new strategies that can make comprehensive reporting both feasible and impactful.

## 2 Existing Efforts Towards Tools for Methodology Documentation

A pioneering effort towards improving the reporting of ERP studies through novel approaches was the magnetoencephalography (MEG) and EEG (jointly MEEG) publication guidelines by Keil et al. (2014), which featured a checklist/reminder of items to include when writing an MEEG paper, including ERP ones. The list contained 51 items focusing on different research steps, from hypotheses to figures and statistical analysis details. This checklist requires a yes/no answer to whether a certain detail has been reported or not. Even though this list provided useful instructions, systematic reviews have found that it has not had an immediate impact on the quality of documenting methodology in ERP papers before and after the publication of the checklist (Clayson et al. 2019; Šoškić 2021). One possible explanation is that pressure to publish fast in traditional journal articles with limited space can outweigh the perceived value of an additional checklist (Styles et al. 2021).

Another initiative has been launched by the Committee on Best Practices in Data Analysis and Sharing (COBIDAS) by the Organization for Human Brain Mapping (OHBM), which is developing guidelines for good practice for neuroimaging (Nichols et al. 2017; Voets et al. 2024) and MEEG (Pernet et al. 2020). These guidelines include checklists that are more detailed than the one offered by Keil et al. (2014). Noting the ever‐increasing complexity of analysis pipelines, Pernet et al. (2020) recognized that the need to describe pipelines thoroughly is in conflict with limited journal space, and they recommended preparing supplementary tables with details listed in their checklist. To make this easier, COBIDAS and a working group from the International Neuroinformatics Coordinating Facility (INCF) are working on a web application (eCOBIDAS) that would allow easier creation of such supplements (Gau et al. 2022). While the checklist by Keil et al. (2014) is primarily meant to be completed at the time of writing a manuscript, this tool can act as a diary or ‘lab notebook’ and help document analytic decisions (even if only planned or hypothetical) at several research stages (e.g., decisions can be documented during planning a study or a change can be documented when a new decision is made during data collection or analysis). This feature is known to provide benefits to checklist completion (Degani and Wiener 1993; Styles et al. 2021).

In addition to projects specifically focused on improving standardizing the documentation of methodological choices, other initiatives that aim at facilitating research transparency include an ERP pre‐registration template (Govaart et al. 2022), initiatives that aim at standardizing rather than documenting pipelines (e.g., Bigdely‐Shamlo et al. 2015; Gabard‐Durnam et al. 2018; Monachino et al. 2022; Rodrigues et al. 2021), and initiatives that aim at standardizing the sharing of data/metadata, such as BIDS (Gorgolewski et al. 2016; Pernet et al. 2019; Poldrack et al. 2024) and the BIDS‐compliant data sharing platform OpenNeuro (Markiewicz et al. 2021). The pre‐registration template can help researchers consider, make and document methodological decisions before conducting a study. However, although pre‐registrations are a useful reminder of the study's decision‐making process, they do not require standardized documentation of methodological decisions and they are not aimed at documenting what eventually happened when the research has been conducted. Similarly, standardized data pre‐processing pipelines are not study documentation tools, but using an established pipeline might make it easier to describe it and thus has the potential for improved reproducibility compared to idiosyncratic pipelines (e.g., Rodrigues et al. 2021). Finally, data and metadata sharing standards such as BIDS help preserve very detailed and systematically curated methodological information in a metadata file accompanying a dataset. Such metadata can provide very rich information on the study, for example, details concerning individual participants or trials. However, creating well‐curated metadata and code describing complex pipelines, typically conducted using multiple software programs, can be demanding. Furthermore, standardized metadata files document the data end product, but not the methodological decisions (and rationales for these decisions) that eventually lead to this data. Even when they are accompanied by the pre‐processing and analysis code, some of the decisions and particularly the rationale for making them are still not preserved. In addition, extracting all desired information stored in this form is time‐consuming for reviewers and other researchers.

Such metadata can provide very rich information on the study; for example, details concerning individual participants or trials. However, creating well‐curated metadata and code describing complex pipelines, typically conducted using multiple software programs, can be demanding.

## 3 ARTEM‐IS: A Methodology Documentation Initiative

Taking into account the challenges described above, ARTEM‐IS (short for Agreed Reporting Template for EEG Methodology—International Standard) aims to provide tools that make documenting methodology easier and more transparent, and thus improve the reproducibility of research.

More specifically, the goal of the ARTEM‐IS initiative is to create ARTEM‐IS Templates: standardized reporting framework (“molds”) for EEG method summaries. An ARTEM‐IS Template allows systematically describing an EEG study from its design to data visualization, including the rationale for decisions made along the way. To make creating summaries structured in line with an ARTEM‐IS Template convenient, the secondary goal of the initiative is to develop web applications that facilitate this process.

**FIGURE 1:**
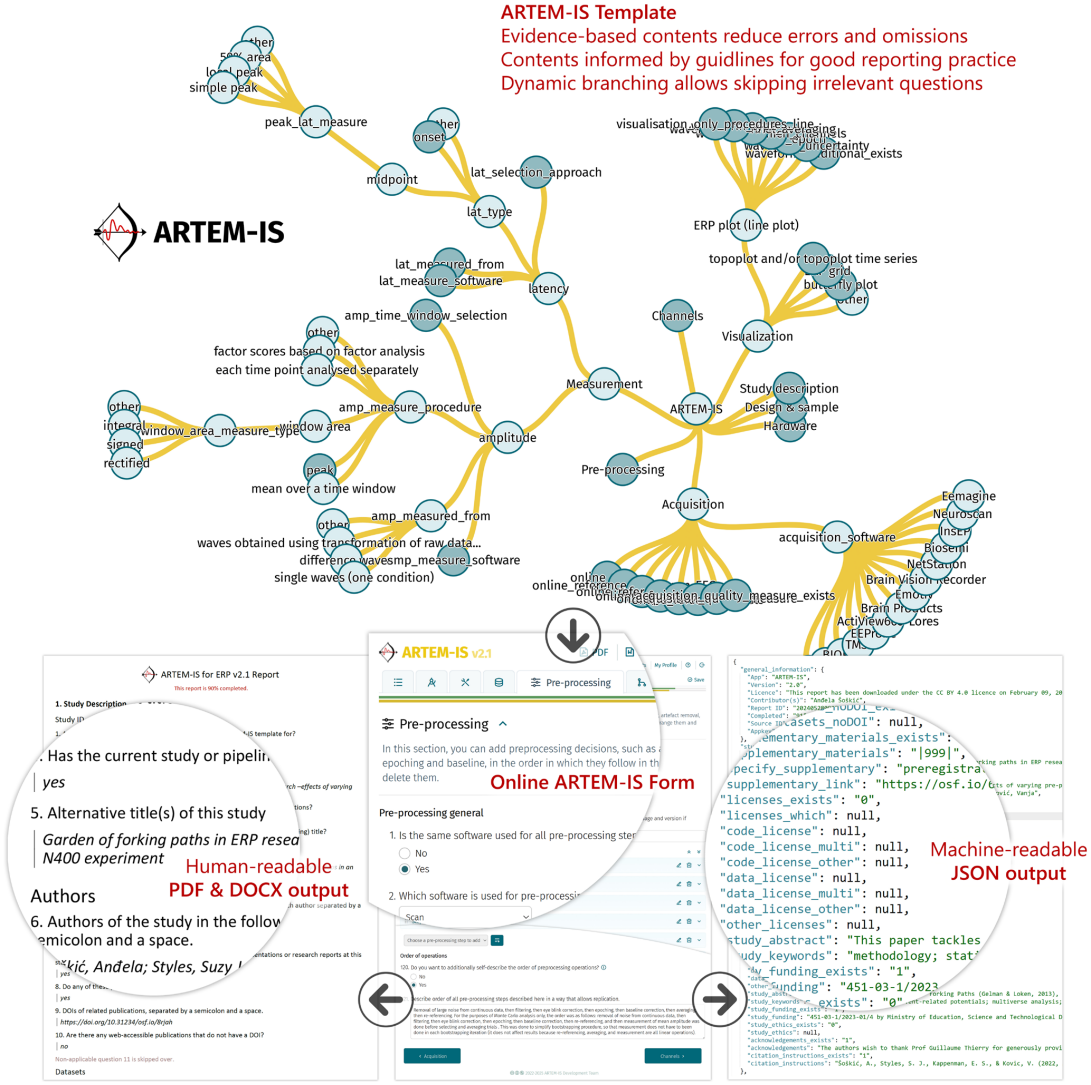
ARTEM‐IS for ERP concept summary.

From the perspective of an ARTEM‐IS Web Application user, creating an ARTEM‐IS‐compliant summary happens through filling in an online questionnaire (ARTEM‐IS Form), which is simple to use and requires short and specific answers, reducing reporting omissions and errors. Completing the Form generates an ARTEM‐IS Report (a summary of methodological information) that ensures a much higher level of compliance with good reporting practice recommendations than what is typically found in journal articles. Outputs of the ARTEM‐IS Web Application can be stored online and downloaded as human‐reader‐friendly PDF and DOCX files, and/or as machine‐readable JSON documents (see Figure 1). ARTEM‐IS Reports can be used as supplements to a publication, as a memory aid when writing a paper, or as records that allow easier metadata extraction in comparison to verbal descriptions in papers.

There are two main reasons to provide a form with individual data fields to be filled in rather than a yes/no checklist of whether a detail has been reported which researchers can refer to while writing their journal articles, such as the ones proposed by Keil et al. (2014) and Pernet et al. (2020). First, a questionnaire with data fields allows documenting the decisions as they are being made. As mentioned in the previous section, the main advantage of this approach is minimizing errors and omissions that occur when trying to reconstruct undocumented details during the paper write‐up, often long after the decisions were made. In addition, using the tool as a ‘diary’ or ‘lab notebook’ to document methodological decisions at any stage of the research process (e.g., while planning it, while conducting data collection) allows ARTEM‐IS to serve for documenting pre‐registration plans, studies in progress, as well as completed studies. Using the template prior to conducting a study can be especially helpful to researchers who are new to EEG, but it can also serve as a useful reminder for seasoned researchers. It can also be used to document and share internal lab standards, with new researchers building upon and extending a pipeline started by more senior researchers. Second, a remetadata form helps prevent omissions caused by mistakenly marking an incomplete or skipped item as complete in a yes/no checklist (Styles et al. 2021).

Documenting a study in enough detail for replication and metascience may sound time consuming at first. However, unlike traditional guidelines and checklists, ARTEM‐IS does not show a static and exhaustive list of steps researchers might potentially consider. Instead, the online web application has a dynamic branching structure that mirrors the decision‐making process itself by presenting questions contingent on previous answers and skipping irrelevant paths, significantly reducing workload. Moreover, most questions require selecting predefined options or inputting short answers. This not only facilitates completing the form, but also comparison between different studies. Third, once filled in, information on features such as EEG equipment can be used as a template for future study descriptions, shortening the process of study documenting even further, especially since, in many cases, researchers create a pipeline from scratch only once and then, in future projects, adjust or extend it.

In addition, investing time in detailed documentation yields long‐term benefits for various stakeholders, including researchers, collaborators (especially in large‐scale projects), reviewers, readers, and the broader scientific community. Examples of these benefits include more accurate pre‐registrations; easier and more frequent replication attempts, meta‐analytic projects, and large‐scale collaborations; streamlined report evaluations for reviewers, editors, and readers (potentially even semi‐automated in the future); fewer errors in methodological descriptions in the literature; reduced requests for additional information from corresponding authors; and improved understanding of the sources of inconsistencies in the literature.

When designing Templates, ARTEM‐IS relies on three cornerstone resources: (1) ARTEM‐IS Design Guidelines, which have been developed by leveraging expertise from other fields with a longer history of using checklists, such as surgery and aviation; (2) reporting standards in the field (e.g., Keil et al. 2014; Pernet et al. 2020; Styles et al. 2021); and (3) insights into reporting errors and omissions common in the EEG literature, which are obtained using detailed systematic reviews of methodology reporting (e.g., Šoškić 2021). The general ARTEM‐IS approach has been discussed in detail earlier (Styles et al. 2021).

## 4 ARTEM‐IS for ERP: The First ARTEM‐IS Documenting Tool

The main topic of the present paper is to describe the first ARTEM‐IS tool: ARTEM‐IS for ERP, designed for documenting a study on event‐related potentials. The up‐to‐date version of the web app can be accessed at http://artemis.incf.org/, which is maintained at https://github.com/INCF/artem‐is. Additional resources, including a user manual as well as a detailed and more technical description of the ARTEM‐IS Template and Web Application are available on the OSF page dedicated to ARTEM‐IS for ERP (https://osf.io/ahp3t/).

In the following sections, we will summarize ARTEM‐IS for ERP Template contents, current Web Application functionalities, efforts towards version compatibility and sustainability of the tool, an account of how ARTEM‐IS for ERP was developed, and the tool's current limitations.

### 4.1 ARTEM‐IS for ERP Template

The current version 2.1 of the ARTEM‐IS^1^ for ERP Template comprises nine sections designed to assist researchers in reporting the following aspects of a study: (1) study description, (2) experimental design and sample, (3) hardware, (4) acquisition, (5) pre‐processing, (6) measurement, (7) channel selection for analysis, (8) visualization, and (9) other. The description of the statistical analysis is not included in this version. Each section contains a set of questions; the answers can be categorical, numerical or short open‐ended textual statements. Sections are separated into subsections for easier navigation. While some questions are intended to be completed in all reports, the need to answer other questions is contingent on responses to previous ones.

Each section is briefly described below.

1. *Study*: This section gathers information about general aspects of the research study: information related to whether a Report documents a planned pipeline or an already applied one; the title of the study; information about the authors; related publications and DOIs; associated available datasets and Supporting Information (e.g., code); Supporting Information; licensing; abstract; keywords; financial support; ethics committee approval; acknowledgements; and citation instructions.
2. *Experimental design*: This section gathers information about the experimental design and the sample of the study: the number of participants in the study; inclusion and exclusion criteria; information related to the experimental/comparison groups; details on the trials presented and analyzed; and software for stimuli presentation.
3. *Hardware*: This section provides information about the hardware used for data acquisition: information related to the EEG cap/net; electrode characteristics and placement scheme; information on the amplifier and configuration; description of triggers (e.g., how they were generated and saved); and information related to any additional devices used (e.g., signal boxes, converters, electrode position measurement devices).
4. *Acquisition*: This section documents information related to the data acquisition process: acquisition software used and version; details on impedances or alternative data acquisition quality measures; references for EEG, EOG and other electrodes, if used, and their alternatives (e.g., Driven Right Leg (DRL)); ground electrode and placement; EOG channels for recording eye movement‐dependent voltage and placement; online high‐pass and low‐pass filters and notch filters; and acquisition sampling rate.
5. *Pre‐processing*: This section documents pre‐processing, i.e., software used for pre‐processing, the steps in the pipeline before measurement and statistical analysis of an ERP component: automated preprocessing pipeline; offline filtering; downsampling; re‐referencing of EEG, EOG and other channels, if used; artifact removal methods in each elimination step (rejection of bad trials, data segments, or channels, artifact correction, channel interpolation, multi‐step automated approaches); epoching; baseline correction; other steps. The user can add as many steps as they want in the order they are arranged in the pipeline and a t the end of the section, the user is asked whether they would like to additionally self‐describe the order of operations in a free‐text field.
6. *Channels*: This section gathers information about the selection of channels for later statistical analysis. Like in the case of the previous section, the current version of the template supports describing the location for the measurement of one ERP component. There are two main scenarios– all channels are included in statistical analyses (e.g., in a mass univariate approach), or a subset of channels is included in the analyses. Within the second scenario, five options are offered: a priori selection of channels; data‐driven selection of channels from the entire scalp; data‐driven selection of channels from an a priori selected broader region on the scalp; selection/identification of channels in two data‐driven steps (e.g., maximal effect within a visually identified broader region); other. Depending on the choice, an appropriate subset of questions follows. At the end of this section, the user is asked to describe whether the channels were entered separately into statistical analysis or if they were aggregated into regions before conducting the statistical analysis.
7. *Measurements*: This section offers options to describe amplitude and latency measurements of one ERP component, unless the user subjected all time points and channels to statistical analysis (e.g., in a mass univariate analysis). Amplitude properties include: measurement software, waveforms used to measure amplitude, amplitude measure (peak, mean, window area, etc.) and its parameters, measurement time window, rationale for selecting this exact time window. Similarly, latency properties include: measurement software, waveforms used to measure latency, midpoint latency measure (e.g., local peak, 50% area)—if applied, onset latency measure (e.g., fractional area, fractional peak)—if applied, the appropriate parameters of each latency measure depending on the choices, time window within which the latency was searched for, and the rationale for selecting this exact time window.
8. *Visualization*: This section provides information related to the visualization: the type of plot created (e.g., line plot (waveforms), topoplot, ERP grid, butterfly plot); whether any extra pre‐processing was performed for visualization purposes (e.g., smoothing filter, different baseline); description of what the data represents (e.g., single waves, difference waves) and corresponding units (e.g., voltage maps, normalized voltage maps); description of which conditions or difference waves were shown; channels selected for visualization and the rationale for this decision, beginning and end of the time window visualized and the rationale for selecting this time window.
9. *Other*: This section contains an option to add additional comments, if there are any.

**FIGURE 2:**
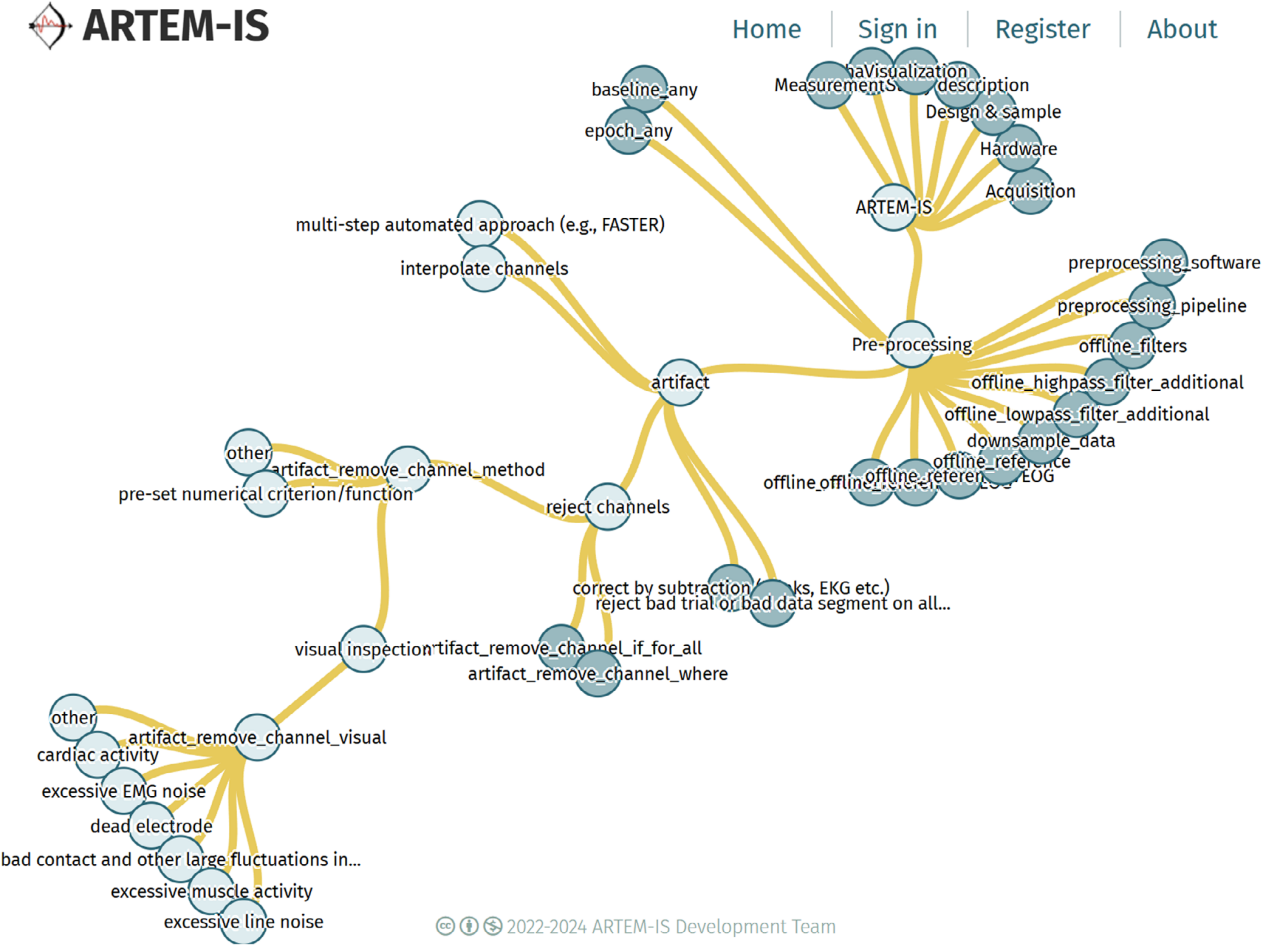
Part of the branching structure of ARTEM‐IS for ERP reporting items. Nodes at the first level of branching represent sections, while other nodes represent either multiple‐choice items (questions) or choices that can be picked.

A dynamic overview of the branching structure of multiple‐choice questions in the form is available on the front page of the web application (http://artemis.incf.org/), where it is visible before logging in, while an example of this branching can be seen in Figure 2.

The ARTEM‐IS for ERP v2.1 Template does not set rules on which items in the Template are mandatory to fill in. This is because the goal of the ARTEM‐IS project is not to enforce providing a particular set of information. Our goal, instead, is to help researchers be more detailed and precise in their descriptions, as well as to help readers have an easy and transparent overview of which (and how much) of the necessary information has been provided.

Notably, most questions that are part of the ARTEM‐IS for ERP v2.1 Template are described as necessary to provide in a research report by contemporary guidelines for good practice in EEG research (e.g., Keil et al. 2014; Pernet et al. 2020). There are a few exceptions, where the researchers who took part in the ARTEM‐IS Template development suggested additional items they considered useful (e.g., additional filter properties that researchers may want to provide on top of the minimal reporting guidelines). The Template clearly separates these items from the items listed in the reporting guidelines. In these cases, the researchers are first asked whether they would like to include this additional information, and only asked to provide it if they select “Yes”. If a researcher selects “No”, they skip these additional questions, which do not count towards the percent of completion calculated by the ARTEM‐IS Web Application.

### 4.2 ARTEM‐IS for ERP Template Specification

ARTEM‐IS for ERP Template is described using two data sheets which define reporting items that researchers should provide, as well as their properties, such as rules for their dynamic showing and hiding or the expected type of answer. The two data sheets jointly constitute an *ARTEM‐IS Template Specification*. The first data sheet, called simply *ARTEM‐IS for ERP v2*.*1 Spreadsheet*, contains all questions (reporting items) that can be found in this Template and specifies their properties, such as the metadata field name in the JSON Report for each question, logical expression defining the conditions under which the question is shown, or the type of input that is expected. The second data sheet is called *ARTEM‐IS for ERP v2.1 Presets* and it defines answers that should be offered to multiple‐choice questions.

The data sheets are edited on the Google Spreadsheets platform by the ARTEM‐IS team, which facilitates collaborative work with a low bar of entry for new contributors in terms of technical skills. A static version of the ARTEM‐IS for ERP v2.1 Specification is openly available on OSF (https://osf.io/ahp3t/), with a Creative Commons License (CC) with an obligation for attribution (BY), for non‐commercial uses (NC), and with the obligation that others will also share their resulting work with an equivalent license (SA) (i.e., CC‐BY‐NC‐SA). Educational uses such as in higher education or commercial training courses are specifically licensed.

### 4.3 ARTEM‐IS Web Application

Existing off‐the‐shelf software solutions were not suitable for providing a flexible and fully controlled environment to serve as a platform on which ARTEM‐IS would be built. Hence, the ARTEM‐IS team has opted for the development of a custom web application as an interface for the dynamic and interactive data framework. The application was created in PHP and JavaScript, using MySQL as a database backend, and it is hosted on the platform of the International Neuroinformatics Coordinating Facility (INCF, www.incf.org). Developing an application from scratch ensured the future upgrading and improvement of ARTEM‐IS for ERP, as well as building ARTEM‐IS tools for other subdomains of EEG research by modifying and adapting the database structure built for ARTEM‐IS for ERP.^2^

#### 4.3.1 Code License

The ARTEM‐IS Web Application is currently not open source, but the ARTEM‐IS team is working towards making it open in due course. Please note that the ARTEM‐IS Web App is intended to be a tool that facilitates creating and sharing ARTEM‐IS Reports. As such, it derives all of the essential functionality directly from the ARTEM‐IS Template Specifications, which are openly available as stated above, and which can be used to create and share ARTEM‐IS‐compliant reports independently of the Web App. At present, the only intellectual property that is not shared is the code for displaying the user interface and managing reports in the backend of the ARTEM‐IS official Web Application on the International Neuroinformatics Coordinating Facility (INCF) platform (https://artemis.incf.org/).

#### 4.3.2 Web Application Input

The main motivation behind the creation of the application was to enable users to easily generate ARTEM‐IS Reports using the guided interactive form. Questions are organized into sections, so that one can switch between them, not necessarily filling the questionnaire in a linear way. Changes to the Report are saved automatically, thus making it possible to document incomplete templates for research in progress. In line with the dynamic, branching structure of the template, many of the questions are associated with a logical expression that determines whether the question should be displayed or not, depending on the answers to previous questions.

In line with the decision not to make any questions mandatory to fill in, the ARTEM‐IS Web Application also does not enforce answering any questions in the Form. Each Report can be saved, downloaded, and publicly shared in any stage of the progress of filling out the Form. Instead, the readers of online Reports shown in the Web App and of the downloaded Reports are provided information on the percent of completion of a given Report. The calculation of this proportion is dynamically adapted to exclude items that are skipped due to the branching logic of the Template. In addition, users always have feedback on which questions still require response since they are clearly marked in red.

#### 4.3.3 Output: ARTEM‐IS Reports

ARTEM‐IS Reports can be viewed and edited online, as well as downloaded in three formats: PDF, DOCX, and JSON documents. A PDF Report is made to be easier to understand by human readers, with the full text of both the questions and answers displayed in the Report. The DOCX format offers the same content, but with a complementary purpose. While PDF offers consistent formatting and wider accessibility, DOCX is more suitable for copying and reusing content.

In contrast, JSON Reports are made with machine readability and version compatibility in mind. Structured JSON objects, unlike, for example, CSV files, provide a convenient way to store hierarchically organized, machine‐readable information as a set of key/value, i.e., question/response pairs. Additionally, JSON files are exported in the so‐called “pretty‐print” format which enables users to relatively easily search and browse through the template structure in a text editor. Finally, the JSON format provides a convenient way for users to export and import information and build their own Reports upon the already existing privately shared or public Reports (see Using an existing Report as a template for a new one).

To achieve these goals, Reports in JSON format feature item codes rather than full question text of the items or full text of response options for multiple‐choice questions. This is because the item codes for the same question can remain unchanged between versions of ARTEM‐IS in the case of minor changes such as slight rewording. Item codes for each question and response option can be found in the ARTEM‐IS Template Specification (see ARTEM‐IS for ERP Manual at https://osf.io/kn9f3/ for more details).

To help track editing history, reports both in all formats include information on the date of download, contributors, and license at the time of download. Similarly, online Reports include information on when they were edited and by whom, but the full version history is not preserved as this would require considerable additional memory resources. This makes Reports in PDF and JSON format more appropriate supplementary documents for journal articles than online Reports.

#### 4.3.4 Fostering Collaboration and Exchange

Besides allowing easier creation of ARTEM‐IS‐compliant Reports, the second motivation behind the development of the web application was to facilitate the exchange of information and collaboration among researchers. The application is free to use but requires user registration since the user is responsible for the decision on who would be able to see, download, and/or modify the template and under what conditions. Reports can be restricted to an invited group of contributors or distributed freely under the CC BY 4.0 license. Contributors to the report have the option to add new or existing ARTEM‐IS users to the team and invite them to collaborate on the project report. New collaborators automatically receive an email notification with the unique link to the ARTEM‐IS template form. Information on authors of a study is separate from the information on the contributors to its ARTEM‐IS report, allowing, for example, researchers to systematically document the method of papers that are published by others for the purposes of systematic reviews and meta‐analyses.

#### 4.3.5 Using an Existing Report as a Template for a New One

There are many scenarios when reusing contents already provided in an existing ARTEM‐IS Report can save considerable time. For example, laboratories may want to save their default acquisition settings in one place and prepare a template that only needs to be supplemented with details on a particular study. In an even more time‐saving scenario, many laboratories use standard designs and pipelines and can save significant time by creating pre‐filled reports with these standardized settings, which then only need to be completed with idiosyncratic information. A third use case is the replication or continuation of a study—if a study already has a publicly available ARTEM‐IS Report, authors of the replication or follow‐up study can copy the existing Report and modify it where deviations occur.

ARTEM‐IS for ERP Web Application allows the reuse of existing documents to build new ones either by copying the online Reports or by uploading Reports in JSON Format. The latter option allows reuse of not only publicly available Reports, but also the ones that are shared privately outside of the Web Application, for example if a lab manager privately shares a pre‐filled Report template with laboratory setup with their team members via email.

In both cases, the result is a *new online Report* with all answers pre‐filled/copied from the original report. The newly created online Report can have a completely different team of contributors compared to the original Report, and it is clearly marked as a *derivative* of the original report, with a link to the original source (while ensuring privacy protections for private originals).

### 4.4 Use Case Examples

In the section, a few examples from practice are provided, with scenarios that a typical laboratory may encounter. For simplicity, PDF documents are provided, but the reader can also find JSON files on the OSF page of ARTEM‐IS for ERP (https://osf.io/ahp3t/).

The most typical use case scenario for using ARTEM‐IS for ERP is to document an already conducted study or a study that is currently in progress. The study can be documented either as decisions are being made, or later, after it has been completed. Similarly, ARTEM‐IS can be used to document EEG settings for a planned study, for example as a supplement to a preregistration. Within our team, documenting an entire already conducted study with one ERP component in focus took between 30 and 60 min, depending on study complexity and the amount of effort to obtain information that is not immediately available. This applied even if a study had been conducted years ago. An example of documenting a study that has been conducted earlier can be found on OSF (https://osf.io/6tpk2).

In the next example, already briefly mentioned earlier, a PI or laboratory manager may want to create an ARTEM‐IS Report which contains the default EEG acquisition setup(s) within their laboratory or a standard design frequently used in the laboratory. In this case, an ARTEM‐IS Report can be used by the laboratory as a reference guide when conducting studies or writing articles, especially for members who are new to the lab. This way, laboratory members can save time looking up equipment details over and over again, or help ensure that studies using the same approach use consistent or time‐tested methods. In addition, such a half‐ready ARTEM‐IS Report can be used as a template to save time when building ARTEM‐IS Reports on studies that are conducted in the laboratory. When the PI or manager is familiar with the laboratory set‐up, creating a most basic template takes less than 15 min. If a new Report is made using a more detailed pre‐filled template, documenting a new study may take as little as 20 min. One example of this type of Report can be found on the OSF page of ARTEM‐IS for ERP (https://osf.io/dn6s4).

In the final example, the reader can see how ARTEM‐IS can be used to document multiple ERP components analyzed in the same study or multiverse analyses of the same dataset even though ARTEM‐IS for ERP v2.0 allows describing a single pre‐processing and analysis pipeline, including asking about one measurement window and one set of channels. The simple workaround for this current limitation of the Template is to copy a Report with one pipeline or component described and change the parameters that are different for the new component or pipeline. This has been done by Šoškić et al. (2024) for a multiverse analysis of the same ERP component and the resulting set of related ARTEM‐IS Reports can be found on their OSF project page (see Supplement D at https://osf.io/6nqxy/). In this example, creating additional Reports based on the first completed Report took about 10 additional minutes for each copy.

### 4.5 Version Compatibility and Sustainability

Two major concerns that are frequently expressed when it comes to projects similar to ARTEM‐IS are compatibility between different versions and sustainability of the project in the face of challenges such as changes in team composition.

Regarding the first challenge, starting from v2.0, ARTEM‐IS Template Specification is accompanied by a set of guidelines that ensure compatibility between versions and clear relations between different versions of the same template. These guidelines can be found in the *ARTEM‐IS Template and Web Application Description* for ARTEM‐IS for ERP v2.1, which can be found on our OSF page (https://osf.io/ahp3t/).

The second challenge is more difficult to address and is shared with many projects of a similar kind. One way that ARTEM‐IS tackles this challenge is by making the ARTEM‐IS Specification openly available and documented in detail, by committing in due course to make the Web Application code openly available in the same manner, too. This facilitates knowledge transfer and continuity, especially in cases where crucial team members are unable to continue their work on the project.

### 4.6 Limitations of ARTEM‐IS v2.1

The ARTEM‐IS for ERP is the first ARTEM‐IS tool, and as such, while it is fully functional, it also serves as a proof of concept. Namely, it currently contains several core features both regarding the Template contents and the Web Application functionalities, that can be used and tested by a broader community of users, and that can be further built upon.

In its current v2.1 version, the ARTEM‐IS for ERP Template allows documenting a simple ERP experiment involving one ERP component (one set of electrodes; latency and amplitude measurements on this set of electrodes and each from one time window) and one pre‐processing pipeline. A workaround for representing multiple pipelines and components has been discussed in the *Use case examples* section. Regarding the complexity of study design, ARTEM‐IS is currently best suited for simple study designs involving a limited number of experimental conditions and standard pre‐processing options, which are represented using predefined options. Complex and non‐standard designs and pre‐processing options can still be described using free‐text options, including the one at the very end of the Report, and the option to copy Reports can save considerable time. While not as convenient as a more simple use‐case scenario, it still allows capturing necessary details.

ARTEM‐IS for ERP v2.1 does not cover statistical analysis, which is itself so complex and varied that it could easily become a separate ARTEM‐IS Template. Until such a time, users may choose to report their statistical analysis in the “Other” section if there is a need. The web application design allows expanding its scope to include statistical analyses in the future.

Likewise, the Web Application currently supports managing only one version of one ARTEM‐IS Template. This has two notable consequences. Firstly, if there is an update to the ARTEM‐IS for ERP Template, all the Reports in the Web Application will be migrated to the new version of the Template. However, it is an important goal of the ARTEM‐IS project to keep versions of the same Template mutually compatible as much as possible. Secondly, if ARTEM‐IS extends to additional subfields of EEG, this will require additional Web Application development.

In addition, in the current version of the Template, some steps (e.g., artifact removal decisions that involve more complex algorithms and procedures, such as ICA) are still left to the user to describe in an open‐ended format. These can be expanded into a full branching structure of short questions to be fully compliant with ARTEM‐IS Guidelines in subsequent versions.

Finally, there is space for further enhancements and refinements as ERP methodology progresses and as we learn more from the community using the tool. For example, in future editions, new options may be added to categorical questions that now fit under the category of “Other”, or new questions may be added to include new developments in science or to break down existing open‐ended questions. In addition, the existing tool may be tweaked to increase accessibility and serve a pedagogical purpose for researchers new to ERP by preparing instructional materials, such as a lexicon of used terminology or video guides. One stretch goal of the project is to consider ARTEM‐IS as a pathway to software that suggests automatically generated text suitable for use in a methods section.

To validate the usability of ARTEM‐IS for ERP, the Template has been tested on about 40 studies, mostly published ones with classical ERP designs with visual and auditory stimuli and conducted on neurotypical young adults, though the sample of studies also includes examples featuring children, clinical samples, mobile EEG devices, complex designs, multiverse analyses, laboratory protocols. It was feasible to use ARTEM‐IS to represent all of these studies; though, as expected, the current version of ARTEM‐IS for ERP is not optimal for studies featuring complex designs (primarily designs with many conditions across all factors). ARTEM‐IS is yet to be tested on multimodal recordings, studies involving more diverse stimulus modalities, or mobile ERP studies involving the free movement of participants. Researchers conducting studies that do not feature simple and classical ERP experiments with neurotypical adults may want to briefly review the entire Template before deciding that it is compatible with their study, though we see no reason in principle why any ERP study would be impossible to describe using the Template.

### 4.7 Development Through Community Involvement

ARTEM‐IS for ERP has been developed as a collaborative grassroots initiative involving contributors from different backgrounds who were recruited at conferences, hackathons and via social media calls, and it is open for further improvements through scientific community collaboration. In line with the ARTEM‐IS Guidelines, ARTEM‐IS for ERP has been initially developed on the basis of the outcomes of the systematic review of ERP literature by Šoškić et al. (2021). Since then, it has been continually improved through the efforts of the INCF working group on ARTEM‐IS (https://www.incf.org/sig/incf‐working‐group‐artem) with contributions from a wider group of collaborators at hackathons (Gau et al. 2022), specifically OHBM BrainHack in July 2021 (https://github.com/ohbm/hackathon2021/issues/11) and BrainHack Global in December 2021 (https://brainhack.org/global2021/project/project_153/). In addition, ARTEM‐IS has benefited from exchanges with other open neuroscience projects, especially eCOBIDAS, whose work on representing neuroscientific reporting items as data fields editable in Google Spreadsheets served as a base for developing the ARTEM‐IS Specification. A full list of contributors is maintained on OSF (https://osf.io/ut9pc/). In addition, community feedback was requested through consultation opportunities at conferences, such as LiveMEEG 2020 (Šoškić et al. 2020), PuG (Šoškić 2021), SIPS (Yang and Styles 2022), INCF Assembly (Šoškić et al. 2022), SPR (Šoškić et al. 2022b), Neuronus (Šoškić et al. 2022a), or Methods in Mobile EEG (Stekić et al. 2023).

The benefits of community involvement become obvious when comparing the original draft of ARTEM‐IS for ERP to the current version. The original draft comprised a static spreadsheet table with 92 fields to be filled in, with options to add more detail next to each field. The current v2.1 version of ARTEM‐IS for ERP is a web application with 460 fields that facilitates dynamic and structured data entry and has all the features described above.

## 5 General Conclusions

ARTEM‐IS addresses the emerging needs of scholarly publications in the field of electrophysiology by offering EEG methodology metadata templates, which help researchers document methodological decisions from study design to data visualization in a reproducible and metascience‐friendly way. To ensure both quality of the tools and equity of access to them, ARTEM‐IS templates are developed with the input from the wider EEG community and are openly available to everyone. ARTEM‐IS for ERP is the first ARTEM‐IS tool, focused specifically on documenting studies using event‐related potentials (http://artemis.incf.org/).

Documenting an experiment to the level of detail sufficient for replication attempts and metascience has several benefits. For example, the ARTEM‐IS for ERP could potentially fast forward ERP reporting, as it can help researchers prepare the Methods section of manuscripts more easily and accurately. Not only reporting, but the review process could also be made easier, if journal editors ask researchers to attach the ARTEM‐IS template with article submission. This would allow reviewers to check more easily whether crucial steps in the reporting pipeline have been skipped, reported ambiguously, or performed in a manner inconsistent with other elements of the methodological description. In addition, improved reproducibility and replicability are in line with the ethical principle of beneficence, given that low reproducibility and replicability slow down the progress of science and put undue additional burden on participants. Another advantage of using ARTEM‐IS for ERP is that it allows researchers to outsource some methodology details to the supplementary template, saving more of the valuable journal space for results and discussion. Next, while guidelines for pre‐registering ERP studies exist (Paul and Mani 2022), ARTEM‐IS for ERP can make pre‐registration efforts more accurate due to the high level of precision it enables. Documenting study methodology before data collection is not only helpful for improving transparency, but also for practicing careful planning by reducing unnoticed flexibility. Similarly, when documenting completed studies, promoting good scientific practice (Niso, Botvinik‐Nezer, et al. 2022; Niso, Krol, et al. 2022; Ekhtiari et al. 2024) of sharing a detailed analysis pipeline will benefit both researchers interested in replications and the authors themselves when designing new studies based on previous findings (Garrett‐Ruffin et al. 2021). Finally, leveraging data from the ARTEM‐IS reporting template could inspire future replication studies and promote open science. Namely, there have not been many replication efforts in the field of EEG so far (Pavlov et al. 2021; Šoškić 2021), and one of the reasons may be that it is difficult to design replications without deep involvement of original authors, as initial steps in the #EEGManyLabs project have shown (Pavlov et al. 2021). Moreover, helping researchers achieve closer replications would allow opening up for discussions around replication results to be centered around theoretical questions rather than methodological differences.

In the current version, ARTEM‐IS for ERP v2.1 includes most of the core aspects of methods of a typical ERP study, and as such it is already ready to use for a broad class of typical ERP experiments. While we believe that this tool is already a big step forward in documenting methodological decisions in the field of ERP, it can be further improved by adding more sections and questions, primarily to allow documenting more complex designs and statistical analysis, but also by keeping up to date with progress and trends in ERP methodology. As with all previous developments, this work in progress is also open to input from the entire ERP community through contributions and feedback at future conferences and hackathons or joining the work of the INCF Working Group on ARTEM‐IS.

## Author Contributions

**Anđela Šoškić:** conceptualization, methodology, data curation, validation, visualization, writing – original draft, writing – review and editing, project administration. **Vanja Ković:** conceptualization, writing – original draft, methodology, validation, visualization, writing – review and editing, project administration, data curation. **Johannes Algermissen:** methodology, validation, writing – review and editing. **Nastassja L. Fischer:** methodology, validation, writing – original draft, writing – review and editing. **Giorgio Ganis:** methodology, validation, visualization, writing – original draft, writing – review and editing. **Remi Gau:** methodology, data curation, software, writing – review and editing. **Faisal Mushtaq:** project administration, writing – review and editing. **Guiomar Niso:** writing – review and editing, methodology, validation, writing – original draft. **Robert Oostenveld:** methodology, validation, writing – review and editing, writing – original draft. **Dejan Pajić:** methodology, writing – review and editing, conceptualization, software, visualization. **Yuri G. Pavlov:** methodology, validation, writing – original draft, writing – review and editing. **Katarina Stekić:** methodology, validation, software, writing – original draft, writing – review and editing. **Yu‐Fang Yang:** methodology, validation, writing – original draft, writing – review and editing, data curation. **Suzy J. Styles:** conceptualization, methodology, validation, visualization, project administration, writing – original draft, writing – review and editing, data curation.

## Conflicts of Interest

The authors declare no conflicts of interest.

## Supporting information


**Data S1:** psyp70187‐sup‐0001‐Supinfo1.pdf.

**Data S2:** psyp70187‐sup‐0002‐Supinfo2.pdf.

**Data S3:** psyp70187‐sup‐0003‐Supinfo3.json.

**Data S4:** psyp70187‐sup‐0004‐Supinfo4.pdf.

**Data S5:** psyp70187‐sup‐0005‐Supinfo5.json.

**Data S6:** psyp70187‐sup‐0006‐Supinfo6.pdf.

**Data S7:** psyp70187‐sup‐0007‐Supinfo7.xlsx.

**Data S8:** psyp70187‐sup‐0008‐Supinfo8.xlsx.

## Data Availability

ARTEM‐IS for ERP is maintained at https://github.com/INCF/artem‐is and additional Supporting Information can be found at https://osf.io/ahp3t/.

## Endnotes

^1^
v1.0 was a pilot version of the first usable template, designed to gather feedback based on an already developed model. Based on this feedback, v2.0, submitted for review in this journal, introduced major improvements, primarily restructuring the *template specification* into two documents to enable advanced encoding of questions and responses, ensuring compatibility between report versions—one of v1.0's main limitations. However, these changes made the two versions incompatible, particularly due to modifications in numerical response codes. Additionally, v2.0 expanded in terms of content, with the most significant updates in visualization. Importantly, v1.0 was never used in practice, so this incompatibility is not an issue. The current version, v2.1, which incorporates also the reviewer feedback, is fully compatible with v2.0.

^2^
There are existing, open‐source, off‐the‐shelf software solutions for questionnaire design, and some of them would make ARTEM‐IS more compatible with the similar attempts in the neuroimaging field, in the first place eCOBIDAS, with which ARTEM‐IS shares the goal to create reporting tools, and which very generously supported the ARTEM‐IS team in designing the initial versions of the Web Application. Unfortunately, none of these solutions is currently flexible enough to offer all features needed for the ARTEM‐IS Web Application. The niche requirements of the ARTEM‐IS project compared to a typical questionnaire forced us to develop a custom solution, while trying to keep the ARTEM‐IS Template Specification as similar as possible to eCOBIDAS to facilitate collaboration.
