# Supplementary material for: ARTEM‐IS for ERP: Agreed Reporting Template for EEG Methodology—International Standard for Event‐Related Potential Experiments
Source: Psychophysiology. 2025 Dec 8;62(12):e70187. doi: 10.1111/psyp.70187 (PMC12683983; doi:10.1111/psyp.70187)
Supplement: Supplementary file 1 — Data S1: psyp70187‐sup‐0001‐Supinfo1.pdf. [file PSYP-62-e70187-s003.pdf]

ARTEM-IS

Template and Web Application Description

Created on: October 2025
ARTEM-IS version described: ARTEM-IS for ERP v2.1

V2.0
Written by: Anđela Šoškić, Vanja Ković, and Dejan Pajić
Reviewed by: Robert Oostenveld, Nastassja L. Fischer, Yuri G. Pavlov

V2.1
Updated by: Anđela Šoškić
Updates reviewed by: Katarina Stekić

Contents:

- What is ARTEM-IS?
- What does this report contain and who is it for?
- Glossary
- Input and output of the Web App
  - ARTEM-IS for ERP 2.0 Template content overview
    - Mandatory responses vs. percent of completion
  - Output: Understanding ARTEM-IS Reports
    - PDF Report
    - JSON Report
- ARTEM-IS Web App features from the perspective of a user
  - Landing Page
  - Creating and setting up an account
  - Signing in, signing out, and session
  - Menu options after signing in

Adding a new ARTEM-IS Report and editing Reports
Browsing your own Reports and editing Report metadata
Setting up and viewing Report Contributors
Private and public reports
Browsing and viewing public Reports
Downloading an existing Report
Using an existing Report as a template for a new one
Tracking down the origin of a Report
Help/About
How ARTEM-IS Web App works in the background
ARTEM-IS for ERP v2.0 Template Specification
Links to ARTEM-IS Template Specification spreadsheets
ARTEM-IS Web App backend
Template updates, version control and compatibility
Creating new versions of the ARTEM-IS for ERP Template Specification
General guidelines:
Changes to the Presets sheet (response options):
Adding new response options to an existing response set:
Adding a whole new response set
Discontinuing a response option or a whole response set from use
Changes to an existing response option
Rearranging the order of response options
Changes to the ARTEM-IS Spreadsheet (questions list):
Adding new questions (reporting items)
Adding optional questions or making questions optional
Removing questions from the Template
Changes to an existing question
Adding a new subsection and rearranging affiliations of questions with subsections
Rearranging order of questions within a section
Adding an entire new section
Changing order of existing sections
Separating questions in one section into multiple sections or moving a question from one section to another
Editing section description (preamble)
Updating the Web App to a new version of the ARTEM-IS for ERP Template
Keeping track of changes between Template versions
Citing ARTEM-IS
Licence
ARTEM-IS Extensions
Contact
References

What is ARTEM-IS?

ARTEM-IS is a collaborative grass-roots initiative that builds web apps which support EEG researchers in creating detailed human- and machine-readable method summaries using a standardised metadata template. These summaries can be used as supplements, memory aid when writing, or to facilitate metadata extraction. The main purpose is to improve research reporting accuracy, research replicability and reproducibility, as well as to provide better grounds for meta-scientific research in the EEG domain.

You can learn more about the project and read the ARTEM-IS Statement in this paper. If you agree with the Statement, please consider supporting it with your signature by filling in this form. The more up-to-date information on the project can be found in our OSF repository. If you have further questions, or you are interested in joining the team, do get in touch.

What does this report contain and who is it for?

This report is a supplementary document to this paper introducing ARTEM-IS for ERP.

This report aims to provide necessary information to:

a) researchers who want to use the Web Application to generate or read ARTEM-IS-compliant methodology summaries
b) researchers who want to create and use JSON machine-readable methodological summaries
c) new members of the ARTEM-IS Working Group that have not been part of the original development of ideas and who want to understand the logic of the Template Specification and take part in future developments
d) everyone who wishes to use ARTEM-IS as a basis to start similar projects in other domains of neuro- or other fields of science, or to make their own platform for creating ARTEM-IS compliant methodology summaries

As such, this report includes:

- instructions on how to use the ARTEM-IS official Web Application
- instructions on how to read ARTEM-IS Reports
- instructions on how to read an ARTEM-IS Template Specification (i.e., the data sheets that define contents and branching rules of ARTEM-IS Templates)
- guidelines for developing new ARTEM-IS Template Specifications, either to create new versions of ARTEM-IS for ERP, or to create novel Template Specifications

This report does not cover details on how the official ARTEM-IS Web Application specifically works, nor details on the process of version control and updating of the Application with new features. This is because, unlike the ARTEM-IS Template, the Web Application is not publically available at the moment due to practical constraints (see Licence).

Glossary

ARTEM-IS Template: the standardised reporting format for describing methodological properties, i.e., all questions with expected answer formats, branching rules, etc.
ARTEM-IS Template Specification: detailed description of the ARTEM-IS Template contents and branching rules. It consists of two data sheets, ARTEM-IS Spreadsheet and ARTEM-IS Presets Spreadsheet
ARTEM-IS Spreadsheet: spreadsheet describing all questions and their properties (expected answer type, branching rules, sections they belong to, presentation order, etc.)
ARTEM-IS Presets Spreadsheet: spreadsheet describing response options that are offered to multiple-choice questions (list of options, order of presentation, numerical codes, etc.)
ARTEM-IS Web Application (ARTEM-IS Web App): the online web application provided by the ARTEM-IS team and hosted on the INCF server, that allows generating, sharing, storing, and browsing methodological descriptions that are structured according to the ARTEM-IS Template (ARTEM-IS Reports)
ARTEM-IS Form: the online form that is available on the ARTEM-IS Web App that the users fill in to generate methodological descriptions structured according to the ARTEM-IS Template (ARTEM-IS Reports)
ARTEM-IS Report: the methodological description structured according to the ARTEM-IS Template, available for download in PDF or JSON formats through the Web App (ARTEM-IS PDF Report, ARTEM-IS JSON Report)
Question/Reporting Item: one reporting item in the template (appears as one question in the ARTEM-IS Form)
Response set: set of options that are offered to a multiple choice question
Response option: one option within a response set that can be selected when there is a multiple choice question

Input and output of the Web App

Currently, there is one ARTEM-IS **Template**: ARTEM-IS for ERP. The ARTEM-IS **Web App** is currently designed to allow generating, sharing, storing, and browsing methodological descriptions that are structured according to this Template.

The main input of the Web App is the online questionnaire, called **ARTEM-IS Form**, that allows users to generate standardised descriptions in line with the Template. The main outputs of the Web App are **ARTEM-IS Reports**, which can be viewed online or downloaded in JSON, DOCX, or PDF formats (JSON Reports, DOCX Reports, and PDF Reports).

In this section, we will first provide a brief overview of the contents of the ARTEM-IS for ERP Template, and consequently, the online Form in the Web App. Next, we will describe the contents of the PDF and JSON Reports and how these should be interpreted.

ARTEM-IS for ERP 2.0 Template content overview

The current version 2.0 of the ARTEM-IS for ERP Template comprises nine sections designed to assist researchers to report the following aspects of a study: (1) study description, (2) experimental design and sample, (3) hardware, (4) acquisition, (5)

pre-processing, (6) measurement, (7) channel selection for analysis, (8) visualisation, and (9) other. The description of the statistical analysis is not included in this version. Each section contains a set of questions; the answers can be categorical, numerical or short open-ended textual statements. Sections are separated into subsections for easier navigation.

Brief description of each section:

1. Study: This section gathers information about general aspects of the research study: information related to whether a Report documents a planned pipeline or an already applied one; the title of the study; information about the authors; related publications and DOIs; associated available datasets and supplementary materials (e.g., code); supplementary materials; licensing; abstract; keywords; financial support; ethics committee approval; acknowledgements; and citation instructions.

2. Experimental design: This section gathers information about the experimental design and the sample of the study: the number of participants in the study; inclusion and exclusion criteria; information related to the experimental/comparison groups; details on the trials presented and analysed; and software for stimuli presentation.

3. Hardware: This section provides information about the hardware used for data acquisition: information related to the EEG cap/net; electrodes characteristics and placement scheme; information on the amplifier and configuration; description of triggers (e.g., how they were generated and saved); and information related to any additional devices used (e.g., signal boxes, converters, electrode position measurement devices).

4. Acquisition: This section documents information related to the data acquisition process: acquisition software used and version; details on impedances or alternative data acquisition quality measures; references for EEG, EOG and other electrodes, if used, and their alternatives (e.g., Driven Right Leg (DRL)); ground electrode and placement; EOG channels for recording eye movement-dependent voltage and placement; online high pass and low pass filters and notch filters; and acquisition sampling rate.

5. Pre-processing: This section documents pre-processing, i.e., software used for pre-processing, the steps in the pipeline before measurement and statistical analysis of an ERP component: automated preprocessing pipeline; offline filtering; downsampling; re-referencing of EEG, EOG and other channels, if used; artifact removal methods in each elimination step, if there were more than one (rejection of bad trials, data segments, or channels, artifact correction, channel interpolation, multi-step automated approaches); epoching;, baseline correction; other steps. The user can add as many steps as they want in the order they are arranged in the pipeline and at the end of this section, the user is asked whether they would like to additionally self-describe the order of operations in a free-text field in which pre-processing steps were applied.

6. Measurements: This section offers options to describe amplitude and latency measurements of one ERP component, unless the user subjected all time points and channels to statistical analysis (e.g., in a mass univariate analysis). Amplitude properties include: measurement software, waveforms used to measure amplitude, amplitude measure

(peak, mean, window area, etc.) and its parameters, measurement time window, rationale of selecting this exact time window. Similarly, latency properties include: measurement software, waveforms used to measure latency, midpoint latency measure (e.g., local peak, 50% area) - if applied, onset latency measure (e.g., fractional area, fractional peak) - if applied, the appropriate parameters of each latency measure depending on the choices, time window within which the latency was searched for, and the rationale for selecting this exact time window.

7. Channels: This section gathers information about the selection of channels for later statistical analysis. Like in the case of the previous section, the current version of the template supports describing the location for the measurement of one ERP component. There are two main scenarios - all channels are included in statistical analyses (e.g., in a mass univariate approach), or a subset of channels is included in the analyses. Within the second scenario, five options are offered: a priori selection of channels; data-driven selection of channels from the entire scalp; data-driven selection of channels from an a priori selected broader region on the scalp; selection/identification of channels in two data driven steps (e.g., maximal effect within a visually identified broader region); other. Depending on the choice, an appropriate subset of questions follows. In the end of this section, the user is asked to describe whether the channels were entered separately into statistical analysis or if they were aggregated into regions before conducting the statistical analysis.

8. Visualisation: This section provides information related to the visualisation: the type of plot created (e.g., line plot (waveforms), topoplot, ERP grid, butterfly plot); whether any extra pre-processing was performed for visualisation purposes (e.g., smoothing filter, different baseline); description of what the data represents (e.g., single waves, difference waves) and corresponding units (e.g., voltage maps, normalised voltage maps); description of which conditions or difference waves were shown; channels selected for visualisation and the rationale for this decision, beginning and end of the time window visualised and the rationale for selecting this time window.

9. Other: This section contains an option to add additional comments, if there are any.

For a detailed overview of all the items of the checklist, see ARTEM-IS Spreadsheets below.

Mandatory responses vs. percent of completion

ARTEM-IS does not include rules on which items in the Template are mandatory to fill in, though the ARTEM-IS Template Specification has a field that allows for adding this option in the future. This is because the goal of the ARTEM-IS project is not to enforce providing a particular set of information. Our goal, instead, is to help researchers be more detailed and precise in their descriptions, as well as to help readers have an easy and transparent overview of which (and how much) of the necessary information has been provided.

As a result, the ARTEM-IS Web App also does not enforce answering any questions in the online form. Each Report can be saved, downloaded, and publically shared in any stage of the progress of filling out the Form. Instead, the readers of online Reports shown in the Web App and downloaded Reports are provided information on the **percent of completion of a given Report.**

The calculation of this proportion excludes items that are skipped due to the branching logic of the Template. More specifically, in some cases, there are sub-questions that only appear if a specific answer to a previous, “main” question has been selected. In these cases, the proportion calculation includes sub-questions only if they have been revealed to the user after selecting the appropriate response to the main question. The calculation excludes these sub-questions if the sub-questions have not been revealed to a user, either because they have provided a different answer to the main question, or because they have skipped the main question altogether.

Notably, most questions which are part of the Template are described as necessary to provide in a research report by contemporary guidelines for good practice in EEG research (e.g., Keil et al., 2014; Pernet et al., 2020). ARTEM-IS Template clearly separates the few reporting items that are not described as mandatory by the existing guidelines for good practice (e.g., additional filter properties which researchers may want to provide on top of the minimal reporting guidelines). In these cases, the researchers are first asked whether they would like to include this additional information. They are then asked to provide these additional details only if they select “Yes”. If they select “No”, they skip these additional questions. This also means that skipping them does not influence the percent of completion calculated by the ARTEM-IS Web App.

Output: Understanding ARTEM-IS Reports

PDF Report

A PDF Report, shown in Figure 1, is made to be easier to understand by human readers, with full text of both the questions and answer options displayed in the report.

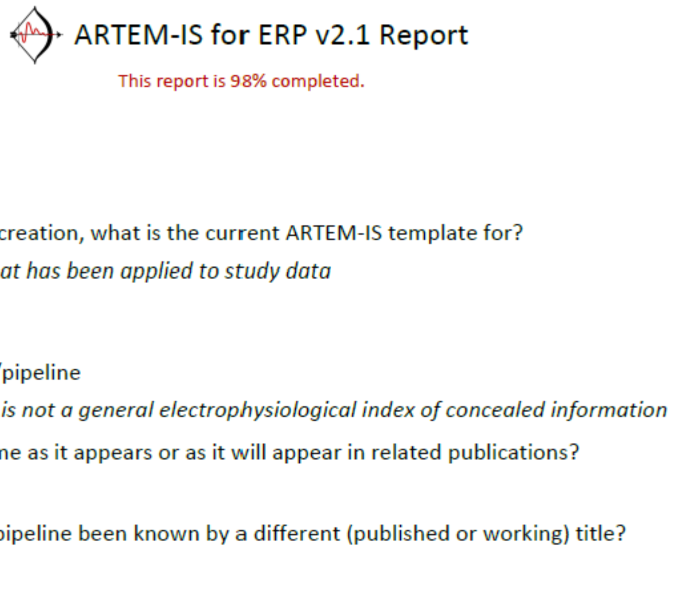

ARTEM-IS for ERP v2.1 Report

This report is 98% completed.

1. Study Description

Study ID

1. At the time of document creation, what is the current ARTEM-IS template for?

Documenting a pipeline that has been applied to study data

Study title

2. Current title of the study/pipeline

Anterior N2 enhancement is not a general electrophysiological index of concealed information

3. Is the current title the same as it appears or as it will appear in related publications?

yes

4. Has the current study or pipeline been known by a different (published or working) title?

no

Figure 1. Example ARTEM-IS for ERP v2.1 Report in PDF format.

This report consists of:

- Information about the percent of items that are completed in the Report.
- A list of all sections, with subsections clearly shown, and with a list of all pre-processing steps showcasing the order of operations in the pre-processing pipeline. Within each section, only the questions that are included according to the previous answers and branching logic are shown. If a question is not included in the branching structure of the Report, the Report reader will be alerted by an appropriate statement (e.g., *"Conditional questions 8 to 11 are not displayed."*). Similarly, if a user filling in the report has omitted to answer a question that is shown to them, the response to this question will state: "*Answer not provided.*"
- At the end of a Report, there is information on (a) Contributors to the Report, (b) licence of the Report, (c) link to the online version of the report, (d) download date, and (e) a note if the Report is created by copying a different Report which has been used as a template to create this Report (a note stating *"**Derivative.** This report is a derivative of [Report Title] by [Contributors]. [link to the original Report that has been used]"* is provided at the end of the Report).
- If needed, the unique `Report ID` of a Report in PDF format can be extracted from the link to the online version of the Report provided at the end of the PDF document.

DOCX Report

The DOCX format offers the same content as the PDF Report, but with a complementary purpose. While PDF offers consistent formatting and wider accessibility, DOCX is more suitable for copying and reusing content.

JSON Report

Unlike PDF Reports that focus on human readability, JSON Reports, shown in Figure 2, are made with machine readability and version compatibility in mind. Structured JSON objects, unlike, for example, CSV files, provide a convenient way to store hierarchically organised, machine-readable information as a set of key/value, i.e., question/response pairs. Additionally, JSON files are exported in the so-called "pretty-print" format which enables users to relatively easily search and browse through the template structure in a text editor. Finally, the JSON format provides a convenient way for users to export and import information and build their own Reports upon the already existing privately shared or public Reports (see Using an existing Report as a template for a new one).

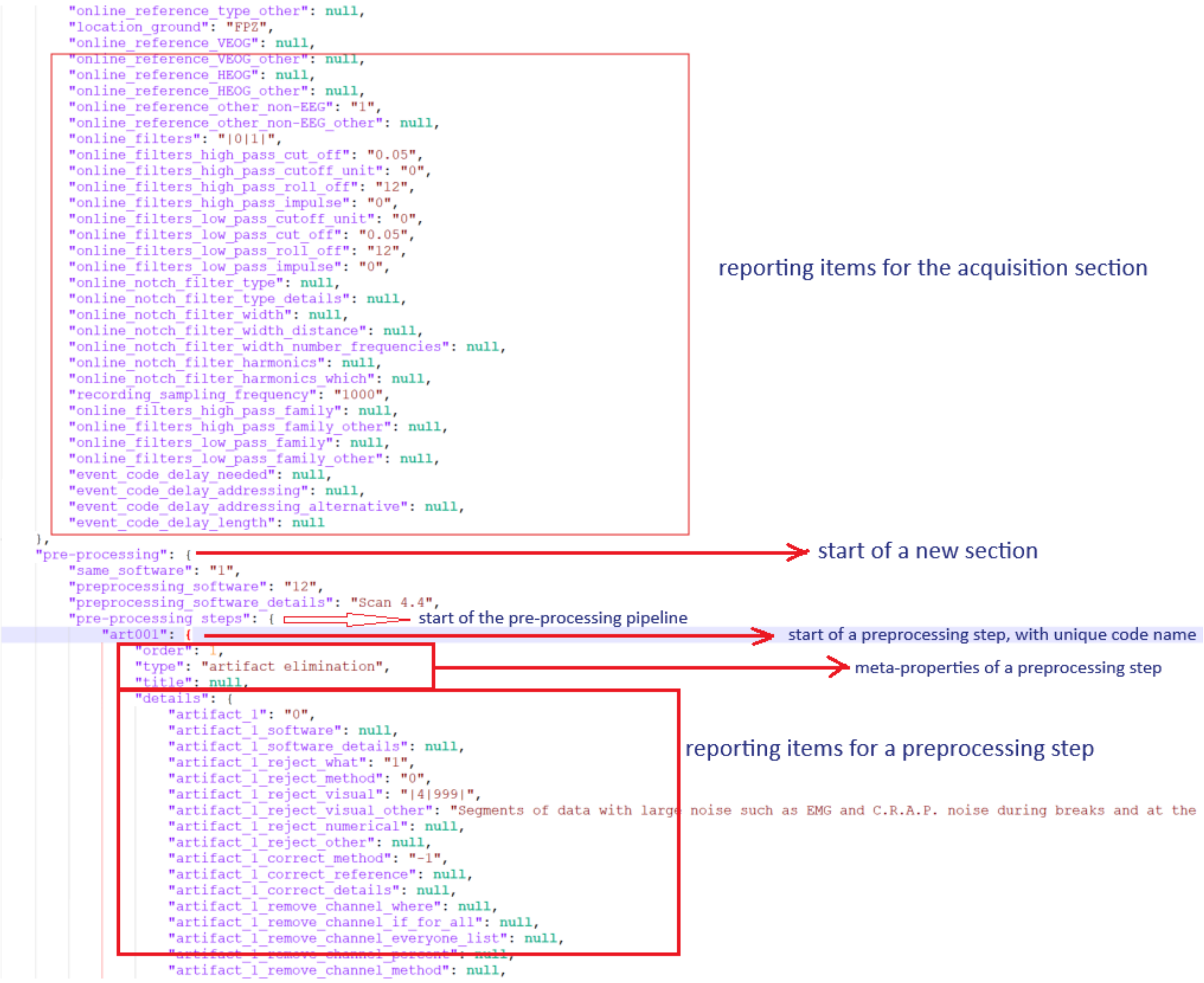

Figure 2. Example ARTEM-IS for ERP v2.1 Report in JSON format.

To achieve these goals, Reports in JSON format feature item codes rather than full question text of the items, as the item codes for the same question remain unchanged between versions of ARTEM-IS, even if minor details change, for example in the case of a slight rewording of a question to make it more clear to readers. Item codes for each question can be found in the ARTEM-IS Template Specification, inside the ARTEM-IS Spreadsheet, in the column `item_pref_label` (see section ARTEM-IS for ERP v2.1 Template Specification for an explanation of the Specification, including the `item_pref_label` column).

Equally, for multiple-choice questions, response codes are given rather than full-text versions of responses. Response codes can be found in the ARTEM-IS Template Specification, inside the ARTEM-IS for ERP v2.1 Presets table, in the column `value`.

Structure of a JSON Report

In its first, `general_information`, section (i.e., JSON object), a Report in the JSON format contains meta-information about the specific Report itself: ARTEM-IS version, the licence under which the report was available (private vs. CC-BY public) when downloaded, together with the date on which the Report has been downloaded, unique ID of the Report (`Report ID`), percent of completion (`Completed`). If a Report has been created by

copying another Report, the unique ID of the original Report that was used as the template will be provided in the field `Source ID`.

This section is followed by separate sections (i.e., JSON objects) that represent each of the major sections in the ARTEM-IS form. Each section contains its reporting items, which are represented using item codes and values or response codes, whichever is applicable. All items without response have `null` values.

Subsections are not represented as such in the JSON Report, with the exception of the preprocessing steps in the Preprocessing section, because this section has the option to add desired preprocessing steps and to have multiple instances of each.

The preprocessing steps are nested inside a JSON object named `pre-processing steps`. Inside this object, there is a separate JSON object for each step. Each object representing the steps has a unique 6-character key (object name) in line with the following convention:

- The first three letters are a code for the type of preprocessing operation: `art` for artifacts, `off` for offline filters, `epo` for epoching, `bas` for baseline correction, `rrf` for rereferencing, `api` for automatic preprocessing pipelines, `dwn` for downsampling, and `oth` for other, self-described operations.
- The latter three characters code multiple instances of the same preprocessing step and code the order in which they were created. For example, the artifact removal step which was created first will be named `art001`, the next one `art002`, and so on. Reordering the steps does not change their object names.

The pre-processing step objects contain step order in the pipeline (`order`), type of the pre-processing step among the offered options (`type`), a self-described title of the pre-processing step (`title`), and the response items related to this preprocessing step nested inside a JSON object called `details`.

ARTEM-IS Web App features from the perspective of a user

In the section that follows, we will assume that the user is accessing the Web App from a PC browser, but the Web App is also designed to be accessible from a mobile device with a small screen, like a mobile phone. .

Landing Page

When first opening the ARTEM-IS Web App, a dynamic, expandable visualisation showing the branching structure of all multiple-choice questions is shown on the landing page, as shown in Figure 3. Each multiple-choice question and sub-question is represented by a node. By clicking on the nodes, the user can expand and hide their branches.

The only other thing is the menu in the top right, which allows registering or signing in to access the Web App contents (creating and browsing Reports), which are available only to registered users, as well as the About/Help page.

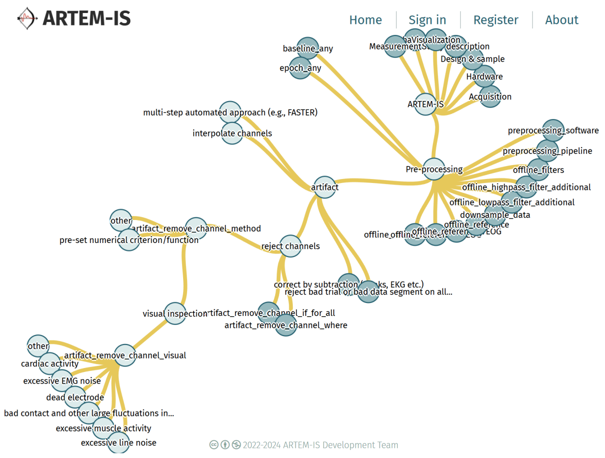

Figure 3. ARTEM-IS Web App landing page

Creating and setting up an account

Before using ARTEM-IS Web App and accessing CC-BY Reports on the platform, researchers need to create an account first by clicking on the option **Register** in the top right corner of the screen. Registration is necessary since the user is responsible for the decision on who would be able to see, download, and/or modify their reports.

To allow additional privacy and easier login for researchers, two options are offered:

1) registration with an email address
2) registration via ORCID account

Registration form is provided in Figure 4. The Registration form is straightforward and requires users to insert personal name(s), email address and a password, while family name(s), affiliation and ORCID are optional.

Researchers who wish to register with ORCID are offered this option at the top of the registration form, and they should follow ORCID instructions upon clicking on the link offering ORCID registration.

Whichever option the researchers select when registering - ORCID-based or email-based, they can add the other one later to allow both, if desired.

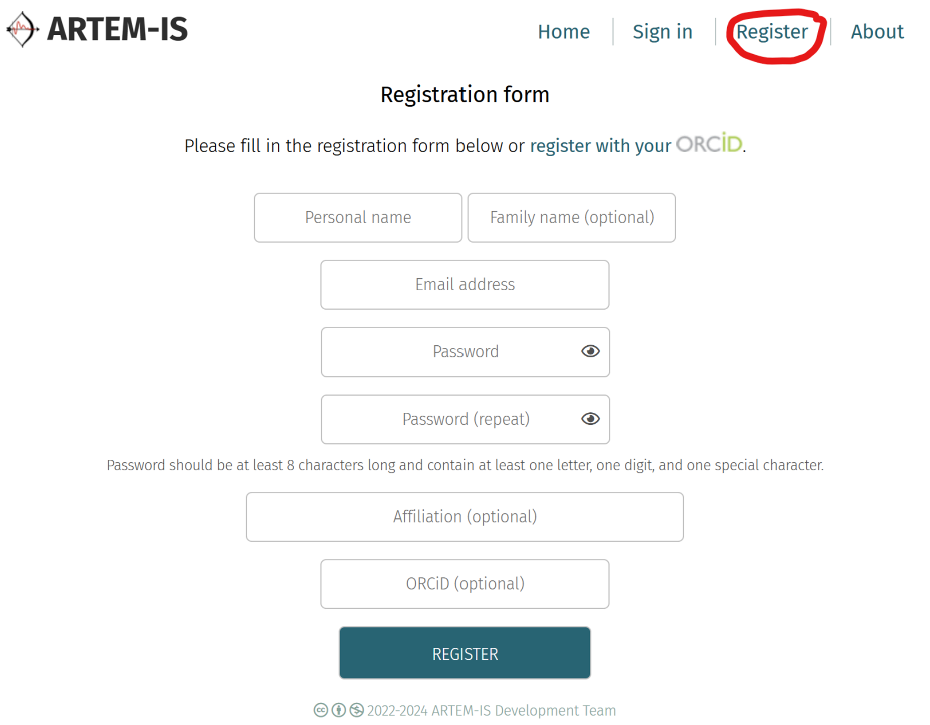

Figure 4. Registration form

The chosen settings can be changed later by visiting **My profile** option in the menu on the top right of the screen.

Signing in, signing out, and session

Once a user account has been created, it can be accessed using the **Sign in** option, which allows users to access their account via email-based login or via ORCID (Figure 5).

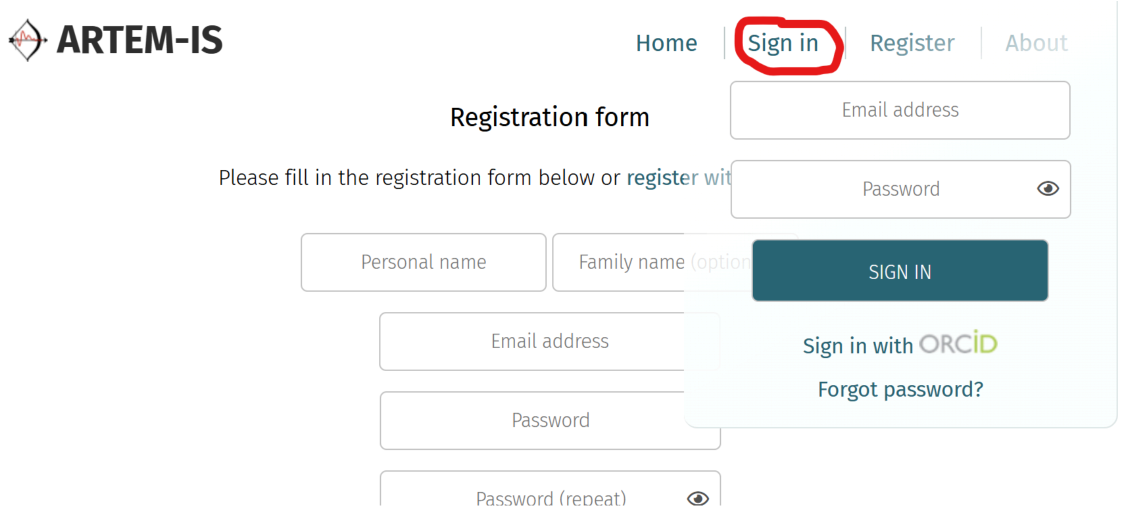

Figure 5. Signing in

Conversely, signing out is done by clicking on the door-and-arrow exit logo at the top right corner of the screen (Figure 6). Additionally, the login session is set to automatically expire after a period of inactivity.

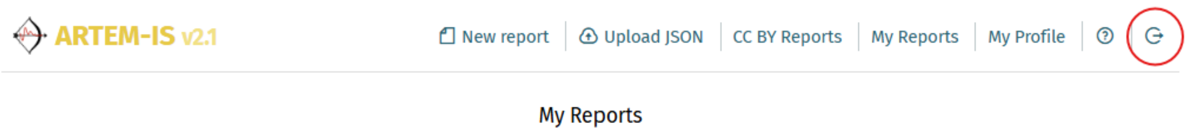

Figure 6. Signing out

Menu options after signing in

Upon signing in, the menu on the top right changes to show the following options (See Figure 7):

- **Create:** create a new Report by starting an empty form
- **Upload JSON**: upload an existing JSON Report to the Web App to create a new online Report with all answers pre-filled/copied from the offline JSON file, and which can be modified further.
- **CC BY Reports**: browse Reports that have been made publically available on the platform
- **My Reports**: browse one's own public and private Reports
- **My Profile:** account settings
- **Help/About**: icon with a question mark, contains information on the ARTEM-IS project and simplified instructions that describe basic options in the Web App
- **Sign out:** icon with the door and arrow

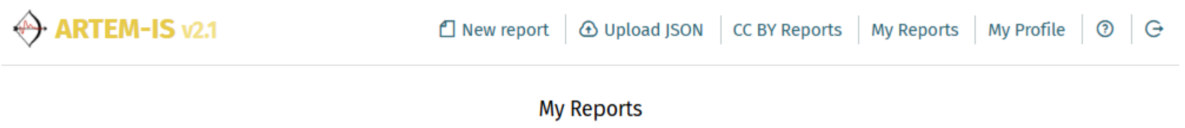

Figure 7. Main menu in the Web App

Adding a new ARTEM-IS Report and editing Reports

A new Report is created from the beginning by clicking on the option **New Report in** the main menu of the Web App. An existing Report can be edited or viewed by clicking on its title either from the **My Reports** or from the **CC-BY Reports** sections.

Any of these actions will open the screen for viewing or editing Reports, which can be seen in Figure 8 (ARTEM-IS Form). Each user can only edit Reports which they have created or to which they have been assigned as a Contributor, and they can view and download Reports that have been marked as publically available, but they will be unable to edit them.

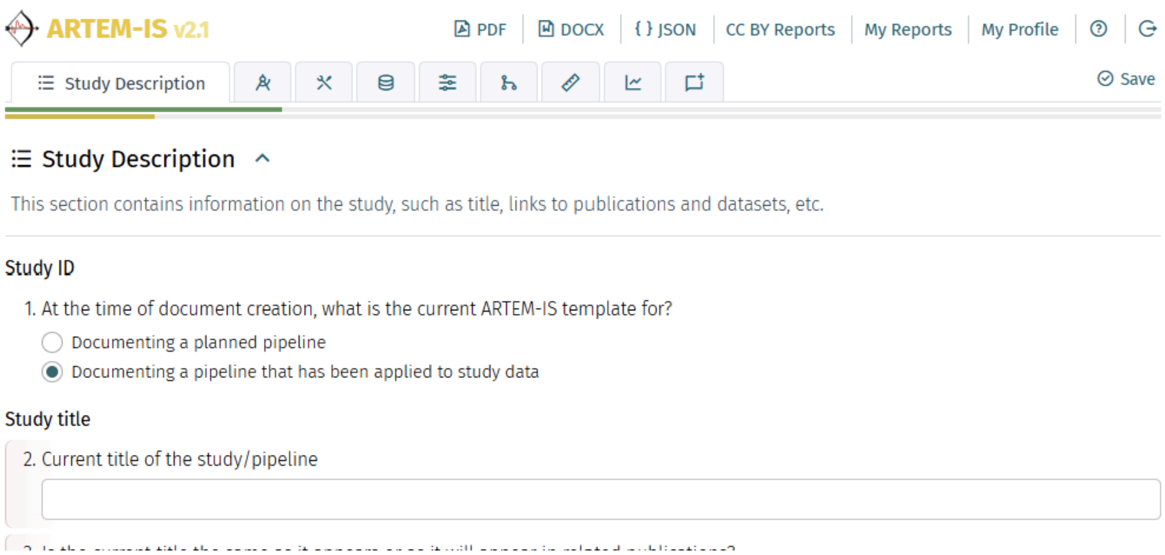

Figure 8. Editing a Report using ARTEM-IS Form

The Report viewing/editing page has a slightly different menu in the top right corner, with the following new options in addition to the already described ones:

- **PDF:** download a PDF version of this Report
- **DOCX**: download a DOCX version of this Report
- **JSON:** download a JSON version of this Report

Below the main menu, there is a bar which allows moving back and forth through different sections of the Report. Each section of the Report is shown in a separate tab, each represented with an icon (hovering over an icon reveals the name of each section).

To the right of all tabs is a **Save** button, which allows saving the progress. To make this easier, changes are saved automatically within 5 seconds as edits are made to a Report and when moving to a different section in the Form. The Save button icon shows a **tick symbol** if all progress has been successfully saved, and a **rotating circle** if uploading is still in progress. Moving on to a new tab or a different menu in the Web App before all the progress has been successfully saved prompts a warning to avoid any information loss.

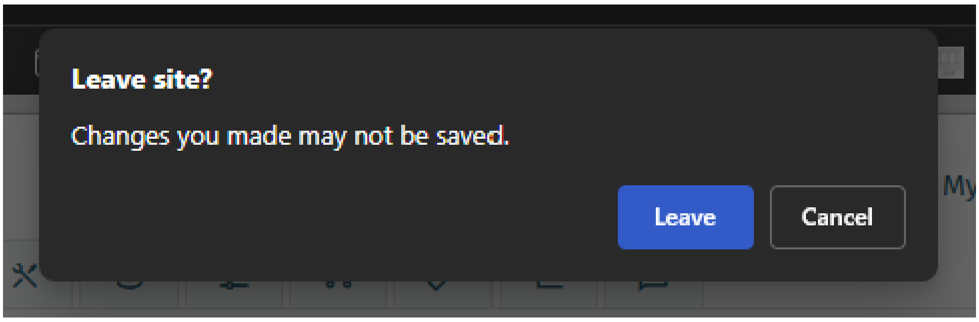

Figure 9. Warning when trying to leave a section before the progress has been saved

Below this menu, there are two **progress bars,** which visualise completion of the Report. The top bar shows the progress in filling in the whole Report, and the lower bar the progress

in filling in the currently open section. By clicking on a progress bar, you will be taken to the first unanswered item in the entire Report or the current section, allowing easier navigation.

After this, the Section title and brief introduction are shown. The introduction can be hidden by clicking on the arrow next to the section title, and it is followed by questions belonging to this section, divided into subsections. Items that are yet to be filled in are clearly marked with red boxes (see question 3 in Figure 8). The user can move between sections either by clicking on the button taking the user to the next section at the end of each page, or by clicking on the tab which they want to open. If a question has an **info logo**, the user can hover over it to get additional instructions on filling in the answer, such as additional instructions on appropriate response formatting or examples of possible answers. It is recommended to have read through these additional instructions when filling in a Report for the first time!

Removing mistakenly added answers. When answering each question, answers can be both added and removed. For multiple-choice questions, a mistakenly selected answer can be removed by clicking on the selected response again to unmark it.

As noted above, there are no mandatory questions to complete a report. Each Report can be saved, downloaded, and publically shared in any stage of the completion progress.

Creating, viewing the pre-processing pipeline

While filling in the questions on most pages is straightforward, describing the Preprocessing sections offers some additional options.

In the Pre-processing steps subsection, the user can:

- add new steps using a **drop-down menu**;
- use **arrows** on the right side to hide and show details for each individual pre-processing step, or use the arrows on the top to control visibility of details for all steps;
- edit details of each step in the same way as in all sections;
- hold and drag the **dots sign** to the left of each step to reorder the steps;
- add unique label for a step by clicking on the **pencil sign** (useful for navigating multiple instances of the same step, which is common with artifact removal) - the new label can be saved by pressing Enter or clicking on the **tick sign**, or the user can press Cancel to close the editing option without saving;
- remove a step using the **bin sign**.

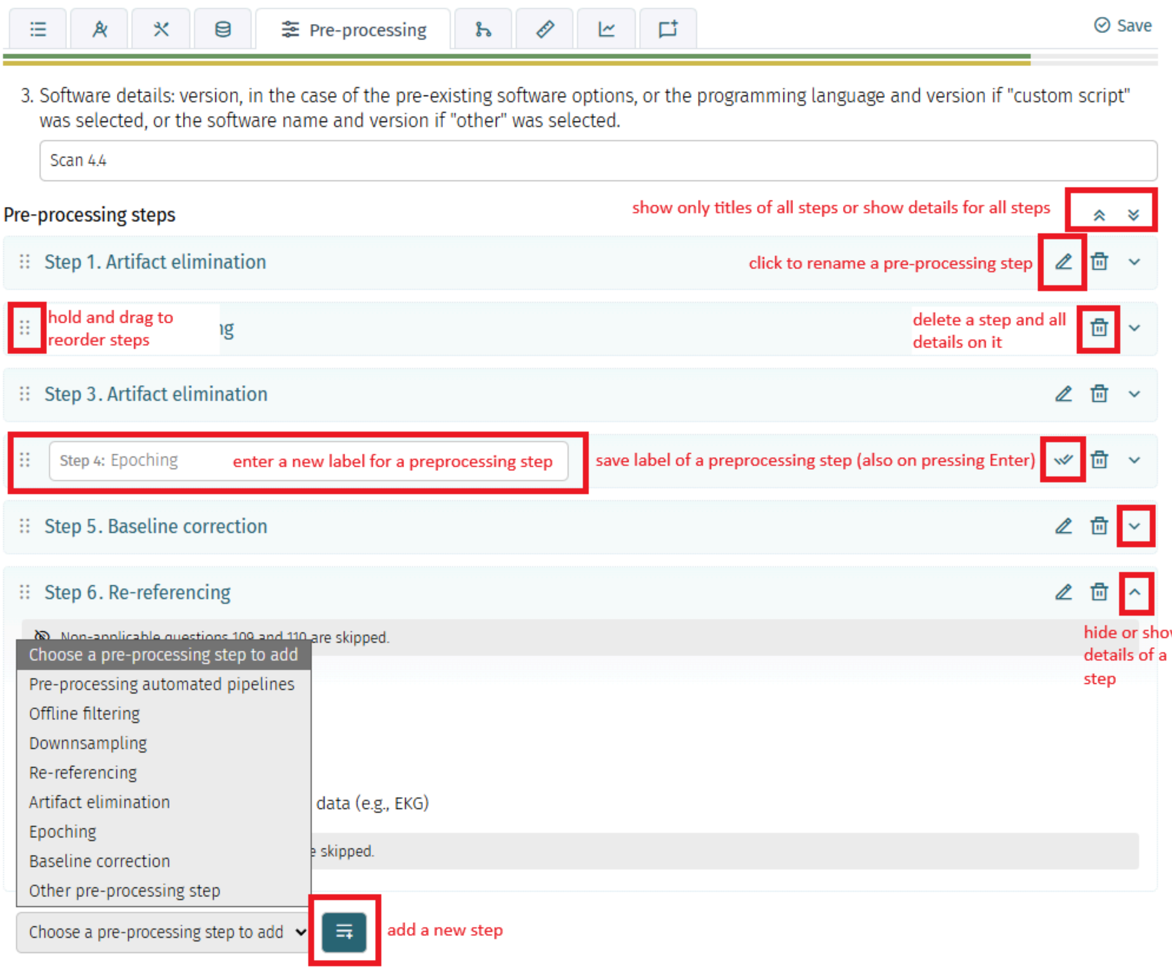

Figure 10. Pre-processing pipeline options

Browsing your own Reports and editing Report metadata

On the **My Reports** page, users can browse their own Reports (see Figure 9). The existing Reports are shown 15 at a time and the full list can be either browsed by navigating through pages of the reports on top of the list or by typing in part of the title in the search bar just above the top of the list.

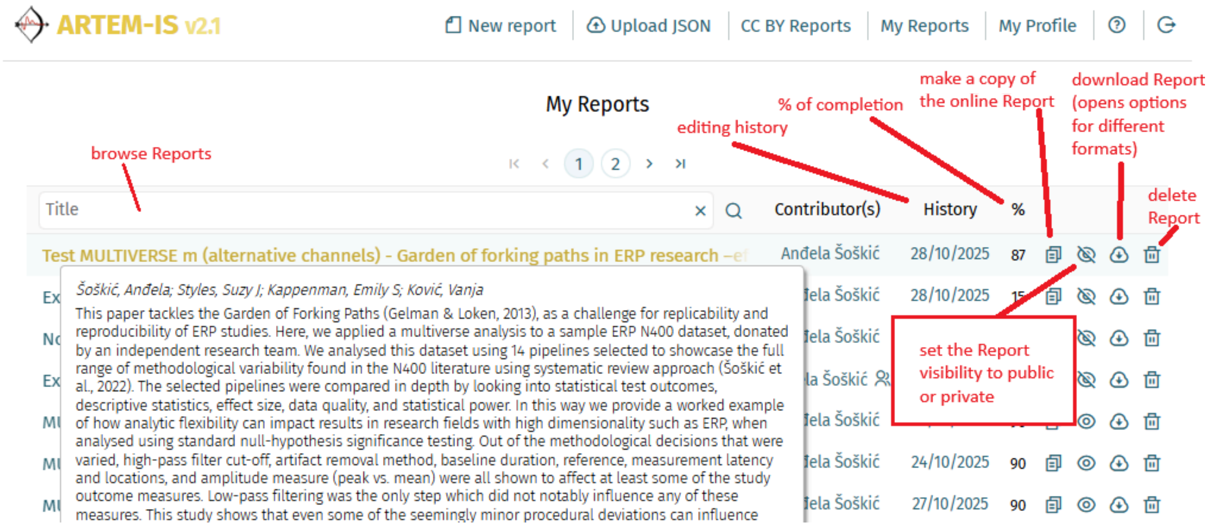

Figure 11. Browsing one's own Reports

Users can (looking at options from left to right):

- open the Report for viewing and editing by clicking on the title
- hover on top of the title of a Report to see the authors and the abstract of the study described in this Report;
- see and edit Contributors to a Report (see appropriate section for more information);
- see last date when the Report was edited in the History column and click on the last date to see a more detailed history of editing;
- see percent of completion of each Report by looking at the "%" column;
- set the Report to be public or private (see appropriate section for more information);
- download PDF, DOCX, or JSON Report by clicking on the download logo and choosing the appropriate option;
- delete a Report by clicking on the trash bin logo.

Setting up and viewing Report Contributors

Report contributors can be viewed from **My Reports** or **CC-BY Reports**, under the **Contributor(s)** column, where the name of the main Contributor (the document creator) is displayed (Figure 10). Hovering over the name reveals additional Contributors if there are any, with a logo of a group of people appearing next to the main Contributor name to draw attention when multiple people are listed.

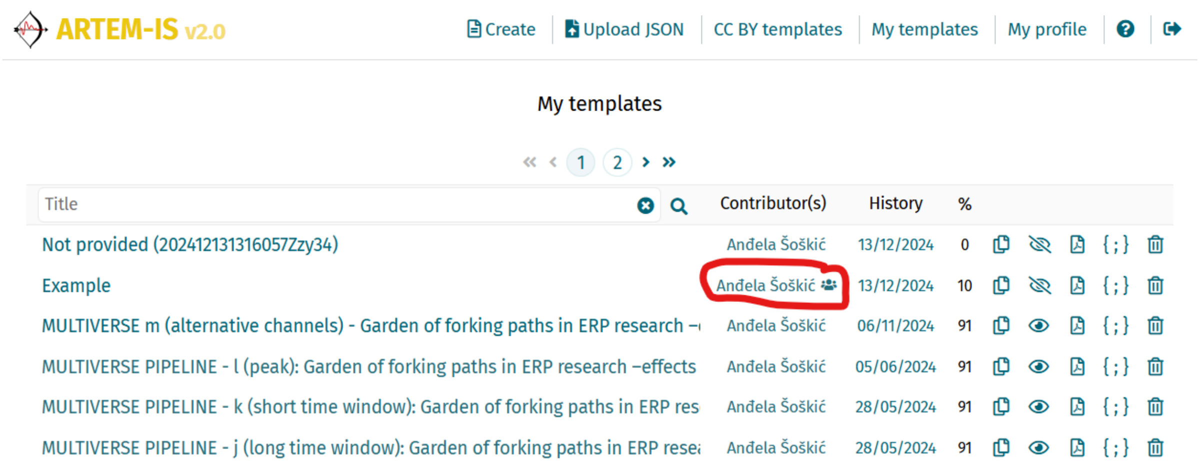

Figure 12. Field for viewing and editing Contributors

Only those already on the Contributor list can modify it—this includes adding, editing, or removing others—but no Contributor can remove themselves from the list. To edit or add Contributors, click on the **Contributor(s)** field (at the main Contributor's name): if you are listed as a contributor, this action opens a screen for editing (see Figure 11), including an option to add more Contributors. Add each new Contributor by typing in the email address tied to their account and clicking on the button to the right. Already assigned Contributors are listed below and you can remove each by clicking on X. If you are not a Contributor, a message stating *"You have no privilege to edit the list of contributors to this report"* will appear.

Information on authors of a study is separate from the information on the Contributors to its ARTEM-IS Report (the list of authors can be found inside the first section of a Report). This allows, for example, researchers to systematically document methods of papers that are published by others for the purposes of systematic reviews and meta-analyses.

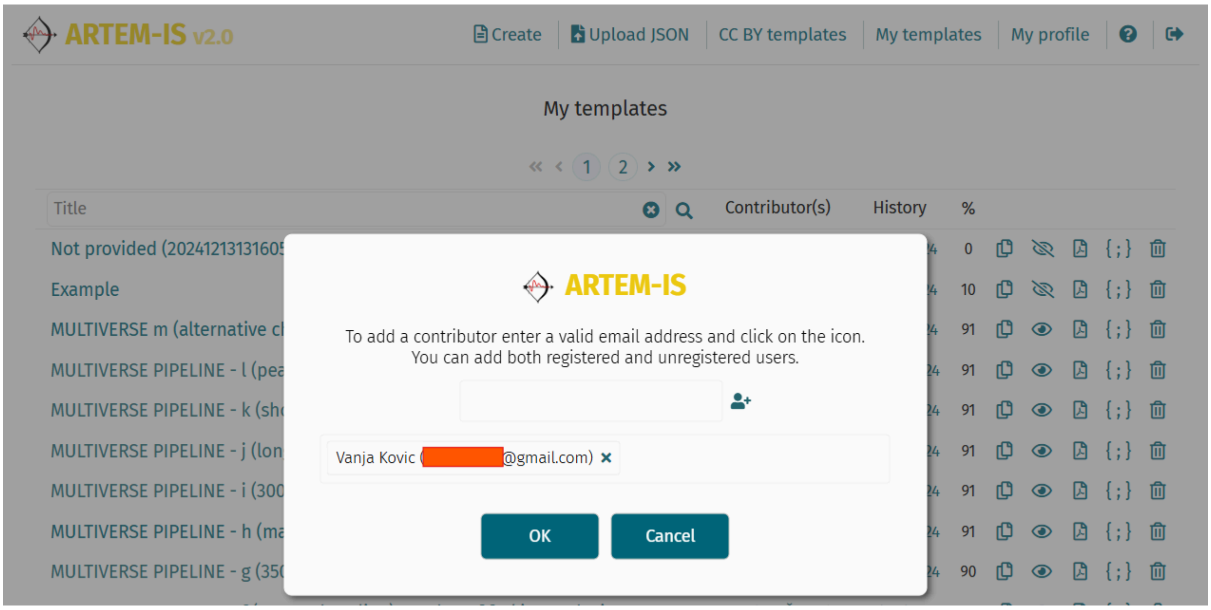

Figure 13. Adding or removing a Contributor

Private and public reports

Reports created by ARTEM-IS Web App users can be saved on the platform in two privacy modalities:

1. **Private Report -** only the users that have been assigned as Contributors to a private Report can see them as well as edit them, in the **My Reports Section**
2. **Public Report with CC-BY 4.0 licence** - if a user opts to make the Report public, the Report is shared with a CC-BY licence and it can be seen on the **CC-BY Reports** page. Only the Contributors to the Report can make edits, and anyone who is a registered user can view, copy, or download public Reports.[1]

Settings for private and public Reports can be viewed on the **My Reports** page, in the column with the eye icon. If the Report is private, the eye icon will be crossed out, while for public (CC-BY) Reports, the eye icon is not crossed out. Clicking on the eye icon toggles the Report's status between **Public** and **Private**. When a user decides to make a private report public, a reminder appears explaining the implications and asking for confirmation: *"This action will make your report public under the CC BY 4.0 licence. Other registered users will be able to see it and make copies, but not to edit it. You can revert this action at any time."* It is important to be cautious when changing the report's status; even if a report was public for

[1] At the moment, ARTEM-IS Web App does not allow sharing Reports under different types of public licenses, though it is a question whether other license types would make sense given the intended uses of the Reports. If this is reconsidered in the future, the ARTEM-IS team will need to consider how this affects permissions for sharing, copying, downloading and uploading Reports, and Web App developers will need to make necessary adjustments to the Web App backend code.

a very short time, there is still a risk that someone may have viewed or downloaded it in the interim.

Browsing and viewing public Reports

Browsing and viewing public reports is similar to **My Reports**, but simplified—there is no **visibility** button or **trash bin** icon. If a user opens a public Report where they are not listed as a **Contributor**, the Report will be frozen for editing. However, the user can still view the Report, download it as a PDF, DOCX, or JSON file, or make a copy of the online Report.

Downloading an existing Report

Reports can be downloaded from:

1. Report Overview (**My Reports** or **CC-BY Reports**) by clicking on the corresponding icon.
2. When a specific report is opened, by selecting **PDF** or **JSON** from the menu in the top-right corner.

The downloaded report can then be shared like any other file through private or public channels or stored in personal archives, for example, to use the static version as an attachment to scientific articles. Alternatively, a link to the online Report hosted on the ARTEM-IS platform can be attached as part of a scientific paper, such as in the Methodology section. However, it is important to note that the online version remains open to further edits, and previous versions cannot be accessed (though the edit history provides information on whether and when the report was modified after the paper's publication).

Using an existing Report as a template for a new one

Let's start with an example—laboratories may want to save *hardware settings* in one place and keep a pre-filled template with the common settings that only needs to be supplemented with offline processing and later steps. Similarly, many laboratories use *standard designs and pipelines*. They can keep records of standard methods to ensure consistency between studies by creating pre-filled reports with these standardized settings, which will also save them significant time when creating ARTEM-IS Reports on these studies. . A third use case is the *replication or continuation of a study*—if a study already has a publicly available ARTEM-IS Report, authors of the replication or follow-up study can copy the existing Report and modify it where deviations occur. Finally, for pre-registered studies, researchers can make a copy of the Report containing the pre-registered plan when creating the Report on the completed study, and easily change only the *deviations between pre-registration and what was eventually done*.

There are two ways to create a copy:

1. *Copy the online report directly* without needing to download and upload it—this is available for both your own reports and public reports. The option to **Copy** (copy icon) is located in the report overview under **My Reports** or **CC-BY Reports**.

2. If a report has been downloaded in JSON format, it can be *uploaded back to the online system.* This can even be done with JSON files of private reports whose online versions are not visible on the ARTEM-IS platform (e.g., when an author downloads and uploads their own Reports or shares them with someone else via email). *Use case scenario:* a lab manager can privately share a half-filled report containing hardware settings with lab members, who can upload it and use the pre-prepared reports to describe all studies conducted with those settings more efficiently.
    - The option to **Upload JSON** is available in the top-right menu of the **My Reports**or **CC-BY Reports** pages.

If a user uploads an older, V2.0, version of the JSON Report, they will be notified that the Report has been converted to V2.1. The user will also be prompted to check and adjust the order of pre-processing steps, an option that was not available in V2.0.

In both cases—whether uploading or copying—the result is a *new online Report* with all answers pre-filled/copied from the original report. The new Report will have a new `Report ID`, distinct from the original report that was copied. The newly created online Report can also have a completely different team of Contributors compared to the original. The main contributor for the new Report is the user who uploaded the offline document or created the copy.

The new Report will be clearly marked as a *derivative* of the original report, with a link to the original source (while ensuring privacy protections for private originals), which will be further explained in the next section.

Tracking down the origin of a Report

As explained in the previous section, if a Report was created by copying another report, it is clearly marked as a derivative of the original report:

1. In the online view within the Web App, a clickable link to the original is displayed just below the progress bar (see Figure 12).

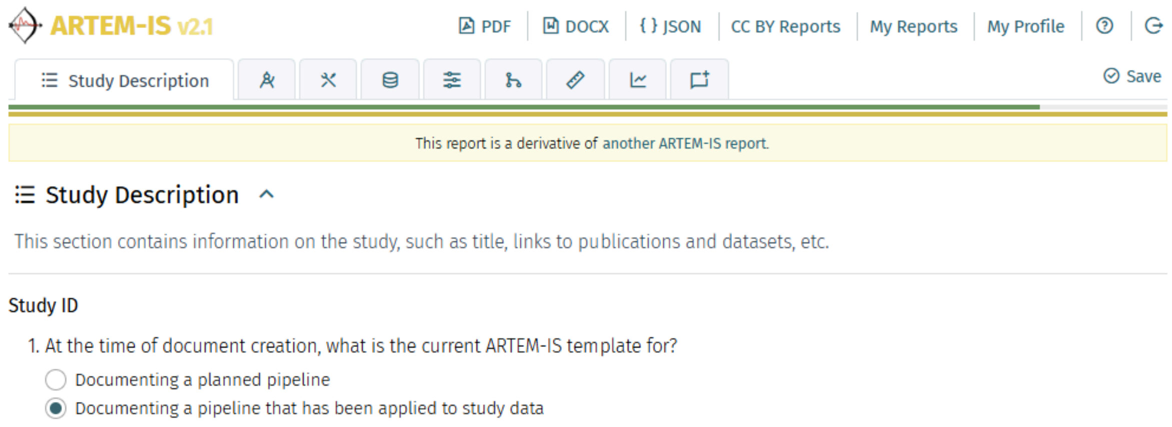

Figure 14. Link to the source Report for derivative Reports in the online From

2. In the PDF and DOCX Reports, there is a clickable link to the original Report at the end of the PDF document, together with a note stating *"**Derivative.** This report is a*

derivative of [Report Title] by [Contributors]. [link to the original Report that has been used]" is provided at the end of the Report).
3. In the JSON Report, the origin is identified by the `Source ID` in the Report metadata section (object) at the beginning of the Report.

Clicking on the link to the original opens the source Report in its online version on the ARTEM-IS platform. If the user has access to the source Report—either as a **Contributor** or because the report is **public**—they will be able to view it. If the source Report itself is a derivative of another Report, the same process can be followed to trace the chain back to the first Report from which the chain originated.

If a source Report has been deleted in the meantime, the user will not be able to access it and will instead receive a notification stating that the report has been deleted. Similarly, if the source Report is private and the user does not have access, they will see a notification indicating that this Report is private.

Help/About

The **Help/About** section offers a brief overview of key user tips, providing a condensed version of the information outlined in this document. It also includes direct links to the project's pages on **OSF** and **GitHub** for further resources. Additionally, the section features an **Update History**, where users can click on any listed update to automatically view a detailed report of changes, as described in the *Keeping Track of Changes* section.

How ARTEM-IS Web App works in the background

An ARTEM-IS Template is defined using two data sheets, described below, which define questions, rules for their dynamic showing and hiding, the appropriate type of answer to each question, and all other properties of the Template. The two spreadsheets jointly constitute an **ARTEM-IS Template Specification**. Defining the Template in the form of data sheets that are edited on the Google Spreadsheets platform by the ARTEM-IS team facilitates collaborative work with a low bar of entry for new contributors in terms of technical skills.

ARTEM-IS Web Application turns these spreadsheets into a dynamic online questionnaire (ARTEM-IS Form), and allows storing and sharing of ARTEM-IS Reports created in the application. There are two main motivations for creating the Web Application. The first motivation is to enable easy creation of ARTEM-IS Reports. Methodology summaries organised in line with the ARTEM-IS Template can be created manually without the support of the Web Application, but it would be a much more tedious process. The second motivation is to facilitate collaboration on ARTEM-IS Reports and exchange of information between researchers creating and using the Reports.

In the section that follows, the Specification will be described first, followed by the Web Application.

ARTEM-IS for ERP v2.1 Template Specification

The first spreadsheet, called simply **ARTEM-IS Spreadsheet**, contains all questions that can be found in a Form/Template and specifies their properties. For ARTEM-IS for ERP v2.1, this table is called ***ARTEM-IS for ERP 2.1 Spreadsheet***. The table contains separate tabs for each section of the Template (visible as a separate tab in the online Form), to make the spreadsheet easier to navigate (see Table 1):

Table 1. Tabs in an ARTEM-IS for ERP v2.1 Spreadsheet.

Tab name in the Spreadsheet	Corresponding tab in the Web App
`study_id`	Study Description
`experimental_design_sample`	Design and Sample
`hardware`	Hardware
`acquisition`	Acquisition
`preprocessing`	Pre-processing
`measurements`	Measurements
`channels`	Channels
`visualization`	Visualization
`other`	Other

Within each tab, the rows represent questions that are found within the given section of the Template. Columns in the tables contain the following properties of these sections (see Table 2).

Table 2. Columns within each tab in the ARTEM-IS for ERP v2.1 Spreadsheet.

Column name in the Spreadsheet	What does the column define	Additional info about the column with advice for designing future templates
`sub_section`	Subsection within the Form/Template section to which a given question belongs. Visible in online and PDF Reports.	All questions that belong to the same section have the same value in this field.<br><br>Subsections are defined here, there is no separately defined pre-set list of subsections. Simply, the questions that are consecutive and have the same `sub_section` value are shown within one subsection of the Web App. The title of a subsection in the online form is this value of the `sub_section` column. The purpose of the subsections is simply to help

		the user navigate the list of questions.
`preamble`	Contents of the brief description on top of each tab in the Web App that introduce a given section (e.g., “This section contains information on the study, such as title, links to publications and datasets, etc.”)	The description of a section should be placed in this column and in the same row as the first item in the section. The remaining rows in each tab can and should be left blank, as no information is read from them. This was just a convenient location to preserve section descriptions within the same spreadsheet as the rest of the Template contents.
`question`	Item question, as it appears on the screen of the Web App and in the PDF report	
`details`	Additional instructions for the user on how to answer a given question that show up when a user hovers over the question mark next to a question	It may contain instructions on the way to format the answer, examples of correctly filled answers, and other information.
`field_type`	Type of question (e.g., multiple-choice).	Options to put in here:<br>1. `text` - open-ended question with an expected short answer<br>2. `textarea` - open-ended question with an expected longer answer (more than one sentence or a list of a couple of items)<br>3. `radio` - a multiple-choice question shown as a list with radio buttons, with one possible answer<br>4. `radio_multiple` - a multiple-choice question shown as a list with checkboxes, where multiple options can be selected simultaneously<br>5. `select` - a multiple-choice question shown as a drop-down list where only one option can be selected<br>6. `select_multiple` - a multiple-choice question shown as a drop-down list where only multiple options can be selected

		simultaneously<br>7. `integer` - a number without decimal fractions<br>8. `float` - a number with decimal fractions<br><br>When creating ARTEM-IS for ERP v. 2.0, we tried to avoid open-ended questions as much as possible, especially with longer answers because this opens space for ambiguous descriptions and it is more difficult for meta analyses. However, this is an ambitious goal, so there are still some sections that could benefit from being broken down into multiple shorter questions in future iterations of ARTEM-IS for ERP.
`choices`	Defines which choices will be offered in multiple-choice questions (`radio`, `radio_multiple`, `select`, `select_multiple` types).	Sets of response options for multiple questions are defined in a separate spreadsheet called *Presets* (see below). This spreadsheet contains a `type` column which essentially defines variable names for each set of options.<br><br>The `choices` column contains the variable name (i.e., `type`) of a set of responses that should be offered to a given question. In other words, for each multiple-choice question, the Web App will offer all response options whose `type` value in the *Presets* spreadsheet corresponds to the `choices` value in the main spreadsheet.
`item_pref_label`	Variable name and simultaneously a unique ID of each question<br><br>This is the title for each question in the JSON report	This value is unique for each question, because this is the variable name for each question property in the backend of the Web App.<br><br>If a `question` text is rephrased for clarification purposes, but it should still be treated as the same question, it is `item_pref_label` that allows identifying that this is still the same question (see Version control for more details).<br><br>There are currently no discontinued

		questions from previous Template versions, but it is important that `item_pref_label` names of old, discontinued questions are not reused in the future to prevent issues with version compatibility between different versions of an ARTEM-IS template (see Version control for more info).
`visibility`	Logical expression that determines whether the item should be displayed or not.	The question is visible if its value is 1, and not visible to a user if its value is 0. If the question should always be visible, the value of this field should be simply 1. If the question should be visible only under certain conditions (depending on responses to other questions), they should be entered here in the form of logical statements written in JavaScript programming language.<br><br>In ARTEM-IS v2.1 there are no conditional questions that refer to responses in a different tab to simplify the backend of the Web App.
`item_order`	order in which a given item appears within a section, as shown on the Web App screen and in PDF Reports	The order number of the first question is 1 for each sheet of the Google spreadsheet / each tab of the Web App / each section of the PDF report.<br><br>The question order increases while going down a tab in the spreadsheet (i.e., the order of the questions in the Spreadsheet is the same as the order in which the questions are shown in the Web App).<br><br>There are currently no discontinued questions because this is the first stable version of ARTEM-IS for ERP. In the future, any discontinued questions should not have any value in the `item_order` field. Instead, they should be skipped and counting the order should continue from the first included question in line.<br><br>*Important!* The value in the spreadsheets here is filled in automatically using a formula that

		gives the order 1 to the first question in a tab, and then the order increases incrementally while skipping questions whose `include` value is 0.
`include`	whether a question should be included in the Template/Form or not	Value should be 1 for included questions and 0 for discontinued questions that are no longer in use.<br><br>The purpose of the column is to allow discontinuing use of questions in the future updates of ARTEM-IS Templates while preserving information on which questions existed in earlier versions of an ARTEM-IS Template and what their unique ID used to be. The latter is important for version control (see later).
`mandatory`	This defines whether it is mandatory to answer a question in order to complete the Form.	**This column is currently not in use, and it is ignored by the Web App.** Currently, there are no mandatory questions (see ARTEM-IS Web App features).<br><br>Value is 1 for all questions at the moment.
`activity_pref_label`	This is the variable name of the section that a question belongs to in the backend of the Web App. In ARTEM-IS JSON reports, `activity_pref_label` acts as the name of each section.	All questions that belong to the same section have the same value in this field. At the same time, because sections of the Web App are separated into different Spreadsheet tabs for easier navigation, all questions within the same Spreadsheet tab have the same `activity_pref_label`.<br><br>Because this is a variable name in the backend of the Web App, it is also crucially important that all sections have unique values of `activity_pref_label`.
`activity_order`	Place of the section that a question belongs to within the order of all sections.	All questions in the first section have value 1 (as opposed to 0 as an alternative possibility).<br><br>Because sections of the Web App

	For pre-processing steps, which can be added, reordered, and removed, this column also contains a unique letter code for each pre-processing step (e.g., 5a, 5b).	are separated into different Spreadsheet tabs for easier navigation, all questions within the same Spreadsheet tab have the same `activity_order`, with the exception of the Preprocessing section, where there are also letter codes for the preprocessing steps.
`unit`	Physical unit in which a numerical value of a physical measure should be entered (e.g., Hz, ms, dB/octave).	This value is shown next to the field where an answer is filled in.
`BIDS_file, BIDS_key, BIDS_key_for_unit, BIDS_status`	Fields that help keep track of correspondences between ARTEM-IS and BIDS to help maintain consistency of terminology and allow potential future integrations, to allow, for example, import of information directly from BIDS-compliant datasets	This information from the Spreadsheet is currently not used in the Web App, it is here for potential future development.
`Change_log`	History of changes to a given question.	Each change is preserved in the format of date, name of the person who made the change, and then a brief description of the change.<br><br>This log has been introduced while preparing the upgrade to ARTEM-IS for ERP v2.0, so only changes starting from the update from v1.0 to v2.0 are logged.

In addition to this spreadsheet, there is a spreadsheet that defines answers that should be offered to multiple-choice questions. These are defined in a separate spreadsheet because there are multiple instances when different questions have the same answer options (an obvious example are yes-or-no questions), and in these cases only one unique response set is used by multiple different questions. For ARTEM-IS for ERP v2.1 this table is called ***ARTEM-IS for ERP 2.1 Presets***.[2]

[2] Why call it Presets? The name is borrowed from eCOBIDAS spreadsheet format, which we used when creating ARTEM-IS v1.0 Spreadsheet, and which we largely based ARTEM-IS v2.0 Spreadsheet and its later updates on. In eCOBIDAS, most response sets are defined within the main spreadsheet together with the questions, while some response sets are defined in their own spreadsheets, usually those that are used often by different questions or those that include a lot of options. In eCOBIDAS terminology, the response set variables defined by these separate spreadsheets are called Preset Variables. We moved away from this format for ARTEM-IS v2.0 and opted to have one spreadsheet containing all response sets to allow better version control and easier oversight, but tried to keep as much consistency to foster exchange and collaboration between ARTEM-IS and eCOBIDAS teams.

This spreadsheet has one tab, in which all response sets are defined. Each unique response set for a multiple-choice question is represented by as many neighbouring rows as there are choices (options), and the response set name (column `type`) defines which answer options belong to the same response set.

> For example, the response set for the yes-or-no question is called “boolean” and it has two options, “yes” and “no”. In the ARTEM-IS for ERP 2.0 Presets spreadsheet, it is represented by two rows, one for each option. Both answer options have the same response set name (`type` column) to indicate that they belong to the same response set (see Figure 13).

1	id	type	name	value	newvalue	sort	active	Last id: 1391
65	1064	boolean	no	0	0	0	1	
66	1065	boolean	yes	1	1	1	1	

Figure 15. Representation of response sets for multiple-choice questions in an ARTEM-IS Presets spreadsheet.

Therefore, each row is one choice within a response set. Notably, different response sets may offer the same answer option (for example, many different questions allow the user to select “other”). In such cases, each instance of the choice that appears multiple times is treated as a separate unique response option, and it has its own row within the group of rows representing a given response set.

Columns in the ARTEM-IS for ERP 2.0 Presets table are described in Table 3.

Table 3. ARTEM-IS for ERP 2.0 Presets columns

**Column name in the Presets spreadsheet**	**What does the column define**	**Additional info about the column with advice for designing future Templates**
`id`	Unique ID of each response option	Different response sets may offer the same answer option (for example, many different questions allow the user to select “other”). In such cases, each instance of the choice that appears multiple times is treated as a separate unique response option, and will have its own `id`.<br><br>The codes have four digits. The first item has ID 1001, and the others take other values, currently sorted in an ascending order and incrementing by 1 in each new row. However, this will not necessarily be the case in the

		later versions of ARTEM-IS: new options that will potentially be added to response sets will take unused larger values and they should still be placed in the middle of the sheet, next to the other options in the same response set.<br><br>When updating to new versions: if a response option is being simply rephrased for clarification purposes, but it should still be treated as the same response option, it should retain the same `id,` which allows identifying that this is still the same answer (see Version control for more details).<br><br>On the other hand, it is important that `id` values of old, discontinued options are not reused in the future for new options to prevent issues with version compatibility between different versions of an ARTEM-IS template (see Version control for more info). There are currently no discontinued options.<br><br>If a new response option is added in the future versions of ARTEM-IS, the correct way to do it is to give it a new, previously unused ID, which should be the smallest available integer value (see Version Control for more info).
`type`	Unique name of the response set to which this answer option belongs.	All answer options that belong to the same response set have the same value in this field.<br><br>Because `type` is used to group response sets, it is crucially important that all response sets have unique values.<br><br>To avoid any version control issues, new response sets created in the future versions of ARTEM-IS should not reuse `type` values of discontinued response sets.
`name`	text of the answer option, as it appears on the	

	screen of the Web App and on the PDF report (e.g., “yes”, “no”)	
`value`	ID code of a response option *within its response set*.<br><br>These are shown as response values in JSON Reports.	Usually, this will be 0 for the first option within the response set to appear on the screen, and then 1, 2… etc. for the following response options, in an ascending order.<br><br>However, this does not need to be the case. Currently, two response options have special codes: code **999** is used to indicate response “other”, and **998** is used to indicate custom solutions.<br><br>Designers of the future versions of ARTEM-IS may want to use this functionality in other ways, too, for example by designating ranges of `value` options (e.g., 101-199, 201-299) to describe subsets of options, but they should not use values larger than 997 to keep 998 and 999 as the largest codes in use.
`sort`	order in which options appear on the screen within a response set	The first item to be presented has sort value 0, the next one 1, etc.<br><br>`Sort` value does not necessarily match `value`, for example if there are items with special `value` codes, such as 999 (code for “other”), or if some options are discontinued in later versions of ARTEM-IS (no longer `active`).<br><br>In addition, sort allows designers of the future versions of ARTEM-IS to sort response options any way they want on the screen without changing unique response codes (`value`). (see Version Control for more information)
`active`	Takes value 1 if the response option is currently in use, and value 0 if the response option has been discontinued.	Currently, all options are active and they have value 1, but this should be used to discontinue options in the future, rather than deleting the entire row altogether. This way, information on `value` and `id` codes that were once utilised can be preserved for

		version control purposes (see Version Control).
`change_log`	History of changes to a given item.	Each change is preserved in the format of date, name of the person who made the change, and then a brief description of the change.<br><br>The column logs only changes starting from ARTEM-IS for ERP V2.0.<br>.
`Last ID`	There are no values in this column, this is just a counter that shows the largest ID value.	This is meant to help the person adding new response options easily determine which is the smallest available (unused) ID value.

Links to ARTEM-IS Template Specification spreadsheets

Table 4. Links to ARTEM-IS Template Specification Spreadsheets

Which form	Spreadsheet	Version	Link
ARTEM-IS for ERP	Question settings	work-in-progress, Google Spreadsheet: v2.1	https://docs.google.com/spreadsheets/u/0/d/10Mn0MvrEMRqSv7JYWJkwFBWAprww2SL9ozaIAAqH0Qs/edit
		static version of v2.0	https://osf.io/3ga5e
		static version of v2.1	https://osf.io/vyuqm
	Question settings *with response options*	static version of v1.0	https://osf.io/9dweq
	Response options for multiple-choice questions	work-in-progress, Google Spreadsheet: v2.1	https://docs.google.com/spreadsheets/u/0/d/1cXUaA6CpuXN_w9OBVMz7DXH0smpqUycI8vUhS9fYFms/edit
		static version of v2.0	https://osf.io/wj94q
		static version of v2.1	https://osf.io/8nuae

ARTEM-IS Web App backend

As mentioned already, the Web App code is not publically available at present (see Licence), so this section provides only a brief overview of how the ARTEM-IS Web App works.

The ARTEM-IS Web Application is a custom solution, created in PHP and JavaScript, using MySQL as a database backend.[3]

At present, the Web Application supports managing only one version of one ARTEM-IS Template. This has two notable consequences. Firstly, if there is an update to the ARTEM-IS for ERP Template, all the Reports in the Web App will be migrated to the new version of the Template. However, it is an important goal of the ARTEM-IS project to keep versions of the same Template mutually compatible as much as possible. Secondly, if ARTEM-IS extends to additional subfields of EEG, this will require additional Web Application development.

The Web Application has an option to load Google Spreadsheets containing ARTEM-IS Specification, and it uses the Specification to define the contents of the online Form, its configuration (e.g., visibility of sub-questions), as well as a backend MySQL database table which stores Reports and their metadata (e.g., `Report ID`, list of Contributors).

Loading Google spreadsheets into the Web App to redefine the ARTEM-IS for ERP Template is done only when updating the Template, not in real time, and it is done by designated ARTEM-IS Team members.

Administrator rights and access to ARTEM-IS data in the Web App backend. There are two important questions for the ARTEM-IS project when it comes to administrator roles: (1) ensuring privacy of information on the platform; (2) ensuring the sustainability of the Web App, i.e., that all backend data can be accessed by the ARTEM-IS team in the case of need.

Currently, there is one designated ARTEM-IS WG member, who is in charge or handling the Web App backend (ARTEM-IS Web App Administrator), and who has full access to it.

On top of this, there is currently one additional ARTEM-IS team member with update rights, who can update ARTEM-IS Template to a new version (see Version Control). ARTEM-IS Web App Administrator can grant update rights to additional WG members.

In addition, since ARTEM-IS is an International Neuroinformatics Coordinating Facility Working Group and the Web App is hosted on their platform, members of the INCF IT Team who maintain the main INCF website have control over the subdomain where ARTEM-IS is hosted. They do not maintain the Web Application or have access to its backend. However, as main administrators of the INCF website, if there is need, they could obtain access to the

[3] There are existing, open-source, off-the-shelf software solutions for questionnaire design, and some of them would make ARTEM-IS more compatible with the similar attempts in the neuroimaging field, in the first place eCOBIDAS, with which ARTEM-IS shares the goal to create reporting tools, and which very generously supported ARTEM-IS team in designing the initial versions of the Web App. Unfortunately, none of these solutions are flexible enough to offer all features that were needed for the ARTEM-IS Web Application. The niche needs of the ARTEM-IS project compared to a typical questionnaire forced us to develop a custom solution, while trying to keep the ARTEM-IS Template Specification as similar as possible to eCOBIDAS to facilitate collaboration.

Web Application backend and grant administrator rights to additional ARTEM-IS Working Group members.

Template updates, version control and compatibility[4]

This section concerns rules for updating an ARTEM-IS Template Specification to a new version. These guidelines have been set in place when creating ARTEM-IS v2.0 to ensure compatibility between versions and clear relations between different versions of the same template.

Creating new versions of the ARTEM-IS for ERP Template Specification

Guidelines for updating the Template Specification to a new version are provided in this section. Before you read them, make sure to read the section on the **ARTEM-IS for ERP V2.1 Template Specification** carefully.

General guidelines:

1. Prior to making any changes, ensure that a static version of the current official Template Specification has been created and deposited to OSF and GitHub for record keeping.
2. Prior to making any changes, make sure that you are familiar with the general ARTEM-IS Design Guidelines.
    a. The Template Specification is aligned with the Design Guidelines as much as possible at the time of implementing v2.1, but not fully, since creating a list of reporting items that are as brief as possible, clear and fully unambiguous, machine readable, easy to fill in, etc., is an ambitious goal. The current version of the Template has parts that would benefit from being further broken down into smaller reporting items and could generally be improved to comply with the ARTEM-IS Design Guidelines.
3. Use the `change_log` column in the spreadsheet to describe the changes that you have made by specifying the date of the change, your name and a brief description.

---

[4] This does not apply to the update from ARTEM-IS for ERP v1.0 to v2.0, which is a special case. As explained earlier in this text, v1.0 was a pilot version of ARTEM-IS created to gather community feedback on the general concept, and it did not have version control features described here. Version control protocol was established in v2.0, and the Template Specification was restructured to allow easier version control.

ARTEM-IS for ERP v1.0 has not been in practical use in any studies or publications, and the handful of ARTEM-IS v1.0 Reports that did exist on the platform at the time of the update from v1.0 to v2.0 have all been updated to v2.0 in a one-off update that ensured that all values are correctly transferred to v2.0 (content-wise, there has been no loss of information). Therefore, there are no ARTEM-IS for ERP v1.0 Reports in practical use.

The full list of changes between v1.0 and v2.0 can be found on https://artemis.incf.org/help

Do not erase previously existing content in this field, but rather add your change to the end, after the previously documented changes.

   a. Changes related to the `preamble` text or the entire section (for example, changes to section activity_order) should be logged only in the first row of the section.

4. **Do not** make changes to values that act as variable names (`type` in Presets and `item_pref_label` or `activity_pref_label` in the ARTEM-IS Spreadsheet) or unique identifiers (`id` and `value` in Presets). Values are unique, cannot change over time and cannot be reused.
   a. Also, **do not** reuse these values elsewhere.
5. After implementing an update, make sure that all changes are implemented correctly (for example, minor typos in the `visibility` field can prevent the question from displaying in correct situations)

Changes to the Presets sheet (response options):

Adding new response options to an existing response set:

- Adding new response options does not pose challenges to compatibility as long as the remaining options remain unchanged
- Steps to add a new response option:
   a. Add a new row to the Presets sheet next to the other response options in the same set, in such a way that response options are sorted in the same order as they will appear on the screen.
   b. Find the currently largest existing response option ID across all response options in the whole table (you can see it in the title row of the `Last id` column).
   c. The unique ID of the newly added response option should be the next available number. For example, if the largest ID at the moment is 1903, use 1904 as the `id` for the new response option).
   d. `type` value for the new response option should be identical as the `type` value of the other response options in the same response set
   e. Input the text of the response option as it will appear in the ARTEM-IS online Form and in the PDF Report into the field `name`
      i. If you are adding an option that is essentially “other”, try to formulate the whole response set so that the response text (`name`) can be literally just “other” (not for example “something else” or “other solution”) to maintain consistency across different response sets.
   f. In the `value` column, choose the unique numerical code for this new item, which will appear in JSON Reports and the backend of the Web Application.
      i. **Do not use** any of the existing values in the same response set, even if an option in the response set has been discontinued (its `active` value is 0). Values must remain unique, cannot change over time and must remain unique.
      ii. In many cases, the new unique numerical code will be just the smallest available integer (e.g., if 0, 1, 2, 3 are already taken, you will want to use 4 as the numerical code for the new value). However, you

may also want to designate specific code ranges to the new values, for example if the response set has code ranges with different meanings (e.g., codes 101-199 for one category of responses, and codes 202-299 for a different category of responses).

iii. There are two universal codes that should be the same across all response options: If the new response option is “Other”, its `value` should be 999. If the new response option refers to a custom-made solution, such as custom processing scripts or electrode layouts, its `value` should be 998. You should use these codes for Other and Custom answer options, and **not use** them for anything else.

iv. If you have added an option to respond “Other”, make sure to also add an open-ended text sub-question to the ARTEM-IS Spreadsheet which will allow users to describe what “other” response they have in mind.

v. Do not use values larger than 997 for codes, so that Custom and Other remain the largest values in use.

g. Input `sort` value of the item to represent its placement in the order of all response options as you want them shown in the ARTEM-IS Form. Adjust sort values of the entire response set to make sure that the response options are adequately sorted. `sort` value of the first response option to be shown in the Form should be 0, and the rest should follow in incremental order as they should appear on the screen.

i. `sort` and `value` can be the same but do not have to be the same. For example, if you are adding new preprocessing software, you will need to use an available `value` for the newly added software. Using the same `value` and `sort` value would place the new response option at the end of the list of response options, whereas it would be easier for the users to navigate the list of software names when they are sorted alphabetically in the online Form.

h. Set `active` value as 1.

i. Log changes (see General guidelines).

Adding a whole new response set

- Before adding a new response set, consider whether there is an already existing response set that you could use with or without a bit of adjustment to the old response set, without endangering version compatibility and weigh carefully what is the best option for version compatibility.
    a. For example, if the ARTEM-IS Template in the future is being extended to include frequency and time-frequency analysis, the team creating this extension may find that they can reuse some of the existing response sets that were developed for ERP analysis
- Add the new response sets as a new set of rows with one row per each response option. Response sets are sorted alphabetically (by `type` value) for easier navigation, so you will want to either add the new response set in the appropriate place, or, alternatively, to add the response set at the end of the table, and then sort the table by `type`.

- Choose `type` value for the response set. `type` value is like a variable name for the response set (e.g., `boolean` for yes-or-no questions) and it should represent what the response set is about. When choosing `type` value, keep in mind different potential uses of the response set (e.g., some of the filter settings are the same for online and offline filters, so the response sets for these questions do not contain “offline” or “online” in their `type` name).
  a. Make sure that the `type` value **is not already taken** by another response set, even if this response set has been discontinued in the current version of ARTEM-IS to ensure compatibility between versions.
  b. Start `type` name with a letter and use only lowercase letters, numbers and “_” character for `type`, like when you are creating variable names in programming
- Input `name` values for each option.
  a. If you have an option that is essentially “other”, try to formulate the whole response set so that the response text (`name`) can be literally just “other” (not for example “something else” or “other solution”) to maintain consistency across different response sets.
- Input `value` for each option. They can either start with 0 and increase incrementally, or you can designate meaningful values or value ranges.
  a. For example, you can designate `value` ranges to have different meanings (e.g., codes 101-199 for one group of responses, and codes 202-299 for a different group of responses).
  b. If one of the responses is “other”, its `value` should be 999. If the new response option refers to a custom-made solution, such as custom processing scripts or electrode layouts, its `value` should be 998. Do use these codes for Other and Custom answer options, and **do not use** them for anything else.
  c. If you have added an option to respond “Other”, make sure to also add an open-ended text sub-question to the ARTEM-IS Spreadsheet which will allow users to describe what “other” response they have in mind.
- Specify the `sort` value for each option to designate the order in which items will appear in the online Form. These values should start with 0 and increase incrementally.
- Log changes (see General guidelines).

Discontinuing a response option or a whole response set from use

- Set the `active` value of all discontinued options to be 0.
- If you are discontinuing an entire response set, make sure that it is not used by any questions in the ARTEM-IS Spreadsheet that you haven't considered.
- **Do not** delete the rows containing the discontinued options.
- **Do not** reuse `id` of discontinued response options or `type` of discontinued response sets for new response options or sets.
- If a response option or response set is discontinued, **this will inevitably create a version-compatibility challenge** in the sense that the Web App will be unable to show the answer to this question for the existing online Reports where this option has been selected (the question will appear as if it was unanswered). Meanwhile, the

answer will still be preserved in the Web Application backend. This issue would not affect downloaded Reports or Reports which have not checked the discontinued answer option. Therefore, removing an answer option should only be considered if really necessary.

- Log changes (see General guidelines).

Changes to an existing response option

- Only change the `name` of a response option if you are doing minor rephrasing. **Do not** change the meaning of a response option, even subtly, to ensure compatibility between versions of ARTEM-IS. Otherwise, the old Reports where this response option has been used will no longer reflect the specific intention of the researchers who filled in the Report.
- If the change that you are planning to introduce is altering the meaning of a response option, you should discontinue the old option and add a new one (or more of them) in its place.
- Log changes (see General guidelines).

Rearranging the order of response options

- Sort allows you to sort response options in the online Form in any way you want without changing unique response codes (`value`).
- Input `sort` value for each option to designate the order in which items will appear in the online Form. These values should start with 0 and increase incrementally.
- You can safely rearrange item order without any consequences for compatibility.
- To make the Presets data sheet easier to browse, make sure that table rows within one response set are sorted by their `sort` column value.
- Log changes (see General guidelines).

Changes to the ARTEM-IS Spreadsheet (questions list):

Keep in mind that the *order of columns in each sheet of the ARTEM-IS Spreadsheet may vary from one sheet to another*; this does not affect the functionality.

Adding new questions (reporting items)

- Adding new questions does not pose a compatibility challenge by itself
- Steps to add a new question:
    a. Add a new row to the appropriate sheet (section) in the ARTEM-IS Spreadsheet, in such a way that response options are sorted in the same order as they will appear on the screen.
    b. Type in the same `sub_section` value as the rest of the subsection has. If this question will be part of a new subsection, type in the novel subsection name in this field
        1. Make sure there are no typos or minor differences in the text, the field value needs to be exactly the same as in the case of other questions in the same subsection!
    c. If the new question is now the first question in a section, move the `preamble` contents to this row

d. Type in question text as you want it to appear in the online Reports and PDF Reports in the `question` column
   1. Tips for formulating question text:
      1. Be as brief as possible - if you can use a phrase rather than a whole sentence that would be ideal (e.g., use “Study abstract” rather than “Please insert study abstract here”)
      2. If you see that the text is becoming too long, consider moving part of the instructions to the `details` column
      3. Do not use “Please” when using imperative tense
e. Type in additional instructions how to correctly provide answers, details, and any other information that you think may help users into the `details` column
f. Choose appropriate `field_type` value (see ARTEM-IS Template Specification for options)
   1. If you have a multiple-choice type of question, opt for drop-down menu variants if there are more than 6 options
g. If the question is multiple-choice in any form (`radio`, `radio_multiple`, `select`, `select_multiple` types), type in the name of the response set that should be used with this question. The name of the response set is the `type` value for this response set in the Presets data sheet.
   1. For other types of questions, leave this cell empty.
   2. If needed, create a new response set in the Presets table (see Changes to the Presets sheet).
   3. If one of the answer options for this question is to select “Other” make sure to also create a new subquestion allowing the user to define what “other” response they have in mind.
h. Type in measurement unit in which you expect responses in the `unit` column, if appropriate (for example, Hz or dB/octave).
   1. The unit will be displayed next to the response field in the Form.
   2. If this question does not require that the user fills in numerical values in a specific measurement unit, leave this field blank.
   3. Opt for one measurement unit if there are multiple options for measurement units you could choose from (for example, dB/octave and dB/decade or ms and s). Alternatively, you can ask a separate multiple-choice question about the measurement unit if you want to allow users to choose a measurement unit in line with their preference.
i. Choose `item_pref_label` value for the question. `item_pref_label` value is like a variable name for the response set (e.g., `study_title` for the title of the study being described) and it should be very brief and represent what the question is about.
   1. Make sure that the `item_pref_label` value **is not already taken** by another question, even if this question has been discontinued in the current version of ARTEM-IS, to ensure compatibility between versions.

2. Start `item_pref_label` name with a letter and use only lowercase letters, numbers and "_" character for `item_pref_label`, like when you are creating variable names in programming

j. Set visibility rules for the question in the `visibility` field. If the question should always be displayed, specify 1. If it is a subquestion of a different question, design a logical expression whose value will be true or 1 when the question should be displayed and false or 0 when the question should not be displayed (e.g., study_working_title_exists == 1).
   1. This should be done in the JavaScript programming language.
   2. **Be careful!** Minor typos in the `visibility` field (for example typing just = rather than == when checking for equality) can prevent the question from displaying correctly.
   3. **Do not** set up question visibility rules so that visibility of a question in one section depends on a response to a question in a different section without contacting the Web App Admin, as this will require changes to the Web App. For simplicity's sake, we recommend avoiding this if possible.

k. Set `item_order` for this question to contain the same item_order formula as all the other rows

l. Set `include` to be 1.

m. Set `mandatory` to be 1. **This column is currently not in use**, and adding "1" to the field is just keeping the new questions the same as the existing one.

n. Set `activity_pref_label` and `activity_order` to be the same as in the case of all other rows in the section where the question is placed.
   1. Make sure there are no typos in `activity_pref_label`, the field value needs to be exactly the same as in the other rows of the sheet!

o. You can skip the BIDS fields for now, they are not currently in use.

p. Log changes (see General guidelines).

Adding optional questions or making questions optional

- There are currently no mandatory questions in the technical sense that the Report cannot be downloaded or shared before answering them. All questions are optional in this sense.
- However, you may also want to create questions that are optional in the sense that the user is simply offered to answer them, and if they select "no", these questions do not count towards the percent of Report completion. In ARTEM-IS v2.0, we did this for all questions that are not required by the corresponding guidelines for good reporting practice, and the same practice is retained in v2.1.
- You can add an optional question by first adding a preceding "yes-or-no" question, asking the user whether they are interested in providing this additional information. Then, you can set visibility of the optional question to depend on the answer to the yes-or-no question. If the user selects "no", they will not be asked the optional question and it will not count towards the percent of Report completion.
- If you are making an existing question optional, you will want to preserve existing responses. You can do that only in cooperation with the Web App Administrator, but you could ensure that all existing Reports created in the old version of the Template have "yes" pre-selected to the novel preceding "yes-or-no" question, when updating

the Template to the new version. This would allow responses existing in the old Reports to be preserved and displayed.

Removing questions from the Template

- Set `include` value of the discontinued question to be 0.
- If it is a multiple-choice question, and the response set tied to this question is no longer in use after removing this question, set `active` value of all discontinued options to be 0.
- **Do not** delete the rows containing the discontinued question or response set.
- If a question is discontinued, **this will inevitably create a version-compatibility challenge** (see also Naming Template Specification versions): all existing responses to this question will be erased from the Web Application backend when updating the Template to the new version.[5] Obviously, all downloaded Reports that contain an answer to the discontinued question would still have them. If a user tries to upload a JSON file with such a Report, the responses to the discontinued question would simply not be loaded to the online platform. This would not affect downloaded Reports or online Reports in which the Contributors had skipped the now discontinued question. Therefore, be careful when deciding to remove responses to a question.
- If the question is being discontinued to make an improved version of the same question in a way that cannot be safely achieved by simply changing the existing question, consider still keeping the old question while creating the new ones at the same time, if possible.
    a. For example, you might be discontinuing a broad open-ended question to introduce a list of more specific, shorter questions, which is indeed a better solution (see ARTEM-IS Design Guidelines). If so, consider whether the existing open-ended question can be retained as a question to provide an additional verbal description, which can be made optional if appropriate.
- Be careful if you are removing all questions in a subsection, because this leads to disappearance of a subsection altogether. Such a change should be implemented while coordinating with the ARTEM-IS Web App Administrator to ensure that no issues arise.
- Log changes (see General guidelines).

Changes to an existing question

- Only make changes to the `question` field if you are doing minor rephrasing which will not change the meaning of the question. If this is not what you have in mind, you should create a new question instead, and possibly discontinue the old one.
- The same goes for the `details` field - you can add clarification and examples, but you cannot change the meaning of the reporting item or the expected formatting of

[5] Currently, when loading ARTEM-IS Specification, the Web App skips questions which have been discontinued and it does not create columns for them in the database table in the backend. Note that this means that the Web App can also be set up to retain the column on discontinued items. For example, it can be set up to simply not display this item in the online form and in the Reports, while retaining the information in the backend. Moreover, with additional development, the Web App can be set up to handle and display different versions of an ARTEM-IS Template. Over time, however, both options might make the Web App require considerable resources for running.

the response for text questions, if the reporting item already has expected formatting (e.g., whether the reference should be provided as a DOI, APA-style, etc.)

- You should make a new question and discontinue the old one if you want to change the expected response type (`field_type`), unless you are making a change where there will be no loss of information, such as a change from integer numbers to decimal numbers, or from allowing a single response to allowing multiple responses. Even in these cases, consult the ARTEM-IS Web App Administrator first to ensure that there are no issues with compatibility which may arise from the way response values are saved in the backend table.
- **Do not** change the measurement unit in the `unit` field, as this will invalidate existing responses. If you think it is necessary to allow expressing values to existing questions in different measurement units, you should allow for the specification of alternative units rather than changing the measurement unit of this particular reporting item.
    a. The preferable way to do that:
        1. Create a preceding multiple-choice question which will enquire about the unit in which the users want to report the value in question. One of the options offered in the multiple-choice question needs to be the old measurement unit, and you can add any additional measurement units you think are necessary.
        2. Work with the ARTEM-IS Web Application Administrator to edit all existing Reports filled in the old version of the Template, so that their response to the multiple-choice question is set to the old measurement unit if they contain a response to the question you want to change.
    b. Alternatively, you can:
        1. Create a preceding multiple-choice question which will enquire about the unit in which the users want to report the value in question. One of the options offered in the multiple-choice question needs to be the old measurement unit, and you can add any additional measurement units you think are necessary.
        2. Set the visibility option of the old question so that it is shown only if the old measurement unit is selected in the multiple-choice question. This way, the old question will contain responses provided only in the old measurement unit.
        3. Create a new question for each new measurement unit, and make its visibility contingent on selecting this measurement unit when responding to the multiple-choice question.
        4. Like in the other scenario, you should work with the ARTEM-IS Web Application Administrator to edit all existing Reports filled in the old version of the Template, so that their response to the multiple-choice question is set to the old measurement unit if they contain an answer to the question you want to change.
    c. Both of these options are valid as the combination of the answer to the multiple-choice question about the unit and the follow-up question(s) where the measurement value is provided allows identifying the correct combination of value + measurement unit. **The first option is preferable whenever**

possible, as it makes the Template simpler and takes up less space in the backend database table of the Web Application.

- If you want to replace the response set with a new one in the `choices` column, this will create a **compatibility challenge** (see also Naming Template Specification versions) in the sense that all existing responses will contain response codes corresponding to the old response set. You should either discontinue this question and create a new one instead, or look into alternative solutions that do not pose compatibility issues.
- You can make changes to the `visibility` column, but it is essential to ensure that this question is set to be visible in all existing Reports which contain a response to the question. You can achieve this by designing the visibility conditions carefully and working with the Web App Admin to adjust response values in existing Reports. For an example of how to do this, see adding optional questions.
    a. Please refer to the instructions for adding new questions for general advice on how to set up visibility rules.
- For changes to the `sub_section`, see here.
- **Do not** move a question to a different section (tab).
- Log changes (see General guidelines).

Adding a new subsection and rearranging affiliations of questions with subsections

- `sub_section` value is not a variable name and it does not need to adhere to programming variable name convention (for example, you can use space to separate words).
- Adding a new subsection does not pose a compatibility challenge. To add a new subsection, either by separating existing questions into smaller subsections or because you are adding a new group of questions, you should simply input the desired name of the new subsection to the `sub_section` column for these questions.
    a. Make sure that all questions that belong to the same subsection have a completely identical `sub_section` value.
- To rearrange which existing questions belong to which subsections, simply input the desired `sub_section` value.
    a. The only situation where you need to be careful is if rearranging questions leads to disappearance of an entire existing subsection, and such a change should be implemented while coordinating with the ARTEM-IS Web App Administrator to ensure that no issues arise.

Rearranging order of questions within a section

- Rearranging the order of question within a section can be done without issues by adjusting the order in which the questions are sorted in the rows of the ARTEM-IS Spreadsheet table and ensuring that the correct formula is in the `item_order` field
- The only thing to keep in mind is that the questions in the same `sub_section` need to be grouped together, i.e., to have consecutive `item_order` values.
- Rearranging question order can sometimes lead to inadvertently leaving some questions without `item_order` value and similar errors. After you are done with

rearranging questions, make sure that all questions in the tab have the correct `item_order` formula in them.
- Log changes (see General guidelines).
- If the previously first question in a sheet is no longer the first, ensure that the `preamble` text and the `Change_log` information related to the entire sheet are moved to the first row again.

Adding an entire new section

- In principle, adding a new section to the ARTEM-IS Template does not pose a compatibility issue.
- The new section can be added by creating a new tab in the ARTEM-IS Spreadsheet and by populating the column titles and contents following the rules described here. Existing ARTEM-IS Spreadsheet tabs can serve as a template for creating a new tab.
    a. Make sure that the columns that are filled in using a formula in the existing sections, such as `item_order`, contain the same formula as in other tabs.
    b. Choose a unique `activity_pref_label` for the new section.
        1. Make sure that the `activity_pref_label` value **is not already taken** by another section, even if this section has been discontinued in the current version of ARTEM-IS to ensure compatibility between versions.
        2. Start `activity_pref_label` name with a letter and use only lowercase letters, numbers and "_" character for `type`, like when you are creating variable names in programming
- Important note! We have not had an addition of a new section to the ARTEM-IS Web Application so far. Therefore, uploading the new section to the ARTEM-IS Web App needs to be coordinated with the Web App Administrator, it cannot be simply done by the team members who have update rights. This is to ensure that no issues arise in the process.
- If you are not adding the section at the end of the Template, but you want to place it in between two existing sections, please also refer to the section on adjusting activity order.
- Log changes only in the first row of the sheet (see General guidelines).

Changing order of existing sections

- In the ARTEM-IS Spreadsheet, this is done by adjusting `activity_order`.
- Important note! While this should not cause compatibility issues in principle, we have not rearranged sections in the ARTEM-IS Web Application so far. Therefore, such a change needs to be done in consultation with the Web App Administrator, it cannot be simply done by the team members who have update rights. This is to ensure that no issues arise in the process.
- Log changes only in the first row of the sheet (see General guidelines).

Separating questions in one section into multiple sections or moving a question from one section to another

- While this should not cause major compatibility issues in principle, it **should be avoided** as a general rule to keep the structure of the Template consistent between

versions, which is especially helpful for machine-readability. Only consider it if really necessary.

- Important note! We have not done this in the ARTEM-IS Web Application so far. Therefore, such a change needs to be done in consultation with the Web App Administrator, it cannot be simply done by the team members who have update rights. This is to ensure that no issues arise in the process.

Editing section description (preamble)

- Making changes to the section description can be done simply by editing `preamble` value in the first row, and it does not pose a compatibility challenge.
- Make sure that the `preamble` value is in the first row of each tab.
- Log changes only in the first row of the sheet (see General guidelines).

Important! Changes to the **Artifacts** subsection of the Preprocessing section should be implemented with extra care and in consultation with the ARTEM-IS Web App Administrator because of the way multiple iterations of the Artifacts questions are created in the database in the backend of ARTEM-IS Web App.

- It is not possible to change the number of artifact removal steps in the Web App by altering the ARTEM-IS Specification spreadsheets. The Specification includes only one set of questions, for one artifact removal step. In the Web App backend, these questions are **copied 10 times over** to create variables that can hold up to 10 artifact removal steps in the backend database of ARTEM-IS Reports. This is because the number of variables in the backend database table cannot be different for each Report - the same table needs to hold the data on all Reports. Ten has been chosen as a reasonable number of artifact removal steps - it is highly unlikely for any study to reach that number.
- The number of potential artifact removal steps in the ARTEM-IS Web App can be changed by the Web App Administrator. However, this will change the structure of the whole database of reports in the backend. Consequences of such a decision need to be carefully weighted. In any case, **the number of potential steps should not be lowered** unless the team has made sure that none of the existing reports are affected by such a change. On the other hand, increasing the number of potential steps does not affect existing Reports, but it does increase the size of the backend database.
- **Naming artifact removal steps:** each item variable name (`item_pref_label`) should start with ***artifact_1***, e.g., *artifact_1_interpolate_how*. The code in the backend recognises variables with such names as artifact variables, and it generates new variables starting with *artifact_2*, *artifact_3*, etc, up to *artifact_10*.

Updating the Web App to a new version of the ARTEM-IS for ERP Template

Currently, the Web App supports only a single Template, in one version. If in the future the need arises for simultaneous hosting of multiple Templates or multiple versions, this will require additional development.

Updating from one version to the next is done by designated ARTEM-IS Team members, who are given access to the update feature. Because there are two types of data sheets in the ARTEM-IS Specification, it is possible to make three types of updates - (1) update of the ARTEM-IS Spreadsheet; (2) update of the Presets spreadsheet, (3) update of both simultaneously.

The update itself takes place in three steps (see Figure 14).

1. In the first step, the desired ***Specification Google Sheets are imported from Google Sheets*** to the ARTEM-IS Web App backend using Google's unique ID of a Spreadsheet (the unique ID part of the URL link). Please have in mind that the spreadsheets need to be publically available through the provided link for this step to work.
2. In the next step, ***tables are compared and a report listing all changes that would take place is automatically generated*** so that the team member updating the template can check it and ensure that undesired changes do not happen. To facilitate this, critical changes are flagged with orange and red flags in the automatic report. For example, a change of the expected answer type, such as switching a question from open-ended to multiple-choice, will be flagged with an orange flag, while trying to use a response set that does not exist in the Presets sheet will result in a red flag.
3. In the third step, ***the ARTEM-IS Template update is implemented***. After this step, there is no going back unless a backup of the database has been made by the Web App Administrator prior to this step, so this is a recommended course of action, especially for larger updates. When implementing the change, **the new version number should be added in the appropriate field, together with the subversion number.**

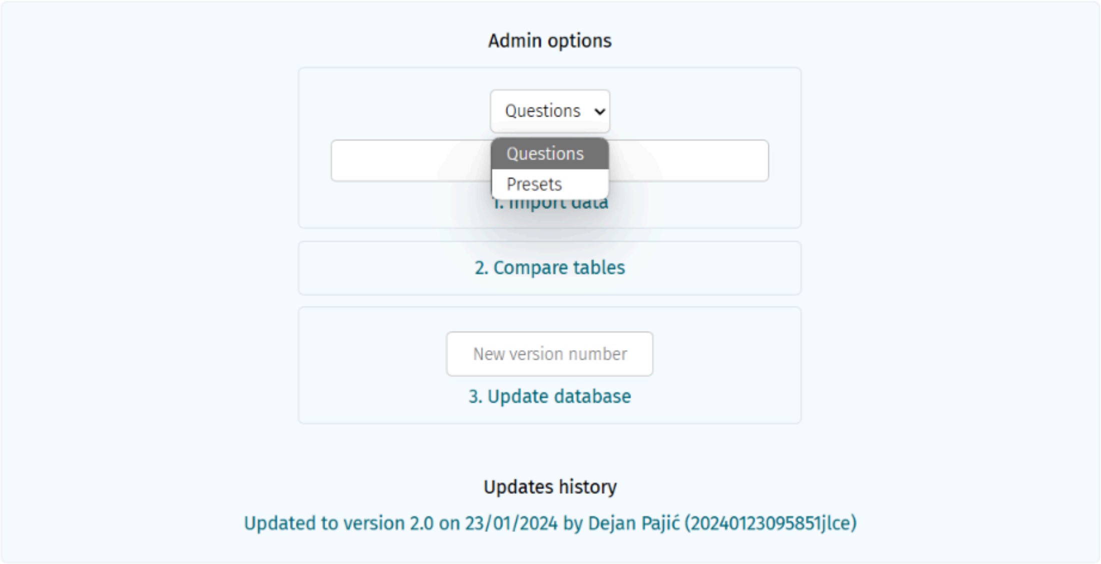

Figure 16. The three steps of updating the ARTEM-IS version.

Keeping track of changes between Template versions

There are two ways to keep track of changes between two versions of an ARTEM-IS for ERP Template.

Firstly, ARTEM-IS Web App keeps an automatically generated changelog which lists all changes between the previous version and the latest one. Automatically generated changelogs for each version can be found in the Web App itself (on this page: https://artemis.incf.org/help) and a permanent copy of them can be found on the OSF (https://osf.io/hcmgt).

In addition, ARTEM-IS Specification spreadsheets both include a Change_log column in which change to each field is documented and explained briefly, starting from updates from v1.0 to v2.0 (the column was a new addition to ARTEM-IS v2.0). This column is easier for a typical reader, includes a rationale and centralises all changes to the item within one update, as well as from different updates (conversely, automatic change log groups each type of change together, so as a result changes to response option text and adding new response options in the same question are in separate sections of the report).

Citing ARTEM-IS

If you used the ARTEM-IS for ERP Web App for your research, if you wish to refer to the ARTEM-IS for ERP Template, or if you wish to cite information in this document, please cite the following publication, to which this document is a supplement:

> Šoškić, A., Kovic, V., Algermissen, J., Fischer, N. L., Ganis, G., Gau, R., … Styles, S. J. (2023, January 5). *ARTEM-IS for ERP: Agreed Reporting Template for EEG Methodology - International Standard for documenting studies on Event-Related Potentials.* https://doi.org/10.31234/osf.io/mq5sy

If you wish to refer to the general ARTEM-IS concept or project, the design principles behind the project, or the ARTEM-IS Statement, please cite the following publication:

> Styles, S. J., Ković, V., Ke, H., & Šoškić, A. (2021). Towards ARTEM-IS: Design guidelines for evidence-based EEG methodology reporting tools. *NeuroImage, 245,* 118721. https://doi.org/10.1016/j.neuroimage.2021.118721

Licence

The **ARTEM-IS for ERP Template** is licensed with a Creative Commons Licence (CC) with an obligation for attribution (BY), for non-commercial uses (NC), and with the obligation that others will also share their resulting work with an equivalent licence (SA) (i.e., CC-BY-NC-SA). In addition, we allow educational uses such as in higher education or commercial training courses.

The **ARTEM-IS Web Application** is currently not open source, but we are committed to making it open in due course. Please note that the ARTEM-IS Web App is intended to be a

tool that facilitates creating and sharing ARTEM-IS Reports. As such, it derives all of the essential functionality directly from the ARTEM-IS Template Specifications, which are openly available as stated above, and which can be used to create and share ARTEM-IS-compliant reports independently of the Web App. The only intellectual property that is not shared is the code for displaying the user interface and managing reports in the backend of the ARTEM-IS official Web Application on the International Neuroinformatics Coordinating Facility (INCF) platform (https://artemis.incf.org/).

ARTEM-IS Extensions

Here are some examples how the ARTEM-IS Template Specification can be used in the future, while adhering to its licence (see Licence):

(1) **Translation of ARTEM-IS for ERP into other languages.**
If you are interested in creating an official translation into another language that we would endorse, link to, or possibly make available on our Web App, please get in touch.
You can, of course, also create and host independent and unofficial translations on your own platform, which will be made easier once the Web Application code is open source, too.

(2) **Develop your own ARTEM-IS Templates or ARTEM-IS inspired Templates, as well as extensions and upgrades to ARTEM-IS for ERP.**
If you want to join the ARTEM-IS Working Group and develop future ARTEM-IS Templates for EEG or to expand ARTEM-IS beyond EEG, please get in touch.
In addition, you can create new reporting tools inspired by ARTEM-IS. In the latter case, the resulting templates should not be called ARTEM-IS Templates to avoid confusion. The ARTEM-IS source should be appropriately credited.

(3) **Make your own ARTEM-IS for ERP Web Application -** for example, an internal application for your laboratory projects or for use in education or training. We hope to facilitate this by making the code openly available in the future.

Contact

If you notice a bug or have a suggestion, please post an issue on GitHub.

If you want to join the ARTEM-IS team, reach out to one of the ICNF Working Group chairs.

If you have general questions, or are interested in collaboration, you are welcome to use the same contact route, too.

References

Keil, A., Debener, S., Gratton, G., Junghöfer, M., Kappenman, E. S., Luck, S. J., Luu, P., Miller, G. A., & Yee, C. M. (2014). Committee report: Publication guidelines and

recommendations for studies using electroencephalography and magnetoencephalography. *Psychophysiology*, *51*(1), 1–21. https://doi.org/10.1111/psyp.12147

Pernet, C., Garrido, M. I., Gramfort, A., Maurits, N., Michel, C. M., Pang, E., Salmelin, R., Schoffelen, J. M., Valdes-Sosa, P. A., & Puce, A. (2020). Issues and recommendations from the OHBM COBIDAS MEEG committee for reproducible EEG and MEG research. *Nature Neuroscience*, *23*(12), 1473–1483. https://doi.org/10.1038/s41593-020-00709-0

Styles, S. J., Ković, V., Ke, H., & Šoškić, A. (2021). Towards ARTEM-IS: Design guidelines for evidence-based EEG methodology reporting tools. *NeuroImage, 245,* 118721. https://doi.org/10.1016/j.neuroimage.2021.118721

Šoškić, A., Ković, V., Algermissen, J., Fischer, N. L., Ganis, G., Gau, R., … Styles, S. J. (2023, January 5). *ARTEM-IS for ERP: Agreed Reporting Template for EEG Methodology - International Standard for documenting studies on Event-Related Potentials.* https://doi.org/10.31234/osf.io/mq5sy
