# Supplementary material for: ARTEM‐IS for ERP: Agreed Reporting Template for EEG Methodology—International Standard for Event‐Related Potential Experiments
Source: Psychophysiology. 2025 Dec 8;62(12):e70187. doi: 10.1111/psyp.70187 (PMC12683983; doi:10.1111/psyp.70187)
Supplement: Supplementary file 2 — Data S2: psyp70187‐sup‐0002‐Supinfo2.pdf. [file PSYP-62-e70187-s004.pdf]

Web Application User Manual

Created on: November 2025
ARTEM-IS version described: ARTEM-IS for ERP v2.1

V2.0
Written by: Anđela Šoškić, Vanja Ković, and Dejan Pajić
Reviewed by: Robert Oostenveld, Nastassja L. Fischer, Yuri G. Pavlov

V2.1
Created on: November 2025
Updated by: Anđela Šoškić
Updates reviewed by: Katarina Stekić

Contents:

- What is ARTEM-IS?
- What does this report contain and who is it for?
- Glossary
- Input and output of the Web App
    - ARTEM-IS for ERP 2.1 Template content overview
        - A brief description of each section
        - Mandatory responses vs. percent of completion
    - Output: Understanding ARTEM-IS Reports
        - PDF Report
        - DOCX Report
        - JSON Report
- ARTEM-IS Web App features from the perspective of a user
    - Landing Page
    - Creating and setting up an account
    - Signing in, signing out, and session
    - Menu options after signing in

- Adding a new ARTEM-IS Report and editing Reports
  - Creating, viewing the pre-processing pipeline
- Browsing your own Reports and editing Report metadata
  - Setting up and viewing Report Contributors
  - Private and public reports
- Browsing and viewing public Reports
- Downloading an existing Report
- Using an existing Report as a template for a new one
- Tracking down the origin of a Report
- Help/About

Reading JSON Reports
- Structure of a JSON Report
- ARTEM-IS for ERP v2.1 Template Specification
  - Links to ARTEM-IS Template Specification spreadsheets

Keeping track of changes between Template versions
Citing ARTEM-IS
Licence
ARTEM-IS Extensions
Contact
References

What is ARTEM-IS?

ARTEM-IS is a collaborative grass-roots initiative that builds web apps which support EEG researchers in creating detailed human- and machine-readable method summaries using a standardised metadata template. These summaries can be used as supplements, memory aid when writing, or to facilitate metadata extraction. The main purpose is to improve research reporting accuracy, research replicability and reproducibility, as well as to provide better grounds for meta-scientific research in the EEG domain.

You can learn more about the project and read the ARTEM-IS Statement in this paper. If you agree with the Statement, please consider supporting it with your signature by filling in this form. The more up-to-date information on the project can be found in our OSF repository. If you have further questions, or you are interested in joining the team, do get in touch.

What does this report contain and who is it for?

This report is a supplementary document to this paper introducing ARTEM-IS for ERP.

This report aims to provide necessary information to:

a) researchers who want to use the Web Application to generate or read ARTEM-IS-compliant methodology summaries
b) researchers who want to create and use JSON machine-readable methodological summaries

As such, this report includes:

- instructions on how to use the ARTEM-IS official Web Application
- instructions on how to read ARTEM-IS Reports

Glossary

ARTEM-IS Template: the standardised reporting format for describing methodological properties, i.e., all questions with expected answer formats, branching rules, etc.
ARTEM-IS Template Specification: detailed description of the ARTEM-IS Template contents and branching rules. It consists of two data sheets, ARTEM-IS Spreadsheet and ARTEM-IS Presets Spreadsheet
ARTEM-IS Spreadsheet: spreadsheet describing all questions and their properties (expected answer type, branching rules, sections they belong to, presentation order, etc.)
ARTEM-IS Presets Spreadsheet: spreadsheet describing response options that are offered to multiple-choice questions (list of options, order of presentation, numerical codes, etc.)
ARTEM-IS Web Application (ARTEM-IS Web App): the online web application provided by the ARTEM-IS team and hosted on the INCF server, that allows generating, sharing, storing, and browsing methodological descriptions that are structured according to the ARTEM-IS Template (ARTEM-IS Reports)
ARTEM-IS Form: the online form that is available on the ARTEM-IS Web App that the users fill in to generate methodological descriptions structured according to the ARTEM-IS Template (ARTEM-IS Reports)

ARTEM-IS Report: the methodological description structured according to the ARTEM-IS Template, available for download in PDF or JSON formats through the Web App (ARTEM-IS PDF Report, ARTEM-IS JSON Report)
Question/Reporting Item: one reporting item in the template (appears as one question in the ARTEM-IS Form)
Response set: set of options that are offered to a multiple choice question
Response option: one option within a response set that can be selected when there is a multiple choice question

Input and output of the Web App

Currently, there is one ARTEM-IS **Template**: ARTEM-IS for ERP. The ARTEM-IS **Web App** is currently designed to allow generating, sharing, storing, and browsing methodological descriptions that are structured according to this Template.

The main input of the Web App is the online questionnaire, called **ARTEM-IS Form**, that allows users to generate standardised descriptions in line with the Template. The main outputs of the Web App are **ARTEM-IS Reports**, which can be viewed online or downloaded in JSON, DOCX, or PDF formats (JSON Reports, DOCX Reports, and PDF Reports).

In this section, we will first provide a brief overview of the contents of the ARTEM-IS for ERP Template, and consequently, the online Form in the Web App. Next, we will describe the contents of the PDF and JSON Reports and how these should be interpreted.

ARTEM-IS for ERP 2.1 Template content overview

The current version 2.1 of the ARTEM-IS for ERP Template comprises nine sections designed to assist researchers to report the following aspects of a study: (1) study description, (2) experimental design and sample, (3) hardware, (4) acquisition, (5) pre-processing, (6) measurement, (7) channel selection for analysis, (8) visualisation, and (9) other. The description of the statistical analysis is not included in this version. Each section contains a set of questions; the answers can be categorical, numerical or short open-ended textual statements. Sections are separated into subsections for easier navigation.

A brief description of each section

1. Study: This section gathers information about general aspects of the research study: information related to whether a Report documents a planned pipeline or an already applied one; the title of the study; information about the authors; related publications and DOIs; associated available datasets and supplementary materials (e.g., code); supplementary materials; licensing; abstract; keywords; financial support; ethics committee approval; acknowledgements; and citation instructions.

2. Experimental design: This section gathers information about the experimental design and the sample of the study: the number of participants in the study; inclusion and exclusion

criteria; information related to the experimental/comparison groups; details on the trials presented and analysed; and software for stimuli presentation.

3. Hardware: This section provides information about the hardware used for data acquisition: information related to the EEG cap/net; electrodes characteristics and placement scheme; information on the amplifier and configuration; description of triggers (e.g., how they were generated and saved); and information related to any additional devices used (e.g., signal boxes, converters, electrode position measurement devices).

4. Acquisition: This section documents information related to the data acquisition process: acquisition software used and version; details on impedances or alternative data acquisition quality measures; references for EEG, EOG and other electrodes, if used, and their alternatives (e.g., Driven Right Leg (DRL)); ground electrode and placement; EOG channels for recording eye movement-dependent voltage and placement; online high pass and low pass filters and notch filters; and acquisition sampling rate.

5. Pre-processing: This section documents pre-processing, i.e., software used for pre-processing, the steps in the pipeline before measurement and statistical analysis of an ERP component: automated preprocessing pipeline; offline filtering; downsampling; re-referencing of EEG, EOG and other channels, if used; artifact removal methods in each elimination step, if there were more than one (rejection of bad trials, data segments, or channels, artifact correction, channel interpolation, multi-step automated approaches); epoching;, baseline correction; other steps. The user can add as many steps as they want in the order they are arranged in the pipeline and at the end of this section, the user is asked whether they would like to additionally self-describe the order of operations in a free-text field in which pre-processing steps were applied.

6. Measurements: This section offers options to describe amplitude and latency measurements of one ERP component, unless the user subjected all time points and channels to statistical analysis (e.g., in a mass univariate analysis). Amplitude properties include: measurement software, waveforms used to measure amplitude, amplitude measure (peak, mean, window area, etc.) and its parameters, measurement time window, rationale of selecting this exact time window. Similarly, latency properties include: measurement software, waveforms used to measure latency, midpoint latency measure (e.g., local peak, 50% area) - if applied, onset latency measure (e.g., fractional area, fractional peak) - if applied, the appropriate parameters of each latency measure depending on the choices, time window within which the latency was searched for, and the rationale for selecting this exact time window.

7. Channels: This section gathers information about the selection of channels for later statistical analysis. Like in the case of the previous section, the current version of the template supports describing the location for the measurement of one ERP component. There are two main scenarios - all channels are included in statistical analyses (e.g., in a mass univariate approach), or a subset of channels is included in the analyses. Within the second scenario, five options are offered: a priori selection of channels; data-driven selection of channels from the entire scalp; data-driven selection of channels from an a priori selected broader region on the scalp; selection/identification of channels in two data driven steps (e.g., maximal effect within a visually identified broader region); other. Depending on the

choice, an appropriate subset of questions follows. In the end of this section, the user is asked to describe whether the channels were entered separately into statistical analysis or if they were aggregated into regions before conducting the statistical analysis.

8. Visualisation: This section provides information related to the visualisation: the type of plot created (e.g., line plot (waveforms), topoplot, ERP grid, butterfly plot); whether any extra pre-processing was performed for visualisation purposes (e.g., smoothing filter, different baseline); description of what the data represents (e.g., single waves, difference waves) and corresponding units (e.g., voltage maps, normalised voltage maps); description of which conditions or difference waves were shown; channels selected for visualisation and the rationale for this decision, beginning and end of the time window visualised and the rationale for selecting this time window.

9. Other: This section contains an option to add additional comments, if there are any.

For a detailed overview of all the items of the checklist, see ARTEM-IS Spreadsheets below.

Mandatory responses vs. percent of completion

ARTEM-IS does not include rules on which items in the Template are mandatory to fill in, though the ARTEM-IS Template Specification has a field that allows for adding this option in the future. This is because the goal of the ARTEM-IS project is not to enforce providing a particular set of information. Our goal, instead, is to help researchers be more detailed and precise in their descriptions, as well as to help readers have an easy and transparent overview of which (and how much) of the necessary information has been provided.

As a result, the ARTEM-IS Web App also does not enforce answering any questions in the online form. Each Report can be saved, downloaded, and publically shared in any stage of the progress of filling out the Form. Instead, the readers of online Reports shown in the Web App and downloaded Reports are provided information on the **percent of completion of a given Report.**

The calculation of this proportion excludes items that are skipped due to the branching logic of the Template. More specifically, in some cases, there are sub-questions that only appear if a specific answer to a previous, “main” question has been selected. In these cases, the proportion calculation includes sub-questions only if they have been revealed to the user after selecting the appropriate response to the main question. The calculation excludes these sub-questions if the sub-questions have not been revealed to a user, either because they have provided a different answer to the main question, or because they have skipped the main question altogether.

Notably, most questions which are part of the Template are described as necessary to provide in a research report by contemporary guidelines for good practice in EEG research (e.g., Keil et al., 2014; Pernet et al., 2020). ARTEM-IS Template clearly separates the few reporting items that are not described as mandatory by the existing guidelines for good practice (e.g., additional filter properties which researchers may want to provide on top of the minimal reporting guidelines). In these cases, the researchers are first asked whether they would like to include this additional information. They are then asked to provide these

additional details only if they select “Yes”. If they select “No”, they skip these additional questions. This also means that skipping them does not influence the percent of completion calculated by the ARTEM-IS Web App.

Output: Understanding ARTEM-IS Reports

PDF Report

A PDF Report, shown in Figure 1, is made to be easier to understand by human readers, with full text of both the questions and answer options displayed in the report.

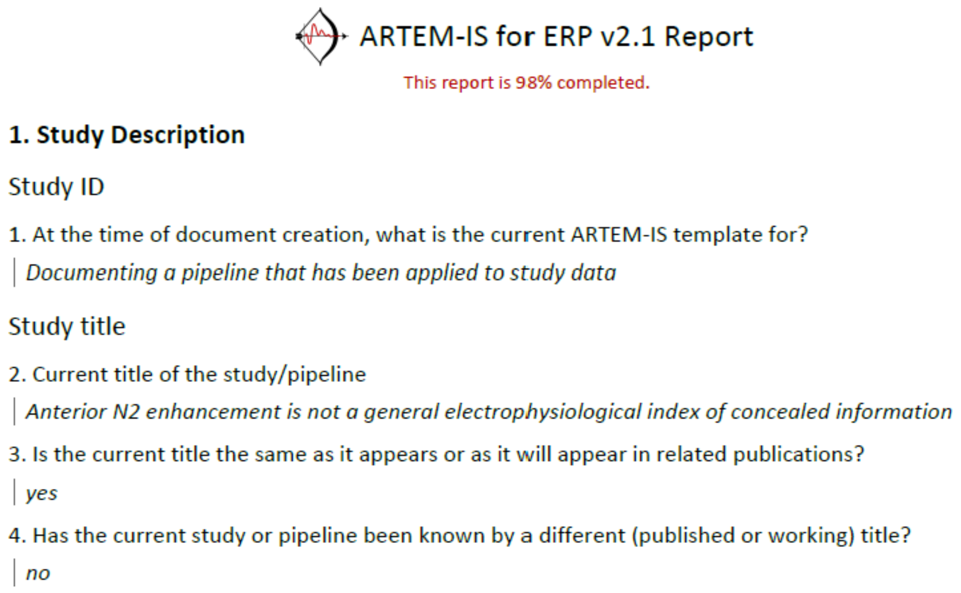

ARTEM-IS for ERP v2.1 Report

This report is 98% completed.

1. Study Description

Study ID

1. At the time of document creation, what is the current ARTEM-IS template for?

Documenting a pipeline that has been applied to study data

Study title

2. Current title of the study/pipeline

Anterior N2 enhancement is not a general electrophysiological index of concealed information

3. Is the current title the same as it appears or as it will appear in related publications?

yes

4. Has the current study or pipeline been known by a different (published or working) title?

no

Figure 1. Example ARTEM-IS for ERP v2.1 Report in PDF format.

This report consists of:

- Information about the percent of items that are completed in the Report.
- A list of all sections, with subsections clearly shown, and with a list of all pre-processing steps showcasing the order of operations in the pre-processing pipeline. Within each section, only the questions that are included according to the previous answers and branching logic are shown. If a question is not included in the branching structure of the Report, the Report reader will be alerted by an appropriate statement (e.g., *“Conditional questions 8 to 11 are not displayed.”*). Similarly, if a user filling in the report has omitted to answer a question that is shown to them, the response to this question will state: “*Answer not provided.*”
- At the end of a Report, there is information on (a) Contributors to the Report, (b) licence of the Report, (c) link to the online version of the report, (d) download date, and (e) a note if the Report is created by copying a different Report which has been used as a template to create this Report (a note stating *“**Derivative.** This report is a derivative of [Report Title] by [Contributors]. [link to the original Report that has been used]”* is provided at the end of the Report).

- If needed, the unique `Report ID` of a Report in PDF format can be extracted from the link to the online version of the Report provided at the end of the PDF document.

DOCX Report

The DOCX format offers the same content as the PDF Report, but with a complementary purpose. While PDF offers consistent formatting and wider accessibility, DOCX is more suitable for copying and reusing content.

JSON Report

Unlike PDF Reports that focus on human readability, JSON Reports, shown in Figure 2, are made with machine readability and version compatibility in mind. Structured JSON objects, unlike, for example, CSV files, provide a convenient way to store hierarchically organised, machine-readable information as a set of key/value, i.e., question/response pairs. Additionally, JSON files are exported in the so-called "pretty-print" format which enables users to relatively easily search and browse through the template structure in a text editor. Finally, the JSON format provides a convenient way for users to export and import information and build their own Reports upon the already existing privately shared or public Reports (see Using an existing Report as a template for a new one).

ARTEM-IS Web App features from the perspective of a user

In the section that follows, we will assume that the user is accessing the Web App from a PC browser, but the Web App is also designed to be accessible from a mobile device with a small screen, like a mobile phone. .

Landing Page

When first opening the ARTEM-IS Web App, a dynamic, expandable visualisation showing the branching structure of all multiple-choice questions is shown on the landing page, as shown in Figure 3. Each multiple-choice question and sub-question is represented by a node. By clicking on the nodes, the user can expand and hide their branches.

The only other thing is the menu in the top right, which allows registering or signing in to access the Web App contents (creating and browsing Reports), which are available only to registered users, as well as the About/Help page.

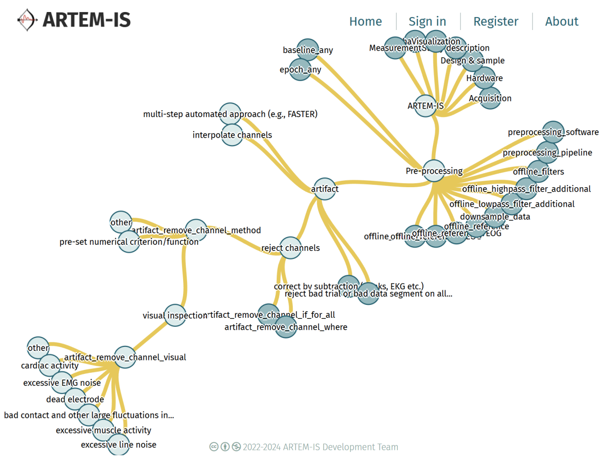

Figure 3. ARTEM-IS Web App landing page

Creating and setting up an account

Before using ARTEM-IS Web App and accessing CC-BY Reports on the platform, researchers need to create an account first by clicking on the option **Register** in the top right corner of the screen. Registration is necessary since the user is responsible for the decision on who would be able to see, download, and/or modify their reports.

To allow additional privacy and easier login for researchers, two options are offered:

1) registration with an email address
2) registration via ORCID account

Registration form is provided in Figure 4. The Registration form is straightforward and requires users to insert personal name(s), email address and a password, while family name(s), affiliation and ORCID are optional.

Researchers who wish to register with ORCID are offered this option at the top of the registration form, and they should follow ORCID instructions upon clicking on the link offering ORCID registration.

Whichever option the researchers select when registering - ORCID-based or email-based, they can add the other one later to allow both, if desired.

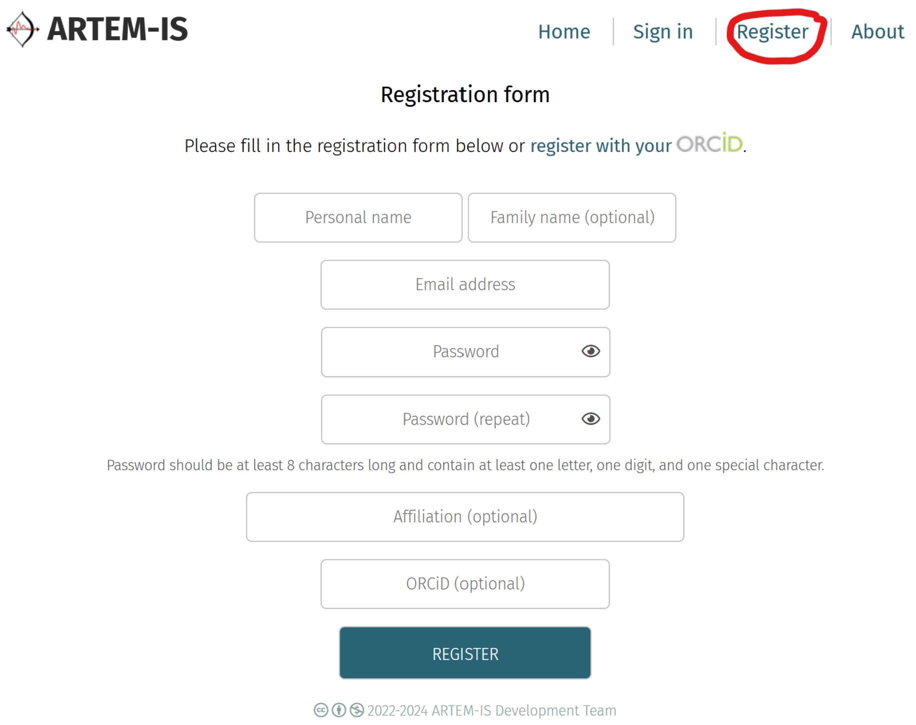

Figure 4. Registration form

The chosen settings can be changed later by visiting **My profile** option in the menu on the top right of the screen.

Signing in, signing out, and session

Once a user account has been created, it can be accessed using the **Sign in** option, which allows users to access their account via email-based login or via ORCID (Figure 5).

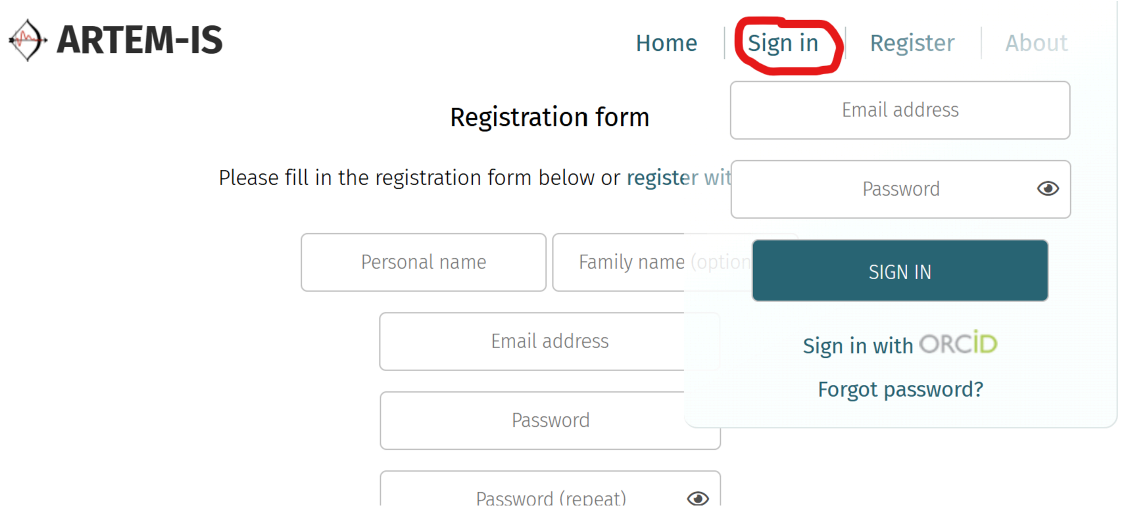

Figure 5. Signing in

Conversely, signing out is done by clicking on the door-and-arrow exit logo at the top right corner of the screen (Figure 6). Additionally, the login session is set to automatically expire after a period of inactivity.

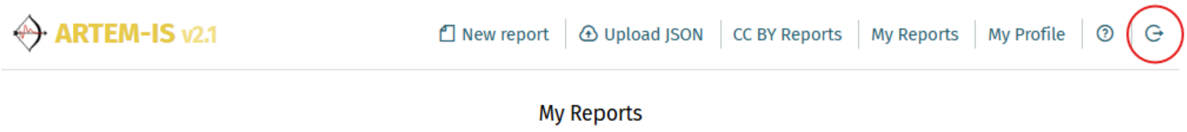

Figure 6. Signing out

Menu options after signing in

Upon signing in, the menu on the top right changes to show the following options (See Figure 7):

- **Create:** create a new Report by starting an empty form
- **Upload JSON**: upload an existing JSON Report to the Web App to create a new online Report with all answers pre-filled/copied from the offline JSON file, and which can be modified further.
- **CC BY Reports**: browse Reports that have been made publically available on the platform
- **My Reports**: browse one's own public and private Reports
- **My Profile:** account settings
- **Help/About**: icon with a question mark, contains information on the ARTEM-IS project and simplified instructions that describe basic options in the Web App
- **Sign out:** icon with the door and arrow

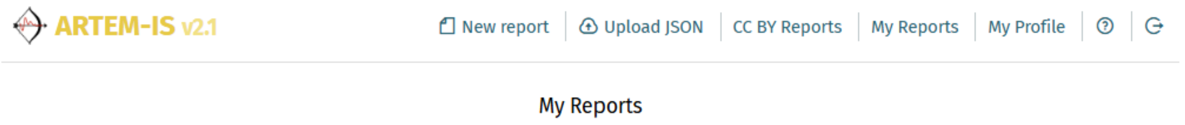

Figure 7. Main menu in the Web App

Adding a new ARTEM-IS Report and editing Reports

A new Report is created from the beginning by clicking on the option **New Report in** the main menu of the Web App. An existing Report can be edited or viewed by clicking on its title either from the **My Reports** or from the **CC-BY Reports** sections.

Any of these actions will open the screen for viewing or editing Reports, which can be seen in Figure 8 (ARTEM-IS Form). Each user can only edit Reports which they have created or to which they have been assigned as a Contributor, and they can view and download Reports that have been marked as publically available, but they will be unable to edit them.

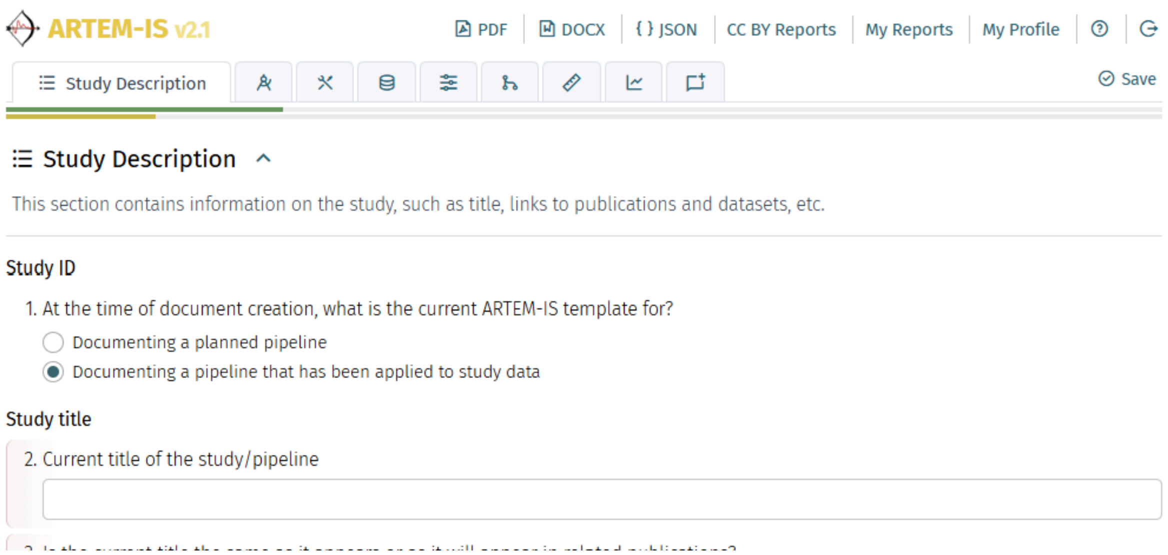

Figure 8. Editing a Report using ARTEM-IS Form

The Report viewing/editing page has a slightly different menu in the top right corner, with the following new options in addition to the already described ones:

- **PDF:** download a PDF version of this Report
- **DOCX**: download a DOCX version of this Report
- **JSON:** download a JSON version of this Report

Below the main menu, there is a bar which allows moving back and forth through different sections of the Report. Each section of the Report is shown in a separate tab, each represented with an icon (hovering over an icon reveals the name of each section).

To the right of all tabs is a **Save** button, which allows saving the progress. To make this easier, changes are saved automatically within 5 seconds as edits are made to a Report and when moving to a different section in the Form. The Save button icon shows a **tick symbol** if all progress has been successfully saved, and a **rotating circle** if uploading is still in progress. Moving on to a new tab or a different menu in the Web App before all the progress has been successfully saved prompts a warning to avoid any information loss.

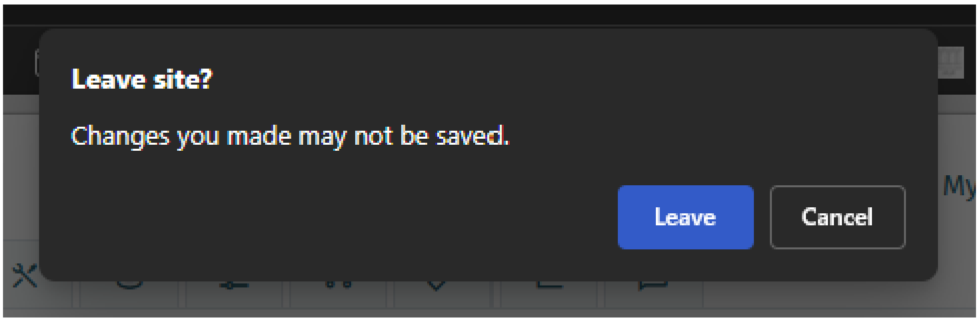

Figure 9. Warning when trying to leave a section before the progress has been saved

Below this menu, there are two **progress bars,** which visualise completion of the Report. The top bar shows the progress in filling in the whole Report, and the lower bar the progress

in filling in the currently open section. By clicking on a progress bar, you will be taken to the first unanswered item in the entire Report or the current section, allowing easier navigation.

After this, the Section title and brief introduction are shown. The introduction can be hidden by clicking on the arrow next to the section title, and it is followed by questions belonging to this section, divided into subsections. Items that are yet to be filled in are clearly marked with red boxes (see question 3 in Figure 8). The user can move between sections either by clicking on the button taking the user to the next section at the end of each page, or by clicking on the tab which they want to open. If a question has an **info logo**, the user can hover over it to get additional instructions on filling in the answer, such as additional instructions on appropriate response formatting or examples of possible answers. It is recommended to have read through these additional instructions when filling in a Report for the first time!

Removing mistakenly added answers. When answering each question, answers can be both added and removed. For multiple-choice questions, a mistakenly selected answer can be removed by clicking on the selected response again to unmark it.

As noted above, there are no mandatory questions to complete a report. Each Report can be saved, downloaded, and publically shared in any stage of the completion progress.

Creating, viewing the pre-processing pipeline

While filling in the questions on most pages is straightforward, describing the Preprocessing sections offers some additional options.

In the Pre-processing steps subsection, the user can:

- add new steps using a **drop-down menu**;
- use **arrows** on the right side to hide and show details for each individual pre-processing step, or use the arrows on the top to control visibility of details for all steps;
- edit details of each step in the same way as in all sections;
- hold and drag the **dots sign** to the left of each step to reorder the steps;
- add unique label for a step by clicking on the **pencil sign** (useful for navigating multiple instances of the same step, which is common with artifact removal) - the new label can be saved by pressing Enter or clicking on the **tick sign**, or the user can press Cancel to close the editing option without saving;
- remove a step using the **bin sign**.

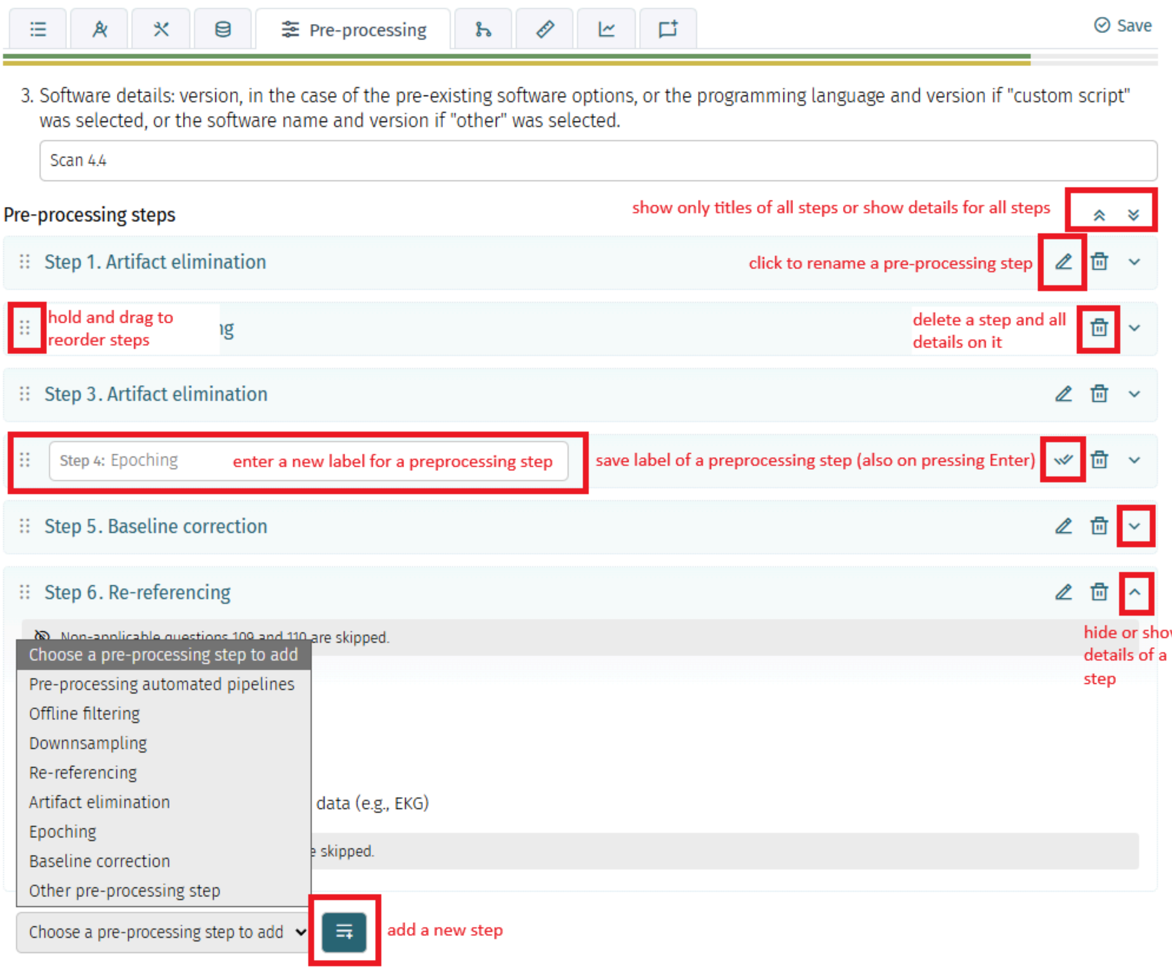

Figure 10. Pre-processing pipeline options

Browsing your own Reports and editing Report metadata

On the **My Reports** page, users can browse their own Reports (see Figure 9). The existing Reports are shown 15 at a time and the full list can be either browsed by navigating through pages of the reports on top of the list or by typing in part of the title in the search bar just above the top of the list.

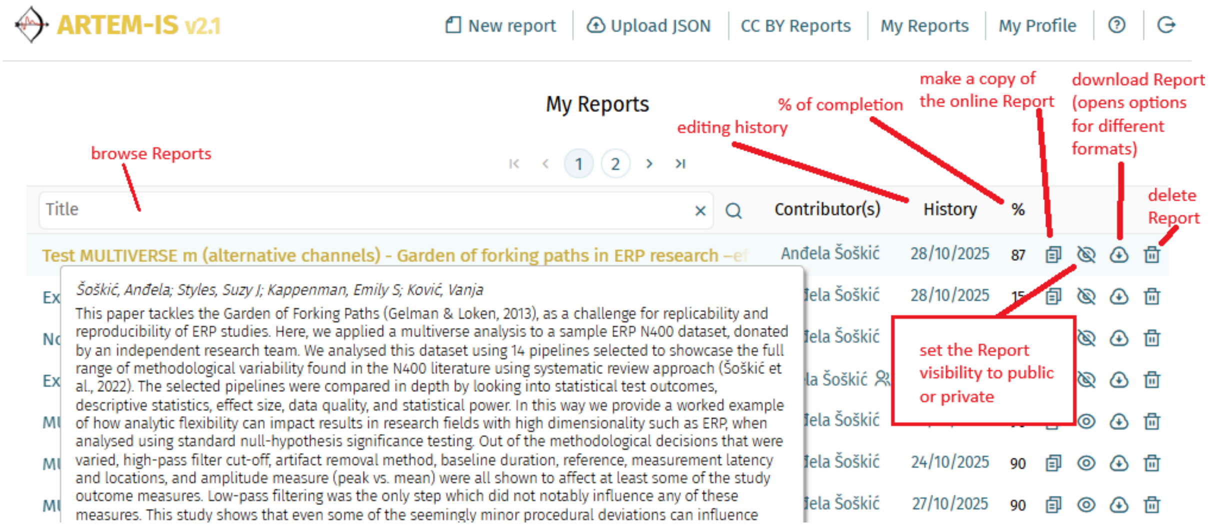

Figure 11. Browsing one's own Reports

Users can (looking at options from left to right):

- open the Report for viewing and editing by clicking on the title
- hover on top of the title of a Report to see the authors and the abstract of the study described in this Report;
- see and edit Contributors to a Report (see appropriate section for more information);
- see last date when the Report was edited in the History column and click on the last date to see a more detailed history of editing;
- see percent of completion of each Report by looking at the "%" column;
- set the Report to be public or private (see appropriate section for more information);
- download PDF, DOCX, or JSON Report by clicking on the download logo and choosing the appropriate option;
- delete a Report by clicking on the trash bin logo.

Setting up and viewing Report Contributors

Report contributors can be viewed from **My Reports** or **CC-BY Reports**, under the **Contributor(s)** column, where the name of the main Contributor (the document creator) is displayed (Figure 10). Hovering over the name reveals additional Contributors if there are any, with a logo of a group of people appearing next to the main Contributor name to draw attention when multiple people are listed.

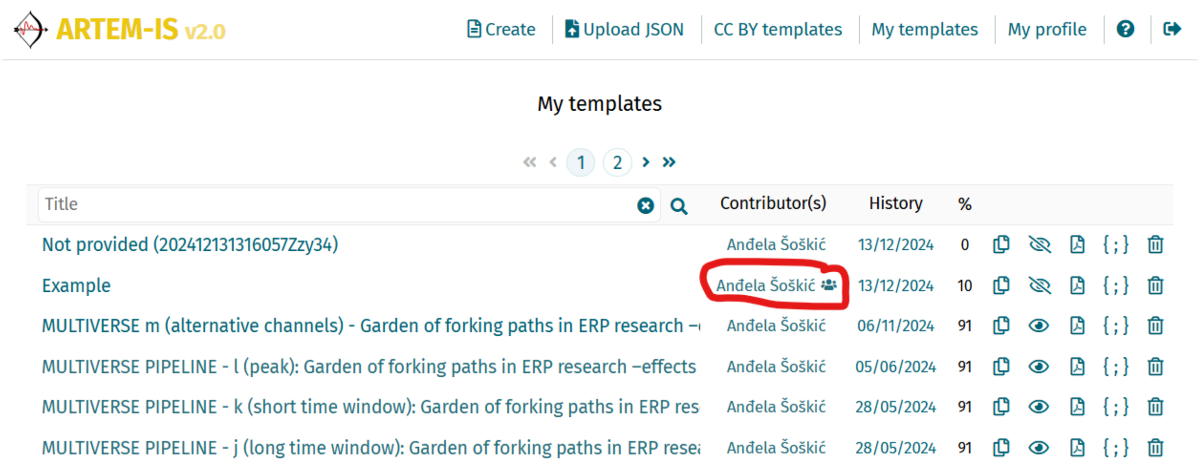

Figure 12. Field for viewing and editing Contributors

Only those already on the Contributor list can modify it—this includes adding, editing, or removing others—but no Contributor can remove themselves from the list. To edit or add Contributors, click on the **Contributor(s)** field (at the main Contributor's name): if you are listed as a contributor, this action opens a screen for editing (see Figure 11), including an option to add more Contributors. Add each new Contributor by typing in the email address tied to their account and clicking on the button to the right. Already assigned Contributors are listed below and you can remove each by clicking on X. If you are not a Contributor, a message stating *"You have no privilege to edit the list of contributors to this report"* will appear.

Information on authors of a study is separate from the information on the Contributors to its ARTEM-IS Report (the list of authors can be found inside the first section of a Report). This allows, for example, researchers to systematically document methods of papers that are published by others for the purposes of systematic reviews and meta-analyses.

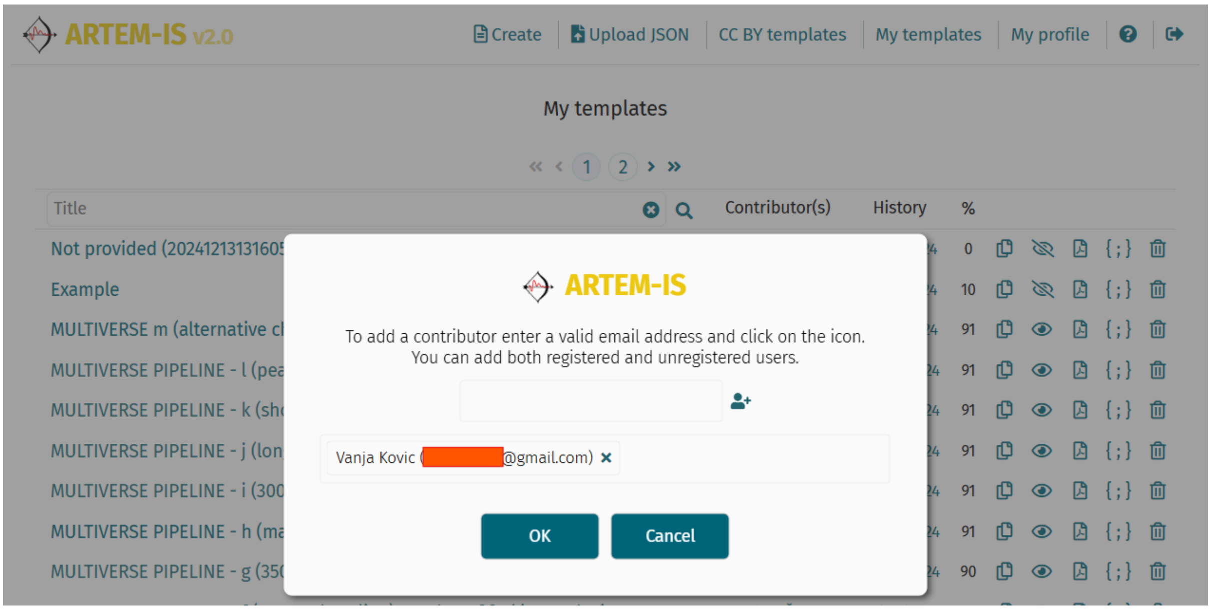

Figure 13. Adding or removing a Contributor

Private and public reports

Reports created by ARTEM-IS Web App users can be saved on the platform in two privacy modalities:

1. **Private Report -** only the users that have been assigned as Contributors to a private Report can see them as well as edit them, in the **My Reports Section**
2. **Public Report with CC-BY 4.0 licence** - if a user opts to make the Report public, the Report is shared with a CC-BY licence and it can be seen on the **CC-BY Reports** page. Only the Contributors to the Report can make edits, and anyone who is a registered user can view, copy, or download public Reports.[1]

Settings for private and public Reports can be viewed on the **My Reports** page, in the column with the eye icon. If the Report is private, the eye icon will be crossed out, while for public (CC-BY) Reports, the eye icon is not crossed out. Clicking on the eye icon toggles the Report's status between **Public** and **Private**. When a user decides to make a private report public, a reminder appears explaining the implications and asking for confirmation: *"This action will make your report public under the CC BY 4.0 licence. Other registered users will be able to see it and make copies, but not to edit it. You can revert this action at any time."* It is important to be cautious when changing the report's status; even if a report was public for

[1] At the moment, ARTEM-IS Web App does not allow sharing Reports under different types of public licenses, though it is a question whether other license types would make sense given the intended uses of the Reports. If this is reconsidered in the future, the ARTEM-IS team will need to consider how this affects permissions for sharing, copying, downloading and uploading Reports, and Web App developers will need to make necessary adjustments to the Web App backend code.

a very short time, there is still a risk that someone may have viewed or downloaded it in the interim.

Browsing and viewing public Reports

Browsing and viewing public reports is similar to **My Reports**, but simplified—there is no **visibility** button or **trash bin** icon. If a user opens a public Report where they are not listed as a **Contributor**, the Report will be frozen for editing. However, the user can still view the Report, download it as a PDF, DOCX, or JSON file, or make a copy of the online Report.

Downloading an existing Report

Reports can be downloaded from:

1. Report Overview (**My Reports** or **CC-BY Reports**) by clicking on the corresponding icon.
2. When a specific report is opened, by selecting **PDF** or **JSON** from the menu in the top-right corner.

The downloaded report can then be shared like any other file through private or public channels or stored in personal archives, for example, to use the static version as an attachment to scientific articles. Alternatively, a link to the online Report hosted on the ARTEM-IS platform can be attached as part of a scientific paper, such as in the Methodology section. However, it is important to note that the online version remains open to further edits, and previous versions cannot be accessed (though the edit history provides information on whether and when the report was modified after the paper's publication).

Using an existing Report as a template for a new one

Let's start with an example—laboratories may want to save *hardware settings* in one place and keep a pre-filled template with the common settings that only needs to be supplemented with offline processing and later steps. Similarly, many laboratories use *standard designs and pipelines*. They can keep records of standard methods to ensure consistency between studies by creating pre-filled reports with these standardized settings, which will also save them significant time when creating ARTEM-IS Reports on these studies. . A third use case is the *replication or continuation of a study*—if a study already has a publicly available ARTEM-IS Report, authors of the replication or follow-up study can copy the existing Report and modify it where deviations occur. Finally, for pre-registered studies, researchers can make a copy of the Report containing the pre-registered plan when creating the Report on the completed study, and easily change only the *deviations between pre-registration and what was eventually done*.

There are two ways to create a copy:

1. *Copy the online report directly* without needing to download and upload it—this is available for both your own reports and public reports. The option to **Copy** (copy icon) is located in the report overview under **My Reports** or **CC-BY Reports**.

2. If a report has been downloaded in JSON format, it can be *uploaded back to the online system.* This can even be done with JSON files of private reports whose online versions are not visible on the ARTEM-IS platform (e.g., when an author downloads and uploads their own Reports or shares them with someone else via email). *Use case scenario:* a lab manager can privately share a half-filled report containing hardware settings with lab members, who can upload it and use the pre-prepared reports to describe all studies conducted with those settings more efficiently.
    - The option to **Upload JSON** is available in the top-right menu of the **My Reports**or **CC-BY Reports** pages.

If a user uploads an older, V2.0, version of the JSON Report, they will be notified that the Report has been converted to V2.1. The user will also be prompted to check and adjust the order of pre-processing steps, an option that was not available in V2.0.

In both cases—whether uploading or copying—the result is a *new online Report* with all answers pre-filled/copied from the original report. The new Report will have a new `Report ID`, distinct from the original report that was copied. The newly created online Report can also have a completely different team of Contributors compared to the original. The main contributor for the new Report is the user who uploaded the offline document or created the copy.

The new Report will be clearly marked as a *derivative* of the original report, with a link to the original source (while ensuring privacy protections for private originals), which will be further explained in the next section.

Tracking down the origin of a Report

As explained in the previous section, if a Report was created by copying another report, it is clearly marked as a derivative of the original report:

1. In the online view within the Web App, a clickable link to the original is displayed just below the progress bar (see Figure 12).

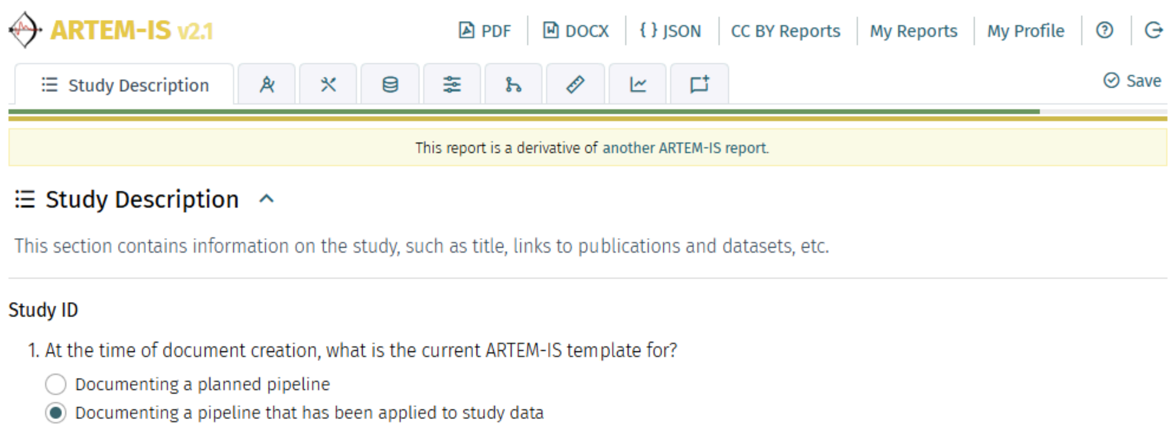

Figure 14. Link to the source Report for derivative Reports in the online From

2. In the PDF and DOCX Reports, there is a clickable link to the original Report at the end of the PDF document, together with a note stating *"**Derivative.** This report is a*

derivative of [Report Title] by [Contributors]. [link to the original Report that has been used]" is provided at the end of the Report).
3. In the JSON Report, the origin is identified by the `Source ID` in the Report metadata section (object) at the beginning of the Report.

Clicking on the link to the original opens the source Report in its online version on the ARTEM-IS platform. If the user has access to the source Report—either as a **Contributor** or because the report is **public**—they will be able to view it. If the source Report itself is a derivative of another Report, the same process can be followed to trace the chain back to the first Report from which the chain originated.

If a source Report has been deleted in the meantime, the user will not be able to access it and will instead receive a notification stating that the report has been deleted. Similarly, if the source Report is private and the user does not have access, they will see a notification indicating that this Report is private.

Help/About

The **Help/About** section offers a brief overview of key user tips, providing a condensed version of the information outlined in this document. It also includes direct links to the project's pages on **OSF** and **GitHub** for further resources. Additionally, the section features an **Update History**, where users can click on any listed update to automatically view a detailed report of changes, as described in the *Keeping Track of Changes* section.

Reading JSON Reports

Structure of a JSON Report

Intro section (JSON object): In its first, `general_information`, section (i.e., JSON object), a Report in the JSON format contains meta-information about the specific Report itself: ARTEM-IS version, the licence under which the report was available (private vs. CC-BY public) when downloaded, together with the date on which the Report has been downloaded, unique ID of the Report (`Report ID`), percent of completion (`Completed`). If a Report has been created by copying another Report, the unique ID of the original Report that was used as the template will be provided in the field `Source ID`.

Template sections: This section is followed by separate sections (i.e., JSON objects) that represent each of the major sections in the ARTEM-IS form. Each section contains its reporting items.

Reports in JSON format feature item codes rather than full question text of the items, as the item codes for the same question remain unchanged between versions of ARTEM-IS, even if minor details change, for example in the case of a slight rewording of a question to make it more clear. Item codes for each question can be found in the ARTEM-IS Template Specification, inside the ARTEM-IS Spreadsheet, in the column `item_pref_label`.

Equally, for multiple-choice questions, response codes are given rather than full-text versions of responses. Response codes can be found in the ARTEM-IS Template Specification, inside the ARTEM-IS for ERP v2.1 Presets table, in the column `value`.

Representation of subsections:

Subsections are not represented as such in the JSON Report, with the exception of the preprocessing steps in the Preprocessing section, because this section has the option to add desired preprocessing steps and to have multiple instances of each.

The preprocessing steps are all nested inside a JSON object named `pre-processing steps`, within the Preprocessing section's JSON object. Inside this object, there is a separate JSON object for each step. Each object representing the steps has a unique 6-character key (object name) in line with the following convention:

- The first three letters are a code for the type of preprocessing operation: `art` for artifacts, `off` for offline filters, `epo` for epoching, `bas` for baseline correction, `rrf` for rereferencing, `api` for automatic preprocessing pipelines, `dwn` for downsampling, and `oth` for other, self-described operations.
- The latter three characters code multiple instances of the same preprocessing step and code the order in which they were created. For example, the artifact removal step which was created first will be named `art001`, the next one `art002`, and so on. Reordering the steps does not change their object names.

The pre-processing step objects contain step order in the pipeline (`order`), type of the pre-processing step among the offered options (`type`), a self-described title of the pre-processing step (`title`), and the response items related to this preprocessing step nested inside a JSON object called `details`.

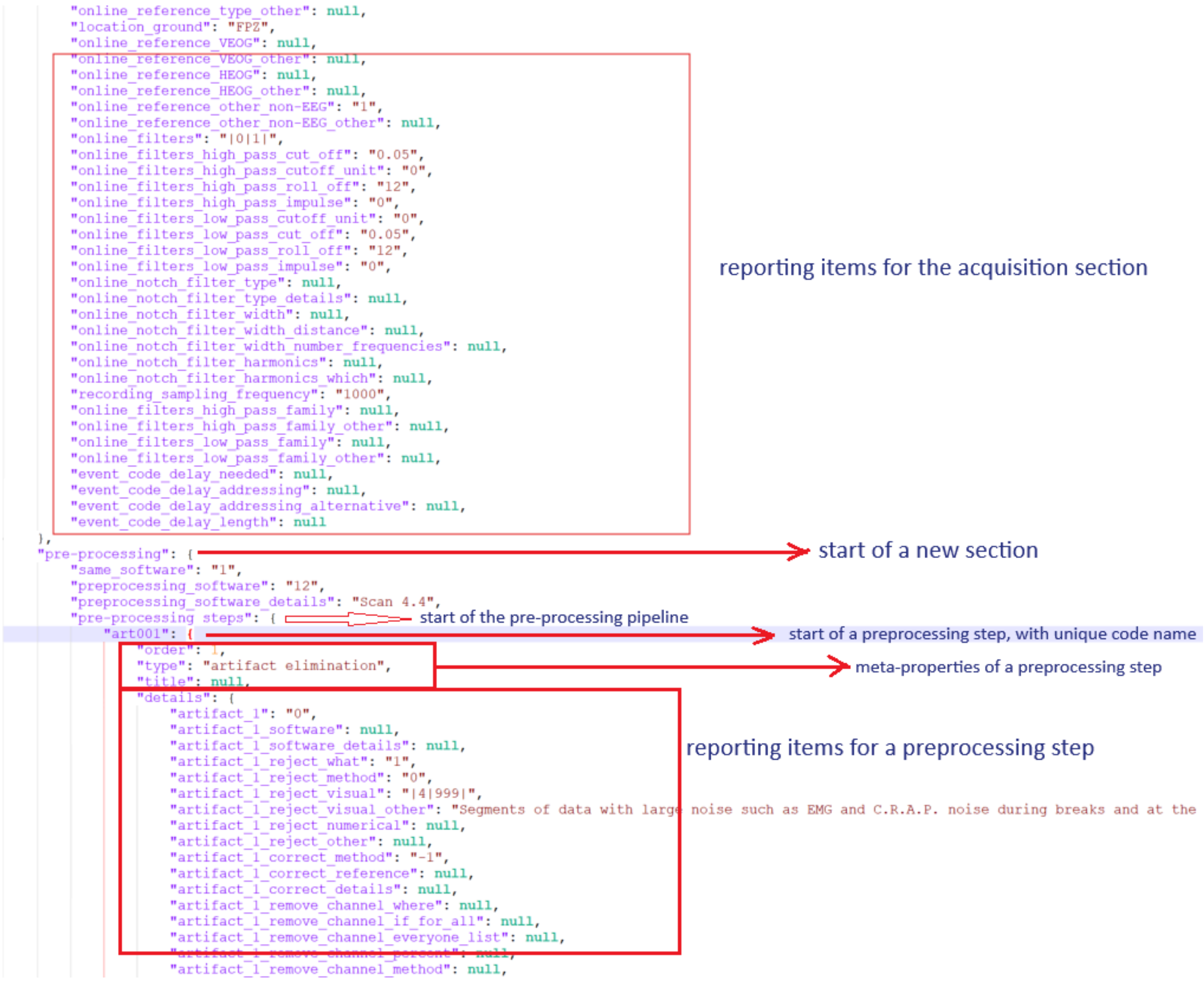

Figure 2. Example ARTEM-IS for ERP v2.1 Report in JSON format.

ARTEM-IS for ERP v2.1 Template Specification

An ARTEM-IS Template is defined using two data sheets, described below, which define questions, rules for their dynamic showing and hiding, the appropriate type of answer to each question, and all other properties of the Template. The two spreadsheets jointly constitute an **ARTEM-IS Template Specification**. In order to read JSON Reports, the user will need to find question and response codes within the Template Specification, so they will be described here briefly.

The first spreadsheet, called simply **ARTEM-IS Spreadsheet**, contains all questions that can be found in a Form/Template and specifies their properties. For ARTEM-IS for ERP v2.1, this table is called ***ARTEM-IS for ERP 2.1 Spreadsheet***. The table contains separate tabs for each section of the Template (visible as a separate tab in the online Form), to make the spreadsheet easier to navigate (see Table 1):

Table 1. Tabs in an ARTEM-IS for ERP v2.1 Spreadsheet.

Tab name in the Spreadsheet	Corresponding tab in the Web App
`study_id`	Study Description

experimental_design_sample	Design and Sample
hardware	Hardware
acquisition	Acquisition
preprocessing	Pre-processing
channels	Channels
measurements	Measurements
visualization	Visualization
other	Other

Within each tab, the rows represent questions that are found within the given section of the Template. Columns in the tables contain the following properties of these sections (see Table 2). The columns the most relevant to reading JSON Reports are highlighted in yellow.

Table 2. Columns within each tab in the ARTEM-IS for ERP v2.1 Spreadsheet.

**Column name in the Spreadsheet**	**What does the column define**
sub_section	Subsection within the Form/Template section to which a given question belongs. Visible in online and PDF Reports.
preamble	Contents of the brief description on top of each tab in the Web App that introduce a given section (e.g., "This section contains information on the study, such as title, links to publications and datasets, etc.")
question	Item question, as it appears on the screen of the Web App and in the PDF report
details	Additional instructions for the user on how to answer a given question that show up when a user hovers over the question mark next to a question
field_type	Type of question (e.g., multiple-choice).
choices	Defines which choices will be offered in multiple-choice questions (radio, radio_multiple, select, select_multiple types).
item_pref_label	Variable name and simultaneously a unique ID of each question<br><br>This is the title for each question in the JSON report
visibility	Logical expression that determines whether the item should be displayed or not.
item_order	order in which a given item appears within a section, as shown on the Web App screen and in PDF Reports

`include`	whether a question should be included in the Template/Form or not
`mandatory`	This defines whether it is mandatory to answer a question in order to complete the Form.
`activity_pref_label`	This is the variable name of the section that a question belongs to in the backend of the Web App. In ARTEM-IS JSON reports, `activity_pref_label` acts as the name of each section.
`activity_order`	Place of the section that a question belongs to within the order of all sections.<br><br>For pre-processing steps, which can be added, reordered, and removed, this column also contains a unique letter code for each pre-processing step (e.g., 5a, 5b).
`unit`	Physical unit in which a numerical value of a physical measure should be entered (e.g., Hz, ms, dB/octave).
`BIDS_file`, `BIDS_key`, `BIDS_key_for_unit`, `BIDS_status`	Fields that help keep track of correspondences between ARTEM-IS and BIDS to help maintain consistency of terminology and allow potential future integrations, to allow, for example, import of information directly from BIDS-compliant datasets
`Change_log`	History of changes to a given question.

In addition to this spreadsheet, there is a spreadsheet that defines answers that should be offered to multiple-choice questions. These are defined in a separate spreadsheet because there are multiple instances when different questions have the same answer options (an obvious example are yes-or-no questions), and in these cases only one unique response set is used by multiple different questions. For ARTEM-IS for ERP v2.1 this table is called ***ARTEM-IS for ERP 2.1 Presets***.[2]

This spreadsheet has one tab, in which all response sets are defined. Each unique response set for a multiple-choice question is represented by as many neighbouring rows as there are choices (options), and the response set name (column `type`) defines which answer options belong to the same response set.

> For example, the response set for the yes-or-no question is called “boolean” and it has two options, “yes” and “no”. In the ARTEM-IS for ERP 2.1 Presets spreadsheet, it is represented by two rows, one for each option. Both answer options have the same

[2] Why call it Presets? The name is borrowed from eCOBIDAS spreadsheet format, which we used when creating ARTEM-IS v1.0 Spreadsheet, and which we largely based ARTEM-IS v2.1 Spreadsheet on. In eCOBIDAS, most response sets are defined within the main spreadsheet together with the questions, while some response sets are defined in their own spreadsheets, usually those that are used often by different questions or those that include a lot of options. In eCOBIDAS terminology, the response set variables defined by these separate spreadsheets are called Preset Variables. We moved away from this format for ARTEM-IS v2.1 and opted to have one spreadsheet containing all response sets to allow better version control and easier oversight, but tried to keep as much consistency to foster exchange and collaboration between ARTEM-IS and eCOBIDAS teams.

response set name (`type` column) to indicate that they belong to the same response set (see Figure 13).

1	id	type	name	value	newvalue	sort	active	Last id: 1391
65	1064	boolean	no	0	0	0	1	
66	1065	boolean	yes	1	1	1	1	

Figure 13. Representation of response sets for multiple-choice questions in an ARTEM-IS Presets spreadsheet.

Therefore, each row is one choice within a response set. Notably, different response sets may offer the same answer option (for example, many different questions allow the user to select “other”). In such cases, each instance of the choice that appears multiple times is treated as a separate unique response option, and it has its own row within the group of rows representing a given response set.

Columns in the ARTEM-IS for ERP 2.1 Presets table are described in Table 3. The columns the most relevant to reading JSON Reports are highlighted in yellow.

Table 3. ARTEM-IS for ERP 2.1 Presets columns

**Column name in the Presets spreadsheet**	**What does the column define**
`id`	Unique ID of each response option
`type`	Unique name of the response set to which this answer option belongs.
`name`	text of the answer option, as it appears on the screen of the Web App and on the PDF report (e.g., “yes”, “no”)
`value`	ID code of a response option *within its response set*. These are shown as response values in JSON Reports.
`sort`	order in which options appear on the screen within a response set
`active`	Takes value 1 if the response option is currently in use, and value 0 if the response option has been discontinued.
`change_log`	History of changes to a given item.
`Last ID`	There are no values in this column, this is just a counter that shows the largest ID value.

Links to ARTEM-IS Template Specification spreadsheets

Table 4. Links to ARTEM-IS Template Specification Spreadsheets

**Which form**	**Spreadsheet**	**Version**	**Link**

ARTEM-IS for ERP	Question settings	work-in-progress, Google Spreadsheet: v2.1	https://docs.google.com/spreadsheets/u/0/d/10Mn0MvrEMRqSv7JYWJkwFBWAprww2SL9ozaIAAqH0Qs/edit
		static version of v2.0	https://osf.io/3ga5e
		static version of v2.1	https://osf.io/vyuqm
	Question settings *with response options*	static version of v1.0	https://osf.io/9dweq
	Response options for multiple-choice questions	work-in-progress, Google Spreadsheet: v2.1	https://docs.google.com/spreadsheets/u/0/d/1cXUaA6CpuXN_w9OBVMz7DXH0smpqUycI8vUhS9fYFms/edit
		static version of v2.0	https://osf.io/wj94q
		static version of v2.1	https://osf.io/8nuae

Keeping track of changes between Template versions

There are two ways to keep track of changes between two versions of an ARTEM-IS for ERP Template.

Firstly, ARTEM-IS Web App keeps an automatically generated changelog which lists all changes between the previous version and the latest one. Automatically generated changelogs for each version can be found in the Web App itself (on this page: https://artemis.incf.org/help) and a permanent copy of them can be found on the OSF (https://osf.io/hcmgt).

In addition, ARTEM-IS Specification spreadsheets both include a Change_log column in which change to each field is documented and explained briefly, starting from updates from v1.0 to v2.0 (this is a new addition to ARTEM-IS v2.0). This column is easier for a typical reader, includes a rationale and centralises all changes to the item within one update, as well as from different updates (conversely, automatic change log groups each type of change together, so as a result changes to response option text and adding new response options in the same question are in separate sections of the report).

Citing ARTEM-IS

If you used the ARTEM-IS for ERP Web App for your research, if you wish to refer to ARTEM-IS for ERP Template, or if you wish to cite information to this document, please cite the following publication, to which this document is a supplement:

> Šoškić, A., Kovic, V., Algermissen, J., Fischer, N. L., Ganis, G., Gau, R., … Styles, S. J. (2023, January 5). *ARTEM-IS for ERP: Agreed Reporting Template for EEG Methodology - International Standard for documenting studies on Event-Related Potentials.* https://doi.org/10.31234/osf.io/mq5sy

If you wish to refer to the general ARTEM-IS concept or project, the design principles behind the project, or the ARTEM-IS Statement, please cite the following publication:

> Styles, S. J., Ković, V., Ke, H., & Šoškić, A. (2021). Towards ARTEM-IS: Design guidelines for evidence-based EEG methodology reporting tools. *NeuroImage, 245,* 118721. https://doi.org/10.1016/j.neuroimage.2021.118721

Licence

The **ARTEM-IS for ERP Template** is licensed with a Creative Commons Licence (CC) with an obligation for attribution (BY), for non-commercial uses (NC), and with the obligation that others will also share their resulting work with an equivalent licence (SA) (i.e., CC-BY-NC-SA). In addition, we allow educational uses such as in higher education or commercial training courses.

The **ARTEM-IS Web Application** is currently not open source, but we are committed to making it open in due course. Please note that the ARTEM-IS Web App is intended to be a tool that facilitates creating and sharing ARTEM-IS Reports. As such, it derives all of the essential functionality directly from the ARTEM-IS Template Specifications, which are openly available as stated above, and which can be used to create and share ARTEM-IS-compliant reports independently of the Web App. The only intellectual property that is not shared is the code for displaying the user interface and managing reports in the backend of the ARTEM-IS official Web Application on the International Neuroinformatics Coordinating Facility (INCF) platform (https://artemis.incf.org/).

ARTEM-IS Extensions

Here are some examples how the ARTEM-IS Template Specification can be used in the future, while adhering to its licence (see Licence):

(1) **Translation of ARTEM-IS for ERP into other languages.**
If you are interested in creating an official translation into another language that we would endorse, link to, or possibly make available on our Web App, please get in touch.
You can, of course, also create and host independent and unofficial translations on

your own platform, which will be made easier once the Web Application code is open source, too.

(2) **Develop your own ARTEM-IS Templates or ARTEM-IS inspired Templates, as well as extensions and upgrades to ARTEM-IS for ERP.**
If you want to join the ARTEM-IS Working Group and develop future ARTEM-IS Templates for EEG or to expand ARTEM-IS beyond EEG, please get in touch.
In addition, you can create new reporting tools inspired by ARTEM-IS. In the latter case, the resulting templates should not be called ARTEM-IS Templates to avoid confusion. The ARTEM-IS source should be appropriately credited.

(3) **Make your own ARTEM-IS for ERP Web Application** - for example, an internal application for your laboratory projects or for use in education or training. We hope to facilitate this by making the code openly available in the future.

Contact

If you notice a bug or have a suggestion, please post an issue on GitHub.

If you want to join the ARTEM-IS team, reach out to one of the ICNF Working Group chairs.

If you have general questions, or are interested in collaboration, you are welcome to use the same contact route, too.

References

Keil, A., Debener, S., Gratton, G., Junghöfer, M., Kappenman, E. S., Luck, S. J., Luu, P., Miller, G. A., & Yee, C. M. (2014). Committee report: Publication guidelines and recommendations for studies using electroencephalography and magnetoencephalography. *Psychophysiology*, *51*(1), 1–21. https://doi.org/10.1111/psyp.12147

Pernet, C., Garrido, M. I., Gramfort, A., Maurits, N., Michel, C. M., Pang, E., Salmelin, R., Schoffelen, J. M., Valdes-Sosa, P. A., & Puce, A. (2020). Issues and recommendations from the OHBM COBIDAS MEEG committee for reproducible EEG and MEG research. *Nature Neuroscience*, *23*(12), 1473–1483. https://doi.org/10.1038/s41593-020-00709-0

Styles, S. J., Ković, V., Ke, H., & Šoškić, A. (2021). Towards ARTEM-IS: Design guidelines for evidence-based EEG methodology reporting tools. *NeuroImage, 245,* 118721. https://doi.org/10.1016/j.neuroimage.2021.118721

Šoškić, A., Ković, V., Algermissen, J., Fischer, N. L., Ganis, G., Gau, R., … Styles, S. J. (2023, January 5). *ARTEM-IS for ERP: Agreed Reporting Template for EEG Methodology - International Standard for documenting studies on Event-Related Potentials.* https://doi.org/10.31234/osf.io/mq5sy
