# Supplementary material for: ARTEM‐IS for ERP: Agreed Reporting Template for EEG Methodology—International Standard for Event‐Related Potential Experiments
Source: Psychophysiology. 2025 Dec 8;62(12):e70187. doi: 10.1111/psyp.70187 (PMC12683983; doi:10.1111/psyp.70187)
Supplement: Supplementary file 4 — Data S4: psyp70187‐sup‐0004‐Supinfo4.pdf. [file PSYP-62-e70187-s005.pdf]

ARTEM-IS for ERP v2.1 Report

This report is 40% completed.

1. Study Description

Study ID

1. At the time of document creation, what is the current ARTEM-IS template for?

> *Answer not provided*

Study title

2. Current title of the study/pipeline

> *University of Belgrade LNPK laboratory template with standard hardware and acquisition settings for Neuroscan*

3. Is the current title the same as it appears or as it will appear in related publications?

> *Answer not provided*

4. Has the current study or pipeline been known by a different (published or working) title?

> *Answer not provided*

Non-applicable question 5 is skipped over.

Authors

6. Authors of the study in the following format: family-name, other names. Each author separated by a semicolon and a space.

> *Answer not provided*

Publications

7. Has the study resulted in any published articles, conference presentations or research reports at this stage?

> *Answer not provided*

Non-applicable questions 8 to 11 are skipped over.

Datasets

12. Has this study resulted in any datasets archived in a persistent, version controlled repository, administered independently from the authorship team?

> *Answer not provided*

Non-applicable questions 13 to 18 are skipped over.

Supplemental materials

19. Has this study resulted in any supplementary materials other than data and publications?

> *Answer not provided*

Non-applicable questions 20 to 22 are skipped over.

Licenses

23. Are any elements in the study currently covered by a license allowing for reuse?

> *Answer not provided*

Non-applicable questions 24 to 31 are skipped over.

Study description

32. Study abstract

> *These are Neuroscan equipment settings which are standardly used by the Laboratory for Neurocognition and Applied Cognition, University of Belgrade. By copying this template rather than starting anew, members of the laboratory can save time because they do not have to fill in the information that is common for all studies, or they can refer to this template to check the default settings if they are unsure about them.*

33. Keywords, separated by semicolons followed by space

> *Answer not provided*

Funding

34. Does the project have (or did it have) financial support from a funding agency?

> *Answer not provided*

Non-applicable question 35 is skipped over.

Ethical Approval

36. At the time of writing has the study been approved by an ethics committee?

> *Answer not provided*

Non-applicable question 37 is skipped over.

Acknowledgements

38. Do you have any acknowledgments to add in addition to authors or funding?

> *Answer not provided*

Non-applicable question 39 is skipped over.

How to cite

40. Do you have any instructions on how you (or the authors) want this study to be cited or otherwise acknowledged?

> *Answer not provided*

Non-applicable question 41 is skipped over.

2. Design and Sample

Sample

1. How many participants took (or will take) part in the study?

> *Answer not provided*

2. How many participants, in total, were included in the analysis, after exclusion of participants?

> *Answer not provided*

Non-applicable question 3 is skipped over.

4. Age range of the sample: age of the youngest participant

> *Answer not provided years*

5. Age range of the sample: age of the oldest participant

> *Answer not provided years*

6. Does the sample include clinical population?

> *Answer not provided*

Variables

7. Is the design of this study full factorial?

> *Answer not provided*

8. How many between-subject factors does this study/pipeline have?

> *Answer not provided*

Non-applicable question 9 is skipped over.

10. How many experimental/comparison groups does the study feature in total?

> *Answer not provided*

Non-applicable questions 11 and 12 are skipped over.

13. How many within-subject factors does this study/pipeline have (excluding EEG factors)?

> *Answer not provided*

Non-applicable question 14 is skipped over.

15. How many within-subject conditions does the study have in total?

> *Answer not provided*

Non-applicable question 16 is skipped over.

17. Does the study include an analysis of a relationship between ERPs and a non-ERP continuous variable (e.g., correlation of an ERP measure with IQ)

> *Answer not provided*

Non-applicable question 18 is skipped over.

Presented trials

19. What determines the number of trials each participant was presented with?

> *Answer not provided*

Non-applicable questions 20 to 28 are skipped over.

Analyzed trials

29. After eliminating trials on all bases (e.g., artifacts, behavioural errors), how many trials, on average, did you include for each condition per participant?

> *Answer not provided*

30. The minimum number of trials analyzed per condition for any participant.

> *Answer not provided*

31. The maximum number of trials analyzed per condition for any participant.

> *Answer not provided*

Presenting software

32. Presenting software for the experimental task

> *Answer not provided*

33. Software details: version, in the case of the pre-existing software options, or the programming language and version if "custom script" was selected, or the software name and version if "other" was selected.

> *Answer not provided*

3. Hardware

Preamp peripherals

1. How were the EEG electrodes placed (or will be placed)?

> *cap or net*

Non-applicable question 2 is skipped over.

3. Cap or net make (brand)

> *Easycap standard caps*

Non-applicable question 4 is skipped over.

5. Cap or net model

> *Easycap*

6. Make (brand) of the electrodes

> *Easycap GmbH*

7. Model of electrodes

> *10-20*

8. Do the electrodes have pre-amplifiers?

> *no (passive electrodes)*

9. The material the electrodes are made of

> *tin*

Non-applicable question 10 is skipped over.

11. Type of conductive medium used for electrodes

> *conductive gel*

Non-applicable question 12 is skipped over.

Electrode positioning

13. Did you (or will you) use all electrode slots available in the cap (i.e., they had electrodes in them)?

> *Answer not provided*

14. What type of placement convention is appropriate for describing electrode placement scheme in this study?

> *International 10-20 system and its extensions (e.g., 10-10, 5 percent)*

15. Which extension of the 10-20 system will you use to describe electrode positions?

> *Answer not provided*

Non-applicable question 16 is skipped over.

17. Number of EEG electrodes used for acquisition (including, if they are on scalp, ground and reference electrodes, as well as earlobes; excluding standalone EOG and other non-EEG electrodes).

Answer not provided

18. The appropriate methods description should also include a list or a scheme of all electrode locations. A convenient way to do this is currently not available in ARTEM-IS due to technical limitations, so please provide either a list of all electrodes, or a link where the sceheme/list can be found.

Answer not provided

19. Did you (or will you) use any electrodes other than scalp electrodes and mastoids/earlobes?

Answer not provided

Non-applicable questions 20 and 21 are skipped over.

Signal Amplifiers

22. Make (brand) of the amplifier

Neuroscan (e.g., SynAmps RT, NuAmps)

Non-applicable question 23 is skipped over.

24. Model of amplifiers

NuAmps

Triggers

25. Source of triggers

presentation software trigger

Non-applicable question 26 is skipped over.

27. Do you wish to describe triggers in more detail?

yes

28. Were triggers delivered simultaneously with data acquisition (ow will they be)?

Online, during acquisition

29. Trigger input destination

Acquisition computer

30. Trigger source

Analogue (e.g., via a parallel port, serial port, or TCP/IP)

Other peripheral devices

31. Other equipment used during acquisition

Answer not provided

Non-applicable question 32 is skipped over.

4. Acquisition

Acquisition software

1. Acquisition software

Neuroscan

2. Software details: version, in the case of the pre-existing software options, or the software name and version if "other" was selected.

Scan 4.4

Data quality

3. Did you (or do you plan to) document impedances, or an alternative data aquisition quality measure?

Answer not provided

Non-applicable questions 4 to 8 are skipped over.

Online (recording) reference

9. Was voltage measured, or will it be measured, relative to an online reference for EEG electrodes (as opposed to alternative solutions such as Driven Right Leg (DRL))?

yes

10. Location of the EEG online reference electrode(s)

Answer not provided

Non-applicable questions 11 and 12 are skipped over.

13. Location of the ground electrode during recording

AFz

14. Online reference for VEOG electrodes

Answer not provided

Non-applicable question 15 is skipped over.

16. Online reference for HEOG electrodes

Answer not provided

Non-applicable question 17 is skipped over.

18. Online reference for other non-EEG (and non-EOG) electrodes

the same reference as EEG electrodes

Non-applicable question 19 is skipped over.

Online filters

20. Which of the following online filters were used, or will be used?

high pass, low pass

21. High-pass filter cut-off used during recording

0.05 Hz

22. Type of online high-pass filter cut-off

half-amplitude

23. High-pass filter roll off used during recording

12 dB/octave

24. High-pass filter impulse response during recording

finite impulse response (FIR)

25. High-pass filter family during recording

Answer not provided

Non-applicable question 26 is skipped over.

27. Type of online low-pass filter cut-off

> *half-amplitude*

28. Low-pass filter cut-off used during recording

> *200 Hz*

29. Low-pass filter roll off used during recording

> *12 dB/octave*

30. Low-pass filter impulse response during recording

> *finite impulse response (FIR)*

31. Low-pass filter family during recording

> *Answer not provided*

Non-applicable questions 32 to 39 are skipped over.

Sampling

40. Acquisition sampling rate

> *1000 Hz*

Event code delay

41. Did you (or will you) need to account for the event code delay?

> *Answer not provided*

Non-applicable questions 42 to 44 are skipped over.

5. Pre-processing

Pre-processing general

1. Is the same software used for all pre-processing steps?

> *Answer not provided*

Non-applicable questions 2 and 3 are skipped over.

Order of operations

4. Do you want to additionally self-describe the order of preprocessing operations?

> *Answer not provided*

Non-applicable question 5 is skipped over.

6. Channels

Selection of channels for analysis

1. Which channels were (or will be) analyzed?

> *a subset of channels submitted to statistical analysis*

2. Rationale for selecting this exact subset of channels

> *Answer not provided*

Non-applicable questions 3 to 28 are skipped over.

Channel aggregating

29. Are channels used individually or aggregated into regions of interest when doing component measurement and/or statistical analysis

> *Answer not provided*

Non-applicable questions 30 to 32 are skipped over.

7. Measurements

Measurement

1. Does this study include measuring amplitude and/or latency for the purposes of later statistical analysis?

> *Answer not provided*

Non-applicable questions 2 to 81 are skipped over.

8. Visualization

Plot type

1. How were ERPs visualised (or how will they be visualised)? Hover over the help sign for the visualisation naming convention.

> *Answer not provided*

Non-applicable questions 2 to 76 are skipped over.

9. Other

Additional comments

1. Do you have any additional information or comments to add?

> *Answer not provided*

Non-applicable question 2 is skipped over.

Contributor(s)

Anđela Šoškić,Vanja Kovic

License

This report has been downloaded under the CC BY 4.0 license on October 28, 2025.
https://artemis.incf.org/20230825143927kgJzf5
