# Supplementary material for: ARTEM‐IS for ERP: Agreed Reporting Template for EEG Methodology—International Standard for Event‐Related Potential Experiments
Source: Psychophysiology. 2025 Dec 8;62(12):e70187. doi: 10.1111/psyp.70187 (PMC12683983; doi:10.1111/psyp.70187)
Supplement: Supplementary file 6 — Data S6: psyp70187‐sup‐0006‐Supinfo6.pdf. [file PSYP-62-e70187-s001.pdf]

ARTEM-IS for ERP v2.1 Report

This report is 98% completed.

1. Study Description

Study ID

1. At the time of document creation, what is the current ARTEM-IS template for?

> *Documenting a pipeline that has been applied to study data*

Study title

2. Current title of the study/pipeline

> *Anterior N2 enhancement is not a general electrophysiological index of concealed information*

3. Is the current title the same as it appears or as it will appear in related publications?

> *yes*

4. Has the current study or pipeline been known by a different (published or working) title?

> *no*

Non-applicable question 5 is skipped over.

Authors

6. Authors of the study in the following format: family-name, other names. Each author separated by a semicolon and a space.

> *CW Hsu; T Schofield; G Ganis*

Publications

7. Has the study resulted in any published articles, conference presentations or research reports at this stage?

> *yes*

8. Do any of these publications have a DOI?

> *yes*

9. DOIs of related publications, separated by a semicolon and a space.

> *https://doi.org/10.1111/psyp.14633*

10. Are there any web-accessible publications that do not have a DOI?

> *no*

Non-applicable question 11 is skipped over.

Datasets

12. Has this study resulted in any datasets archived in a persistent, version controlled repository, administered independently from the authorship team?

> *no*

Non-applicable questions 13 to 18 are skipped over.

Supplemental materials

19. Has this study resulted in any supplementary materials other than data and publications?

no

Non-applicable questions 20 to 22 are skipped over.

Licenses

23. Are any elements in the study currently covered by a license allowing for reuse?

no

Non-applicable questions 24 to 31 are skipped over.

Study description

32. Study abstract

Event-related potentials (ERPs) have been used with the concealed information test (CIT) to detect concealed recognition of specific stimuli (i.e., "probes"). While most research has focused on the P300 component, which is larger for infrequent probes than for frequent control stimuli (i.e., "irrelevants"), some studies have investigated an earlier ERP component, the anterior N2, with mixed results. Although some studies have reported a larger anterior N2 for probes than irrelevants (N2 enhancement), other studies, including our own, have not found such an effect. The present study aimed to replicate and extend our previous findings using the same CIT paradigm and measurement parameters. Results of Bayesian analyses show strong evidence against the hypothesis of anterior N2 enhancement by probes, replicating our previous work. Bayesian analyses also show strong evidence against the hypothesis of N2 enhancement for the three components revealed by a temporal principal component analysis (PCA) conducted to disentangle potentially overlapping ERP effects. In conclusion, whereas the CIT has shown promise in detecting recognition of specific information, anterior N2 enhancement cannot be used as an electrophysiological measure of concealed information across CIT paradigms.

33. Keywords, separated by semicolons followed by space

Concealed information; ERPs; N2; recognition

Funding

34. Does the project have (or did it have) financial support from a funding agency?

yes

35. If this project is (was) being funded, please list grant numbers and funding agencies, separated by a semicolon and a space.

Research Executive Agency EuropeanUnion FP7 Marie Curie Initial TrainingNetworks (ITN), Grant/Award Number:FP7- PEOPLE- 2013- ITN- 604764;Research Executive Agency EuropeanUnion, Seventh Framework Programme(FP7), Marie Curie Career IntegrationGrant, Grant/Award Number: CIG09-GA- 2011- 293850- CoND

Ethical Approval

36. At the time of writing has the study been approved by an ethics committee?

yes

37. If the study has been approved by an ethics comittee, list the approval ID and institution which granted it. If there are multiple approvals, separate them by semicolons followed by space.

Universityof Plymouth Faculty of Health and Human SciencesResearch Ethics Committee

Acknowledgements

38. Do you have any acknowledgments to add in addition to authors or funding?

no

Non-applicable question 39 is skipped over.

How to cite

40. Do you have any instructions on how you (or the authors) want this study to be cited or otherwise acknowledged?

yes

41. Citation instructions

Hsu, C.-W., Schofield, T.,& Ganis, G. (2024). Anterior N2 enhancement isnot a general electrophysiological index ofconcealed information. Psychophysiology, 61,e14633. https://doi.org/10.1111/psyp.14633

2. Design and Sample

Sample

1. How many participants took (or will take) part in the study?

45

2. How many participants, in total, were included in the analysis, after exclusion of participants?

41

3. Specify reasons for excluding participants.

They had fewer than 50% trials in one or more stimulus categories due to incorrectly performing the task

4. Age range of the sample: age of the youngest participant

Answer not provided years

5. Age range of the sample: age of the oldest participant

Answer not provided years

6. Does the sample include clinical population?

no

Variables

7. Is the design of this study full factorial?

yes

8. How many between-subject factors does this study/pipeline have?

0

Non-applicable question 9 is skipped over.

10. How many experimental/comparison groups does the study feature in total?

1

Non-applicable questions 11 and 12 are skipped over.

13. How many within-subject factors does this study/pipeline have (excluding EEG factors)?

1

14. List within-subject factors with factor levels in the brackets, separated by semicolon

| *Item type (probe, irrelevant, target)*

15. How many within-subject conditions does the study have in total?

| *1*

Non-applicable question 16 is skipped over.

17. Does the study include an analysis of a relationship between ERPs and a non-ERP continuous variable (e.g., correlation of an ERP measure with IQ)

| *no*

Non-applicable question 18 is skipped over.

Presented trials

19. What determines the number of trials each participant was presented with?

| *fixed N of trials delivered*

20. The number of trials each participant is presented with (including repetition of the same stimuli or trials)

| *205*

21. How many trials does each condition feature (including repetition of the same stimuli or trials)?

| *135; 35; 35*

Non-applicable questions 22 to 28 are skipped over.

Analyzed trials

29. After eliminating trials on all bases (e.g., artifacts, behavioural errors), how many trials, on average, did you include for each condition per participant?

| *135; 33; 32*

30. The minimum number of trials analyzed per condition for any participant.

| *121; 29; 27*

31. The maximum number of trials analyzed per condition for any participant.

| *135; 35; 35*

Presenting software

32. Presenting software for the experimental task

| *Presentation*

33. Software details: version, in the case of the pre-existing software options, or the programming language and version if "custom script" was selected, or the software name and version if "other" was selected.

| *18.0*

3. Hardware

Preamp peripherals

1. How were the EEG electrodes placed (or will be placed)?

| *cap or net*

Non-applicable question 2 is skipped over.

3. Cap or net make (brand)

| *Biosemi*

Non-applicable question 4 is skipped over.

5. Cap or net model

| *Biosemi headcaps*

6. Make (brand) of the electrodes

| *Biosemi*

7. Model of electrodes

| *Biosemi Pin-Type Active-electrodes for the cap + flat active type for the loose electrodes*

8. Do the electrodes have pre-amplifiers?

| *yes (active electrodes)*

9. The material the electrodes are made of

| *Ag/Ag-Cl*

Non-applicable question 10 is skipped over.

11. Type of conductive medium used for electrodes

| *conductive gel*

Non-applicable question 12 is skipped over.

Electrode positioning

13. Did you (or will you) use all electrode slots available in the cap (i.e., they had electrodes in them)?

| *yes*

14. What type of placement convention is appropriate for describing electrode placement scheme in this study?

| *International 10-20 system and its extensions (e.g., 10-10, 5 percent)*

15. Which extension of the 10-20 system will you use to describe electrode positions?

| *10-10 convention*

Non-applicable question 16 is skipped over.

17. Number of EEG electrodes used for acquisition (including, if they are on scalp, ground and reference electrodes, as well as earlobes; excluding standalone EOG and other non-EEG electrodes).

| *36*

18. The appropriate methods description should also include a list or a scheme of all electrode locations. A convenient way to do this is currently not available in ARTEM-IS due to technical limitations, so please provide either a list of all electrodes, or a link where the sceheme/list can be found.

| *https://www.biosemi.com/pics/cap_32_layout_medium.jpg*

19. Did you (or will you) use any electrodes other than scalp electrodes and mastoids/earlobes?

| *no*

Non-applicable questions 20 and 21 are skipped over.

Signal Amplifiers

22. Make (brand) of the amplifier

> *Biosemi*

Non-applicable question 23 is skipped over.

24. Model of amplifiers

> *BioSemi Active-Two System*

Triggers

25. Source of triggers

> *presentation software trigger*

Non-applicable question 26 is skipped over.

27. Do you wish to describe triggers in more detail?

> *no*

Non-applicable questions 28 to 30 are skipped over.

Other peripheral devices

31. Other equipment used during acquisition

> *no*

Non-applicable question 32 is skipped over.

4. Acquisition

Acquisition software

1. Acquisition software

> *Biosemi*

2. Software details: version, in the case of the pre-existing software options, or the software name and version if "other" was selected.

> *Actiview 8.14*

Data quality

3. Did you (or do you plan to) document impedances, or an alternative data aquisition quality measure?

> *yes*

4. What is the appropriate acquisition data quality measure for the recording equipment?

> *a data quality measure alternative to impedances*

Non-applicable questions 5 to 7 are skipped over.

8. Report data quality measures provided by the recording system alternative to impedances.

> *Level of DC offset < 40 mV in absolute value (as recommended by Biosemi).*

Online (recording) reference

9. Was voltage measured, or will it be measured, relative to an online reference for EEG electrodes (as opposed to alternative solutions such as Driven Right Leg (DRL))?

> *no*

Non-applicable question 10 is skipped over.

11. Specify the alternative to an EEG online reference (for example DRL)

| *DRL*

Non-applicable questions 12 and 13 are skipped over.

14. Online reference for VEOG electrodes

| *the same reference as EEG electrodes*

Non-applicable question 15 is skipped over.

16. Online reference for HEOG electrodes

| *the same reference as EEG electrodes*

Non-applicable question 17 is skipped over.

18. Online reference for other non-EEG (and non-EOG) electrodes

| *no non-EEG non-EOG electrodes were used*

Non-applicable question 19 is skipped over.

Online filters

20. Which of the following online filters were used, or will be used?

| *low pass*

Non-applicable questions 21 to 26 are skipped over.

27. Type of online low-pass filter cut-off

| *half-power*

28. Low-pass filter cut-off used during recording

| *102.4 Hz*

29. Low-pass filter roll off used during recording

| *30 dB/octave*

30. Low-pass filter impulse response during recording

| *finite impulse response (FIR)*

31. Low-pass filter family during recording

| *other*

32. Low-pass filter family during recording other than listed above

| *5th-order Cascaded Integrator-Comb (CIC) decimation filter*

Non-applicable questions 33 to 39 are skipped over.

Sampling

40. Acquisition sampling rate

| *512 Hz*

Event code delay

41. Did you (or will you) need to account for the event code delay?

| *no*

Non-applicable questions 42 to 44 are skipped over.

5. Pre-processing

Pre-processing general

1. Is the same software used for all pre-processing steps?

> *yes*

2. Which software is used for pre-processing?

> *EEGlab (+ ERPlab)*

3. Software details: version, in the case of the pre-existing software options, or the programming language and version if "custom script" was selected, or the software name and version if "other" was selected.

> *EEGlab version 2022.0, ERPLab version 8.30*

The order of pre-processing steps

Step 1: Downnsampling

Step 2: Artifact elimination

Step 3: Other pre-processing step

Step 4: Offline filtering

Step 5: Artifact elimination

Step 6: Artifact elimination

Step 7: Epoching

Step 8: Artifact elimination

Step 9: Artifact elimination

Step 10: Artifact elimination

Step 11: Re-referencing

Pre-processing steps

Step 1. *Downnsampling*

Non-applicable questions 4 and 5 are skipped over.

6. New sampling frequency

> *250 Hz*

Step 2. *Artifact elimination*

7. Select artifact removal category

> *reject channels*

Non-applicable questions 8 to 18 are skipped over.

19. In this step, bad channels were (or will be) eliminated from:

> *from the entire recording of a participant*

20. Were there any channels that were eliminated for all participants?

> *no*

Non-applicable question 21 is skipped over.

22. Percentage of channels eliminated per participant on average in this step

> *5 %*

23. Criterion for eliminating bad channels in this step

> *pre-set numerical criterion/function*

Non-applicable questions 24 and 25 are skipped over.

26. Describe the numerical criterion/function for eliminating channels and provide numerical threshold(s) in this step

> *trimOutlier (SD threshold set at 75); Flat channels, using EEGLab pop_clean_rawdata function with "FlatlineCriterion" set to "5")*

Non-applicable questions 27 to 32 are skipped over.

Step 3. *Other pre-processing step*

33. Which pre-processing step other than listed did you, or will you, implement?

> *Detrending with PREP pipelins, "removeTrend*

Non-applicable questions 34 and 35 are skipped over.

36. Describe this step together with any parameters that are necessary to replicate it.

> *Answer not provided*

Step 4. *Offline filtering*

37. Which offline filters were (or will be) applied?

> *low pass*

Non-applicable questions 38 to 54 are skipped over.

55. Low-pass filter impulse rate

> *infinite impulse response (IIR)*

56. Low-pass filter family

> *Butterworth*

Non-applicable question 57 is skipped over.

58. Type of low-pass filter cut-off

> *half-amplitude*

59. Offline low-pass filter cut-off

> *40 Hz*

60. Offline low-pass filter roll off

> *20 dB/octave*

61. Do you want to report any additional properties of the low-pass filter?

> *yes*

62. Which of these other additional offline low-pass filter properties you want to report?

> *filter direction, filter order*

Non-applicable questions 63 to 65 are skipped over.

66. Direction of the offline low-pass filter

> *forwards and backwards*

Non-applicable question 67 is skipped over.

68. Low-pass filter order

> *8*

Non-applicable questions 69 to 76 are skipped over.

Step 5. *Artifact elimination*

77. Select artifact removal category

> *reject bad trial or bad data segment on all channels*

Non-applicable questions 78 and 79 are skipped over.

80. Is rejection applied to trials or segments of continuous data in this step?

> *data segments*

81. How are artifacts defined in this step?

> *pre-set numerical criterion/function*

Non-applicable questions 82 and 83 are skipped over.

84. Describe the numerical criterion/function and provide numerical threshold(s) in this step

> *Removal of segments containing data at anterior frontal chans with amp > |300| µV or peak-to-peak amp > |600| µV (500 ms window, moved in 250ms steps)*

Non-applicable questions 85 to 102 are skipped over.

Step 6. *Artifact elimination*

103. Select artifact removal category

> *reject bad trial or bad data segment on all channels*

Non-applicable questions 104 and 105 are skipped over.

106. Is rejection applied to trials or segments of continuous data in this step?

> *data segments*

107. How are artifacts defined in this step?

> *pre-set numerical criterion/function*

Non-applicable questions 108 and 109 are skipped over.

110. Describe the numerical criterion/function and provide numerical threshold(s) in this step

> *Removal of segments at any other channels (other than anterior frontal) with amp > |225uV| or p2p amp > |450|uV (500 ms window, moved in 250ms steps)*

Non-applicable questions 111 to 128 are skipped over.

Step 7. *Epoching*

Non-applicable questions 129 and 130 are skipped over.

131. Time-locking event relative to which epochs are defined

> *stimulus onset*

Non-applicable question 132 is skipped over.

133. Epoch beginning relative to the time-locking point

> *-200 ms*

134. Epoch end relative to the time-locking point

> *1000 ms*

135. Does the software baseline-correct data in the same preprocessing step as epoching? If yes, add

"Baseline correction" as the next step in the preprocessing pipeline to describe it.

> *yes*

Step 8. *Artifact elimination*

136. Select artifact removal category

> *correct by subtraction (blinks, EKG etc.)*

Non-applicable questions 137 to 144 are skipped over.

145. General category of artifact correction approach in this step

> *ICA-based approaches*

146. Reference to the artifact correction algorithm used in this step

> *EEGlab pop_runica function with infomax option and default parameters. ICLabel for component removal.*

147. Describe artifact correction procedure in detail sufficient for replication.

> *IcLabel with a threshold of 0.90 for the probability of being a muscular, cardiac, or channel artifact; a component was identified as an ocular artifact and removed if the corresponding label weight was the largest and the brain weight was < 0.05 (i.e., if it was estimated to contain less than 5% brain signals)*

Non-applicable questions 148 to 161 are skipped over.

Step 9. *Artifact elimination*

162. Select artifact removal category

> *reject bad trial or bad data segment on all channels*

Non-applicable questions 163 and 164 are skipped over.

165. Is rejection applied to trials or segments of continuous data in this step?

> *trials*

166. How are artifacts defined in this step?

> *pre-set numerical criterion/function*

Non-applicable questions 167 and 168 are skipped over.

169. Describe the numerical criterion/function and provide numerical threshold(s) in this step

> *removal of any residual trials with vertical or horizontal electrooculographic amplitude greaterthan 100 or 50 µV (in absolute value), respectively, as wellas of trials with an incorrect response;*

Non-applicable questions 170 to 187 are skipped over.

Step 10. *Artifact elimination*

188. Select artifact removal category

> *interpolate channels*

Non-applicable questions 189 to 208 are skipped over.

209. How did you (or will you) select channels for interpolation in this step?

> *Those removed in step 2*

210. Describe interpolation method and its settings.

> *EEGlab pop_interp function, with 'spherical' option*

Non-applicable questions 211 to 213 are skipped over.

Step 11. *Re-referencing*

Non-applicable questions 214 and 215 are skipped over.

216. Data re-referenced in this step

> *EEG*

Non-applicable questions 217 to 224 are skipped over.

Order of operations

225. Do you want to additionally self-describe the order of preprocessing operations?

> *no*

Non-applicable question 226 is skipped over.

6. Channels

Selection of channels for analysis

1. Which channels were (or will be) analyzed?

> *a subset of channels submitted to statistical analysis*

2. Rationale for selecting this exact subset of channels

> *a priori*

3. A priori method of channel selection

> *The same as in previous literature*

4. Literature references, separated by semicolon and space

> *Ganis, G., Bridges, D., Hsu, C. W., & Schendan, H. E. (2016). Is an-terior N2 enhancement a reliable electrophysiological index ofconcealed information? NeuroImage, 143, 152–165, DOI: https://doi.org/10.1016/j.neuroimage.2016.08.042*

Non-applicable questions 5 to 7 are skipped over.

8. Number of a priori selected channels

> *1*

9. List of a priori selected channels, separated by a semicolon and a space

> *Fz*

Non-applicable questions 10 to 28 are skipped over.

Channel aggregating

29. Are channels used individually or aggregated into regions of interest when doing component measurement and/or statistical analysis

> *individual channels*

Non-applicable questions 30 to 32 are skipped over.

7. Measurements

Measurement

1. Does this study include measuring amplitude and/or latency for the purposes of later statistical analysis?

> *yes*

2. Measure type(s)

amplitude

Amplitude measurement - software

3. Amplitude measurement software

EEGlab (+ ERPlab)

4. Software details: version, in the case of the pre-existing software options, or the programming language if "custom script" was selected, or the software name and version if "other" was selected.

As before

Amplitude measurement - waveforms

5. The amplitude is measured from

difference waves

6. Which conditions are subtracted from which? Specify how difference waves were, or will be, constructed.

Probe - Irrelevant

Non-applicable question 7 is skipped over.

Amplitude measurement - measure

8. Select procedure(s) used for amplitude measurement in this time window.

mean over a time window

Non-applicable questions 9 to 16 are skipped over.

Amplitude measurement - time window

17. Strategy for measurement time window selection

a priori

18. Select the a priori procedure (to be) used for amplitude measurement in this time window.

The same as in previous literature

19. Literature references, separated by semicolon and space

Ganis, G., Bridges, D., Hsu, C. W., & Schendan, H. E. (2016). Is an-terior N2 enhancement a reliable electrophysiological index ofconcealed information? NeuroImage, 143, 152–165, DOI: https://doi.org/10.1016/j.neuroimage.2016.08.042

Non-applicable questions 20 to 22 are skipped over.

23. Start of the a priori selected time window for amplitude measurement

250 ms

24. End of the a priori selected time window for amplitude measurement

300 ms

Non-applicable questions 25 to 81 are skipped over.

8. Visualization

Plot type

1. How were ERPs visualised (or how will they be visualised)? Hover over the help sign for the

visualisation naming convention.

> *ERP plot (line plot), topoplot and/or topoplot time series*

Line plot

2. Extra preprocessing: Have any procedures been applied for line plot visualisation purposes only, such as smoothing filter or a different baseline not used in the analyses (or will they be applied, for future studies)?

> *no*

Non-applicable question 3 is skipped over.

4. What do the waveforms represent?

> *single conditions*

5. Which single conditions are shown?

> *Probe; Irrelevant*

Non-applicable questions 6 and 7 are skipped over.

8. Are all channels or a selection of channels used for the visualisation?

> *selected channels*

9. List of channels selected for this visualisation (including those that are averaged together)

> *Fz*

10. What is the rationale for showing these channels?

> *This study was a replication of a previous study and focused on the N2 at Fz.*

11. Are the channels shown individually or averaged?

> *individual channels shown*

Non-applicable questions 12 and 13 are skipped over.

14. Beginning of the time window which the ERP plot shows (relative to trigger)

> *-200 ms*

15. End of the time window which the ERP plot shows (relative to trigger)

> *400 ms*

16. What is the rationale for selecting this time window?

> *other*

17. Rationale for selecting this time window

> *The frontal N2 time window was 250-300 ms, and so the main figure showed ERPs between -200 and 400 ms to have some context. The time window for the PCA analyses (not reported here) was -200 1000ms.*

18. Does the ERP plot include some kind of representation of uncertainty or error margins for waveforms?

> *no*

Non-applicable questions 19 and 20 are skipped over.

21. Do you want to add additional information about this plot?

> *no*

Non-applicable question 22 is skipped over.

Topoplot

23. Extra preprocessing: Have any procedures been applied for topoplot visualisation purposes only, such as smoothing filter or a different baseline not used in the analyses (or will they be applied, for future studies)?

> *no*

Non-applicable question 24 is skipped over.

25. What do the waveforms represent?

> *both single and difference wave conditions*

26. Which conditions are shown?

> *Probe; Irrelevant*

27. Which difference waves are shown?

> *Probe - Irrelevant*

Non-applicable question 28 is skipped over.

29. What kind of unit does the topoplot have?

> *voltage topoplot*

Non-applicable question 30 is skipped over.

31. Are all channels or a selection of channels visualised in this topoplot?

> *all channels*

Non-applicable questions 32 and 33 are skipped over.

34. Are the topoplots averaged over a single time window?

> *yes*

35. Beginning of the time window selected for this visualisation (relative to the time-locking point)

> *250 ms*

36. End of the time window selected for this visualisation (relative to the time-locking point)

> *300 ms*

37. Rationale for selecting this time window

> *Previous literature*

38. Are there topoplots representing a time series of multipe time windows?

> *no*

Non-applicable questions 39 and 40 are skipped over.

41. Do you want to add additional information about this plot?

> *yes*

42. Any additional relevant information you may want to include about this plot

> *There were also plots of PCA timecourses and PCA topographic maps (not described here as they were not the main analysis).*

Non-applicable questions 43 to 76 are skipped over.

9. Other

Additional comments

1. Do you have any additional information or comments to add?

> *no*

Non-applicable question 2 is skipped over.

Contributor(s)

Giorgio

License

This report has been downloaded under the CC BY 4.0 license on October 28, 2025.
https://artemis.incf.org/202410271633514mTakC
